# Microglial Membranes Wrapped Ultrasmall Medium‐Entropy Ru Single‐Atom Nanozyme: Enhanced Catalysis for Accelerating Inflammation/Redox Microenvironment Regulation in Intracerebral Hemorrhage

**DOI:** 10.1002/advs.202520714

**Published:** 2026-01-04

**Authors:** Jiebo Li, Penghui Wei, Yuanbo Pan, Hongjia Zheng, Jiajun Hu, Qianxi Chen, Zhongyuan Shen, Yongrui Hu, Jiajun Wu, Fuxin Lin, Fuxiang Chen, Chenyu Ding, Wenhua Fang, Yuanxiang Lin, Dezhi Kang, Yuxiang Gu, Yang Zhu, Dengliang Wang

**Affiliations:** ^1^ Department of Neurosurgery Neurosurgery Research Institute The First Affiliated Hospital Fujian Medical University Fuzhou Fujian China; ^2^ Department of Neurosurgery National Regional Medical Center Binhai Campus of the First Affiliated Hospital Fujian Medical University Fuzhou Fujian China; ^3^ Fujian Provincial Institutes of Brain Disorders and Brain Sciences The First Affiliated Hospital Fujian Medical University Fuzhou Fujian China; ^4^ Fujian Provincial Clinical Research Center for Neurological Diseases First Affiliated Hospital Fujian Medical University Fuzhou Fujian China; ^5^ Department of Neurosurgery School of Medicine Second Affiliated Hospital Zhejiang University Hangzhou China

**Keywords:** catalytic therapy, ferroptosis, intracerebral hemorrhage, medium‐entropy, single‐atom nanozyme

## Abstract

Intracerebral hemorrhage (ICH) causes severe secondary brain injury (SBI) via excessive inflammation and reactive oxygen species (ROS), and current treatments lack effective dual‐target efficacy. In this study, we designed a microglial membrane‐wrapped single‐atom nanozyme (PtRhIr/Ru SAN@M) by anchoring a single atom of Ru onto ultrasmall, medium‐entropy PtRhIr alloys. This design integrates two underutilized strategies, single‐atom nanozymes (SANs) and medium‐entropy catalysts, to address critical therapeutic gaps in ICH therapy. PtRhIr/Ru SAN@M exhibited enhanced catalytic activity with superior Hydroxyl radical (•OH) scavenging and superoxide dismutase (SOD)‐like and catalase (CAT)‐like performances compared to Ru‐free PtRhIr@M, enabled by electronically modulated active sites. Fluorescence imaging confirmed its ability to penetrate the blood‐brain barrier (BBB) and accumulate in post‐ICH neuroinflammatory regions. Both in vitro and in vivo experiments demonstrated that PtRhIr/Ru SAN@M repolarized microglia from the M_1_ to the M_2_ phenotype, disrupting the neuroinflammatory cycle and halting neuronal death. Therapeutic intervention with PtRhIr/Ru SAN@M significantly increased survival rates, restored neurological function, and enhanced spatial memory after ICH. This study pioneers the integration of SANs with medium‐entropy alloys for ICH, offering a dual‐target ROS‐inflammation regulatory strategy and a generalizable platform for ROS‐related degenerative disease therapies.

## Graphical abstract

PtRhIr/Ru SAN@M nanozymes cross the blood–brain barrier and selectively accumulate in hemorrhagic brain regions. By mimicking multiple enzyme activities, they attenuate oxidative stress, modulate microglial polarization toward an anti‐inflammatory phenotype, inhibit ferroptosis, and promote neuronal repair. This multifunctional approach offers a targeted therapeutic strategy to restore neurological function and improve recovery in intracerebral hemorrhage.

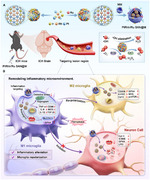

## 1 Introduction

Cerebrovascular disease remains a major cause of mortality and long‐term disability worldwide. Intracerebral hemorrhage (ICH), a life‐threatening subtype of stroke, often leads to severe neurological impairment or death [1, 2, 3, 4]. The pathogenesis of ICH not only involves primary brain injury from blood vessel rupture and hematoma compression but also relies heavily on subsequent inflammation and reactive oxygen species (ROS) overproduction: the extravasated blood releases hemoglobin and pro‐inflammatory factors to recruit microglia and neutrophils, triggering a robust inflammatory response [1,5, 6, 7]; meanwhile, hemoglobin degradation and oxidative stress induce excessive ROS generation, which exacerbates neuronal lipid peroxidation (LPO), protein oxidation, and DNA damage. Together, these processes collectively amplify secondary brain injury (SBI) and impede neurological recovery [8, 9, 10]. Currently, the clinical management of ICH‐related inflammation and ROS damage primarily relies on supportive interventions, mainly including anti‐inflammatory agents (e.g., corticosteroids for severe inflammation), antioxidants (e.g., vitamin C/E supplements), and neuroprotective drugs to mitigate oxidative stress and inflammatory cascades, while therapies that simultaneously target both pathways remain limited [4,11, 12]. In addition, surgical intervention for ICH often induces severe postoperative SBI, which substantially compromises the overall therapeutic efficacy [13, 14, 15]. Given the inherent limitations of current treatments, there is an urgent and unmet clinical need to develop novel therapeutic agents equipped with broad‐spectrum ROS‐scavenging capacity and potent anti‐inflammatory properties for effective and targeted management of ICH [16, 17].

Nanozymes, an emerging class of nanomaterials with intrinsic enzyme‐mimetic activities, have attracted increasing research interest owing to their prominent advantages, including high stability, tunable catalytic activity, and cost‐effectiveness [18, 19, 20, 21, 22, 23, 24]. As a novel type of biocatalyst, metal‐based nanozymes exhibit the inherent ability to regulate ROS generation and scavenging, enabling their widespread application in the biomedical field [25, 26, 27, 28]. Recent studies have demonstrated that nanozymes hold considerable potential for ICH treatment [18, 29]; however, their further development and clinical translation are constrained by critical challenges, such as the structural complexity of nanomaterials and suboptimal atomic utilization efficiency. Recent advances in next‐generation single‐atom nanozymes (SANs) represent a transformative breakthrough. SANs offer enhanced biomedical applicability via tunable electronic properties and maximized atomic utilization efficiency [30,31], making them promising candidates for ICH therapy [32]. Beyond SANs, multimetallic alloy nanostructures, including medium‐ and high‐entropy nanozymes, have garnered widespread research attention [33, 34, 35, 36, 37]. Although medium‐entropy catalysts typically contain fewer elements (3–4 in near‐equimolar ratios) compared to high‐entropy catalysts (≥ 5 elements), they still exhibit extensive modifiability and unique physicochemical properties, such as significant electronic structure modulation and strong synergistic effects between components [33,38, 39]. Regrettably, the potential of integrating SANs with medium‐entropy catalysts for ROS elimination in ICH treatment remains largely unexplored, representing a critical gap in current ICH therapeutic strategies [33,34, 40]. This uncharted territory represents a compelling opportunity for future research, as it could unlock new strategies for mitigating oxidative stress and neuroinflammation associated with ICH, ultimately leading to improved therapeutic outcomes [34, 35, 36].

**SCHEME 1:**
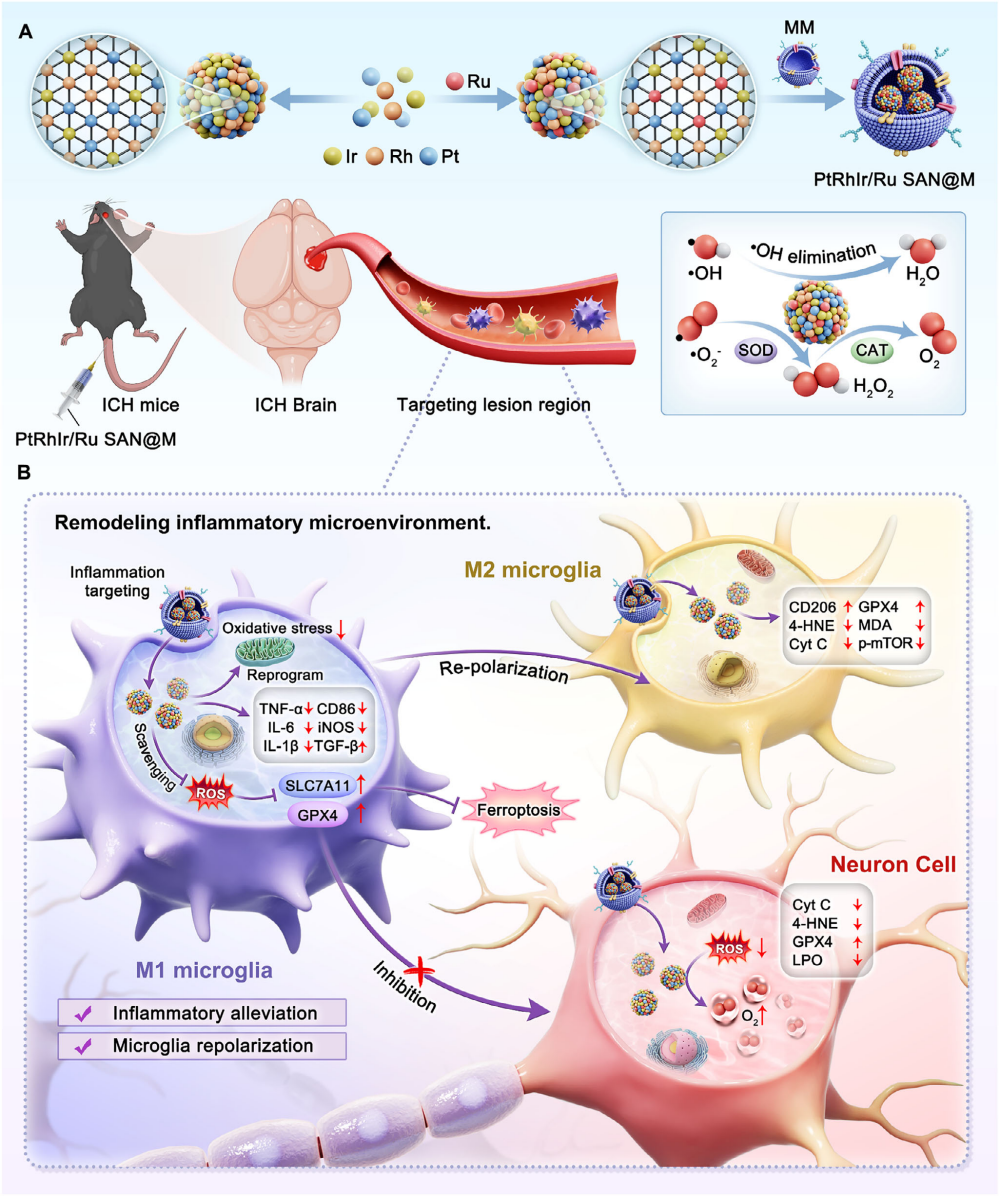
Schematic illustration of the fabrication of PtRhIr/Ru SAN@M and its cascade‐targeting anti‐inflammatory and antioxidative therapy for ich.

Herein, we present a microglial membrane‐wrapped nanozyme constructed by anchoring single‐atomic ruthenium (Ru) onto an ultrasmall medium‐entropy PtRhIr alloy nanostructure, denoted as PtRhIr/Ru SAN@M (Scheme 1) [33, 34]. This innovative design significantly enhances ROS elimination, laying a solid foundation for effective ICH treatment [41, 42, 43]. Owing to the modulation of the electronic structure, PtRhIr/Ru SAN@M showed markedly superior hydroxyl radical (•OH) scavenging capability as well as superoxide dismutase (SOD)‐like and catalase (CAT)‐like activities compared to its Ru‐free counterpart (PtRhIr@M). Fluorescence imaging with Cy5.5‐labeled PtRhIr/Ru SAN@M confirmed two key biological properties: intrinsic blood‐brain barrier (BBB) penetration ability and targeted accumulation within neuroinflammatory foci of post‐ICH brains. Both in vitro and in vivo experiments demonstrated that PtRhIr/Ru SAN@M potently repolarized pro‐inflammatory M_1_ microglia toward the reparative M_2_ phenotype. This repolarization disrupts the self‐perpetuating cycle of neuroinflammation and halts progressive neuronal death, which are two critical processes in mitigating ICH‐induced SBI. Ultimately, therapeutic intervention with PtRhIr/Ru SAN@M achieved three pivotal outcomes post‐ICH: a significant increase in survival rate, substantial restoration of neurological function, and marked enhancement of spatial memory. This study established an efficient strategy for anchoring isolated single atoms to boost nanozyme catalytic activity, while also providing a promising therapeutic approach for ROS‐related neurodegenerative diseases beyond ICH.

## 2 Results and Discussion

### 2.1 Preparation and Characterization of PtRhIr and PtRhIr/Ru SAN

**FIGURE 1:**
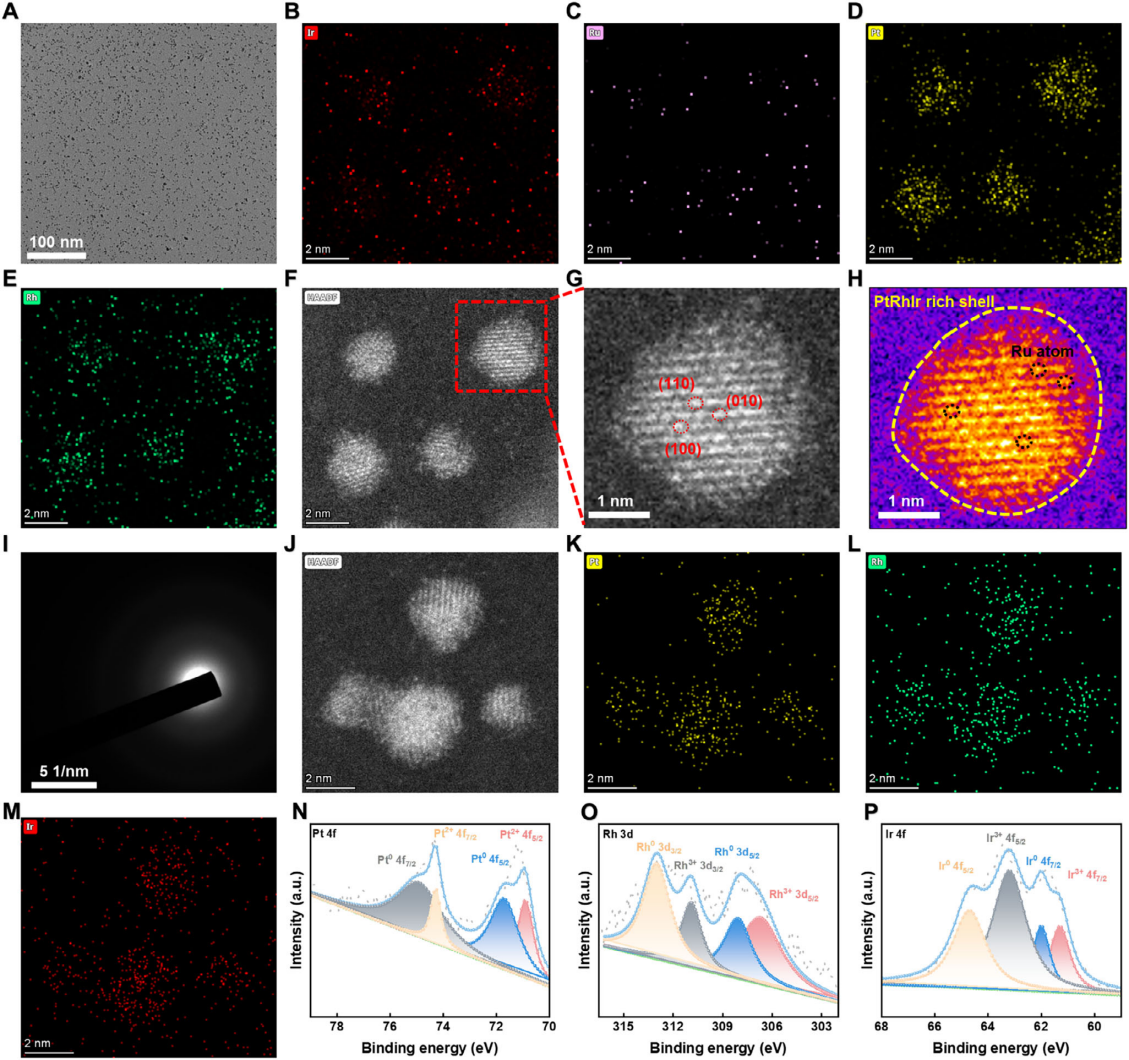
Construction and characterization of PtRhIr/Ru SAN. (A) TEM characterization image of PtRhIr/Ru SAN. (B–E) EDS‐mapping images of PtRhIr/Ru SAN. (F–G) HAADF image of PtRhIr/Ru SAN. (H) HAADF‐STEM images and corresponding with elemental mapping of PtRhIr/Ru SAN. (I) The selected area electron diffraction pattern of PtRhIr/Ru SAN. (J–M) HAADF image and corresponding mapping image of PtRhIr nanozyme. (N–P) High‐resolution XPS spectra of pt 4f, Rh 3d, and Ir 4f in PtRhIr/Ru SAN.

In a meticulously controlled synthesis process, precisely weighed amounts of Potassium hexachlororhodate (III) (0.2 mmol), Potassium tetrachloroplatinate (II) (0.2 mmol), Iridium (III) chloride (0.2 mmol), and Ruthenium (III) chloride (0.01 mmol) were dissolved together in 20 mL of ultrapure water. This solution was then enriched with 100 mg of polyvinylpyrrolidone K30 and magnetically stirred for 30 min to ensure thorough mixing and stabilization of the precursor solution. Subsequently, 4 mL of a freshly prepared 10 mm sodium borohydride solution was introduced, initiating a reduction reaction that was carefully maintained at 30°C for 2 h. These reaction conditions were specifically chosen to optimize the reduction kinetics and precisely control the nucleation and growth of the nanostructures. The resulting product was collected via ultrafiltration at 6000 rpm for 30 min, followed by three washes with 15 mL of ultrapure water to remove any residual reactants and impurities, yielding a high‐purity PtRhIr/Ru SAN. To ensure stability and reproducibility, both PtRhIr/Ru SAN and PtRhIr/Ru SAN@M were stored at 4°C under light‐protected conditions. Transmission electron microscopy (TEM) imaging, a powerful tool for probing the morphology and size of nanomaterials, revealed that the PtRhIr/Ru SAN exhibited spherical particles with a highly uniform average diameter of 2.2 ± 0.3 nm (Figure 1A; Figure S1). This uniformity is crucial for ensuring consistent catalytic performance and stability of the nanozyme. Energy dispersive X‐ray spectrometry (EDS) mapping, which provides nanoscale elemental distribution, confirmed the presence of Ir, Rh, Pt, and Ru within the PtRhIr/Ru SAN (Figure 1B–E). This elemental composition is essential for the synergistic catalytic activity and unique properties of nanozymes. High‐angle annular dark‐field scanning TEM (HAADF‐STEM), a state‐of‐the‐art technique for visualizing atomic‐scale structures, further elucidated the intricate architecture of the PtRhIr/Ru SAN nanozymes (Figure 1F,G). These images not only confirmed the presence of mesoporous channels within the nanozyme but also revealed a spherical structure with distinct compositional contrast. The yellow dashed outline in Figure 1H highlights the PtRhIr‐rich shell, whereas the dotted circles indicate the isolated Ru atoms in the core. This unique core–shell structure, with Pt, Rh, and Ir concentrated in the shell and Ru existing as single atoms in the inner region, was a key determinant contributing to the enhanced catalytic properties of the PtRhIr/Ru SAN. The selected area electron diffraction pattern (Figure 1I) displayed distinct diffraction rings, providing clear evidence of the crystalline order and structure of the PtRhIr/Ru SAN. Such crystallinity is critical for catalytic activity and stability. Additional HAADF‐STEM imaging (Figure 1J) and EDS elemental mapping (Figure 1K–M) were employed to further corroborate the spatial distribution and structural characteristics of the multimetallic components within the PtRhIr/Ru SAN system, ensuring a comprehensive structural characterization. To further investigate the crystal structure, X‐ray diffraction (XRD) analysis was performed. The XRD patterns of both the PtRhIr nanozyme and the PtRhIr/Ru SAN (Figure S2) exhibited well‐defined, distinct peaks, confirming their crystalline structures. The subtle peak shifts and intensity differences observed in the patterns suggested variations in atomic arrangement and metal composition, which are key determinants of the catalytic properties of the nanozymes. These variations highlight the importance of precise synthetic control to tailor nanozyme properties for specific biomedical applications.

Additionally, the PtRhIr/Ru SAN@M was prepared by co‐extruding microglial membranes (M) and PtRhIr/Ru SAN through 1 µm and 450 nm porous membranes, respectively. This approach aimed to integrate nanozymes into biological membranes to enhance their biocompatibility and functionality. To verify the successful integration and functionality of the microglial membranes, the protein content of PtRhIr/Ru SAN@M was analyzed using sodium dodecyl sulfate‐polyacrylamide gel electrophoresis. Gel electrophoresis and protein staining revealed no significant differences between the protein profiles of the purified microglial membranes and PtRhIr/Ru SAN@M (Figure S3), indicating that the nanozymes were successfully encapsulated within the membranes without disrupting membrane protein composition. The zeta potentials of the PtRhIr/Ru SAN and PtRhIr nanozymes were slightly negative (Figure S4), indicating good colloidal stability in dispersion. PtRhIr/Ru SAN demonstrated excellent colloidal stability, as reflected by stable hydrodynamic diameter (Figure S5A) and unchanged Ru content (Figure S5B) over time in physiological media. In summary, these findings confirm the successful fabrication of medium‐entropy PtRhIr/Ru SAN and PtRhIr nanozymes. Comprehensive physicochemical characterization provides a solid foundation for understanding their unique structural features and catalytic properties, paving the way for further exploration of their potential applications in biomedicine and catalytic therapy.

**FIGURE 2:**
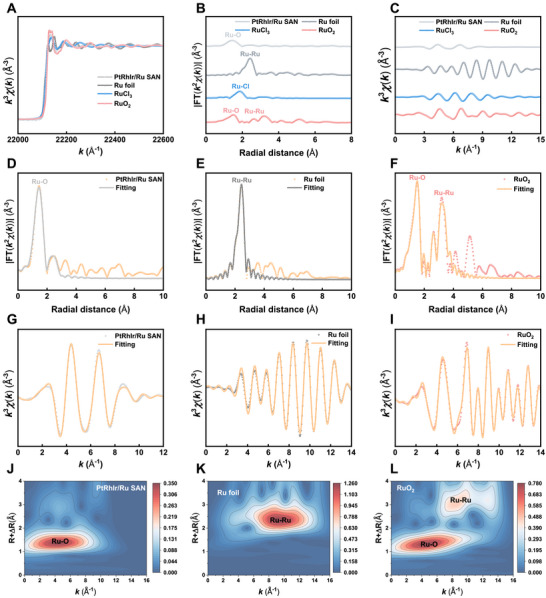
Atomic structural analysis of PtRhir/Ru SAN. (A) XANES spectra of PtRhIr/Ru SAN. (B) EXAFS of the PtRhIr/Ru SAN. (C) EXAFS curves of PtRhIr/Ru SAN in the k space. (D) EXAFS fitting curves of PtRhIr/Ru SAN, (E) Ru foil, and (F) Ruo_2_ at the r space. (G) EXAFS fitting curve of PtRhIr/Ru SAN, (H) Ru foil, and (I) Ruo_2_ at the k space. (J) Wavelet transformation of PtRhIr/Ru SAN, (K) Ru foil, and (L) Ruo_2_.

The chemical composition and electronic structure of the PtRhIr/Ru SAN were analyzed using X‐ray photoelectron spectroscopy (XPS). Pt, Rh, Ir, and Ru were identified as the main elements (Figure 1N–P). High‐resolution XPS confirmed that Ru existed primarily as an atomically dispersed species (Figure S6). Additionally, clear spin‐orbit splitting of Ru 3p (Ru 3p_3/2_ and Ru 3p_1/2_) was observed, further validating the incorporation of Ru into the nanozyme. To elucidate the local coordination environment of Ru, synchrotron radiation–based X‐ray absorption spectroscopy was employed, including both X‐ray absorption near‐edge structure (XANES) and extended X‐ray absorption fine structure (EXAFS) analyses. The Ru K‐edge XANES spectra displayed a distinct absorption edge shift (Figure 2A), confirming the Ru^δ+^ oxidation state, which was consistent with the XPS results. Furthermore, the Fourier transform of the EXAFS data in R‐space revealed a prominent peak at 1.45 Å, characteristic of the Ru─O bond. Notably, the absence of any peaks at 2.39 Å, which would indicate Ru─Ru bonding, is particularly significant. These observations collectively demonstrate the atomic dispersion of Ru active sites within the PtRhIr/Ru SAN (Figure 2B–F). EXAFS fitting spectra at the Ru K‐edge revealed that Ru atoms in PtRhIr/Ru SAN were predominantly coordinated with four nitrogen atoms (Figure 2G–I; Figure S7, and Table S1). Additionally, the PtRhIr/Ru SAN exhibited a wavelet transform signal at 5.0 Å^−1^, corresponding to the Ru─O bond, while no wavelet transform intensity attributable to Ru─Ru bonds was detected (Figure 2J–M). Collectively, these findings provide conclusive evidence for the successful synthesis of atomically dispersed PtRhIr/Ru SAN.

### 2.2 Multi‐Enzyme‐Mimetic Activities of PtRhIr/Ru SAN

**FIGURE 3:**
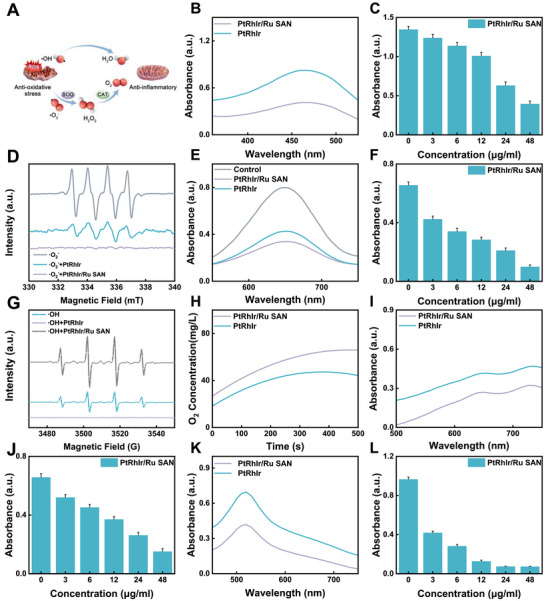
Multiple enzyme‐like activities of PtRhIr/Ru SAN in vitro. (A) multiple enzyme‐like activities of PtRhIr/Ru SAN. (B) sod‐like ability of nanozymes. (C) sod‐like activity at various concentrations (n = 3, for each group). (D) ·o_2_^−^ scavenging property by ESR. (E) •OH scavenging ability of nanozymes measured by TMB. (F) •OH scavenging ability at various concentrations (n = 3, for each group). (G) •OH scavenging property by ESR. (H) cat‐like ability of nanozymes. (I) ABTS‐like ability of nanozymes. (J) ABTS‐like activity at various concentrations (n = 3, for each group). (K) DPPH‐like ability of nanozymes. (L) DPPH‐like activity at various concentrations (n = 3, for each group). All experiments were expressed as mean ± S.D.

The multienzyme‐mimetic activities of the PtRhIr/Ru SAN were systematically characterized, and the catalytic mechanism is schematically presented in Figure 3A. As a core component of the antioxidant defense system, SOD mediates the dismutation of superoxide anions (•O_2_^−^) into hydrogen peroxide via a redox cycle [44,45]; thus, we first evaluated the SOD‐like activity of the PtRhIr/Ru SAN using a xanthine oxidase‐based assay with water‐soluble tetrazolium salt‐8 (WST‐8) as the chromogenic indicator (•O_2_–responsive). Absorbance measurements at 420 nm, the characteristic absorption wavelength of the WST‐8 reaction product, showed that PtRhIr/Ru SAN exhibited a significantly higher •O_2_^−^ scavenging efficiency than the PtRhIr alloy nanozyme (Figure 3B), confirming its robust •O_2_^−^ removal capability (Figure 3C; Figure S8). To further validate this at the radical level, electron spin resonance (ESR) spectroscopy was performed after catalysis (Figure 3D). The ESR signal intensity of •O_2_^−^ was markedly reduced in the PtRhIr/Ru SAN group compared with that in PtRhIr, providing direct evidence of enhanced •O_2_^−^ scavenging activity.

•OH‐scavenging activity was assessed using 3,3′,5,5′‐tetramethylbenzidine (TMB) as a chromogenic probe. In the presence of H_2_O_2_, PtRhIr/Ru SAN catalyzed the oxidation of colorless TMB to blue‐oxidized TMB with a maximum absorption at 625 nm, which is associated with •OH‐mediated TMB oxidation [38, 46], indicating that PtRhIr/Ru SAN converts •OH into non‐toxic H_2_O during the reaction. Quantitative analysis showed that PtRhIr/Ru SAN exhibited a stronger •OH‐scavenging capacity than PtRhIr (Figure 3E), and its enzyme‐mimetic activity increased in a concentration‐dependent manner (Figure 3F; Figure S9). ESR spectroscopy further verified the •OH scavenging dynamics (Figure 3G). The characteristic ESR signal of •OH was significantly attenuated in the presence of PtRhIr/Ru SAN, directly confirming its efficient •OH elimination capability.

CAT‐like activity, mediating H_2_O_2_ decomposition into O_2_ and H_2_O, was evaluated by monitoring in situ O_2_ generation using a portable dissolved oxygen meter (Figure 3H; Figure S10). PtRhIr/Ru SAN outperformed several reported nanozymes for ferroptosis‐related therapy, including CeO_2_, CuO, and Mn_3_O_4_, in terms of O_2_ production, demonstrating its superior catalytic efficiency (Figure S11). Upon exposure to H_2_O_2_, PtRhIr/Ru SAN rapidly generated O_2_, attributable to its large specific surface area and abundant active sites. At a nanozyme concentration of 12 µg/mL, the O_2_ concentration reached 36 mg/L within 60 s and further increased to 65 mg/L at 500 s. In contrast, PtRhIr exhibited a markedly slower O_2_ generation rate, with the concentration peaking only after ≈400 s, highlighting the superior CAT‐like catalytic kinetics of PtRhIr/Ru SAN.

We further extended the radical scavenging evaluation to 2,2′‐azinobis(3‐ethylbenzothiazoline‐6‐sulfonic acid) (ABTS) and 2,2‐diphenyl‐1‐picrylhydrazyl (DPPH) radicals, which are commonly used models for assessing broad‐spectrum antioxidant capacity. For ABTS radical scavenging, UV–vis absorption spectra showed a progressive decrease in the characteristic ABTS radical absorption peak (Figure 3I), indicating effective radical consumption; concentration‐dependent analysis confirmed that ABTS scavenging activity increased with PtRhIr/Ru SAN concentration, reflecting a dose‐dependent trend (Figure 3J; Figure S12). Similarly, DPPH radical scavenging was validated via UV–vis spectroscopy (Figure 3K). The attenuation of the characteristic absorption peak, supported by radical elimination and concentration‐dependent experiments, demonstrated that PtRhIr/Ru SAN scavenged DPPH radicals in a dose‐dependent manner (Figure 3L; Figure S13). Collectively, these results demonstrate that the incorporation of Ru single atoms significantly enhances the enzyme‐mimetic activity of the PtRhIr alloy and that the PtRhIr/Ru SAN integrates SOD‐like, •OH‐scavenging, CAT‐like, ABTS‐scavenging, and DPPH‐scavenging functions, forming a broad‐spectrum ROS‐scavenging system. This multienzyme mimetic property enables effective inhibition or alleviation of ROS‐induced oxidative damage in biological systems, thereby laying a critical foundation for its in vivo therapeutic application.

### 2.3 In Vitro Evaluation of PtRhIr/Ru SAN@M In Hemin‐Induced Ich Model

**FIGURE 4:**
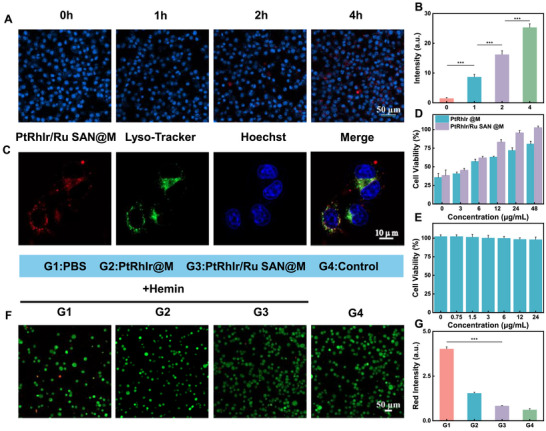
Effect of the nanozyme system on cell viability. (A) CLSM images of PtRhIr/Ru SAN@M showing cellular uptake in BV2 cells and (B) the corresponding quantification of fluorescence intensity (n = 3, for each group). (C) CLSM images of colocalization between the lysosome tracker and PtRhIr/Ru SAN@M in BV2 cells. (D) cell viability of hemin‐induced BV‐2 cells following 24 h treatment with various concentrations of PtRhIr@M and PtRhIr/Ru SAN@M (n = 4, for each group). (E) cell viability of BV2 cells treated with different concentrations of PtRhIr/Ru SAN@M (n = 4, for each group). (F) CLSM images depicting calcein‐am/pi co‐staining of BV2 cells with different treatments and (g) the corresponding quantification of fluorescence intensity (n = 3, for each group). Statistical significance was assessed using a two tailed student's *t*‐test. Data are presented as means ± SD. ^*^ *p* < 0.05, ^**^ *p* < 0.01, ^***^ *p* < 0.001.

Hemin, an iron‐containing porphyrin and potent inducer of heme oxygenase‐1, was chosen to establish an in vitro ICH model [47, 48]. To evaluate the protective effects of PtRhIr/Ru SAN@M against hemin‐induced cellular injury, we first assessed its uptake by BV2 microglial cells using confocal laser scanning microscopy (CLSM). The results demonstrated a time‐dependent increase in the red fluorescence signal of PtRhIr/Ru SAN@M within the cells (Figure 4A,B; Figure S14), reflecting its high cellular affinity and efficient intracellular internalization, which are key prerequisites for its protective effects. Further colocalization experiments confirmed that PtRhIr/Ru SAN@M escaped lysosomal sequestration (Figure 4C), suggesting its effective cytosolic availability and potential intracellular site of action. Cell Counting Kit‐8 (CCK‐8) assays were used to assess both cytoprotective efficacy and biosafety. PtRhIr/Ru SAN@M significantly improved BV2 cell viability in a dose‐dependent manner, with higher nanozyme concentrations correlating with greater cytoprotection (Figure 4D). In contrast, the Ru‐free counterpart (PtRhIr@M) enhanced cell viability only at a high concentration (48 µg/mL), underscoring the superior cytoprotective activity of PtRhIr/Ru SAN@M. Regarding toxicity, both nanozymes exhibited negligible cytotoxicity toward BV2 cells, even at 24 µg/mL (Figure 4E; Figure S15), highlighting their excellent biocompatibility. Calcein‐AM/Propidium Iodide (PI) dual staining further confirmed cell viability. CLSM images revealed a significantly higher proportion of live cells in the PtRhIr/Ru SAN@M‐treated group than in the PtRhIr@M‐treated and hemin‐induced ICH groups (Figure 4F,G; Figure S16). These results confirm the ability of PtRhIr/Ru SAN @M to reduce cell death, outperform PtRhIr@M, and suppress necrotic cell death pathways. Collectively, these findings demonstrate that PtRhIr/Ru SAN@M effectively protects BV2 cells from hemin‐induced injury in an in vitro ICH model by inhibiting cell death pathways.

### 2.4 Ferroptosis‐Inhibited Ability of RhIr/Ru SAN @M In Vitro

**FIGURE 5:**
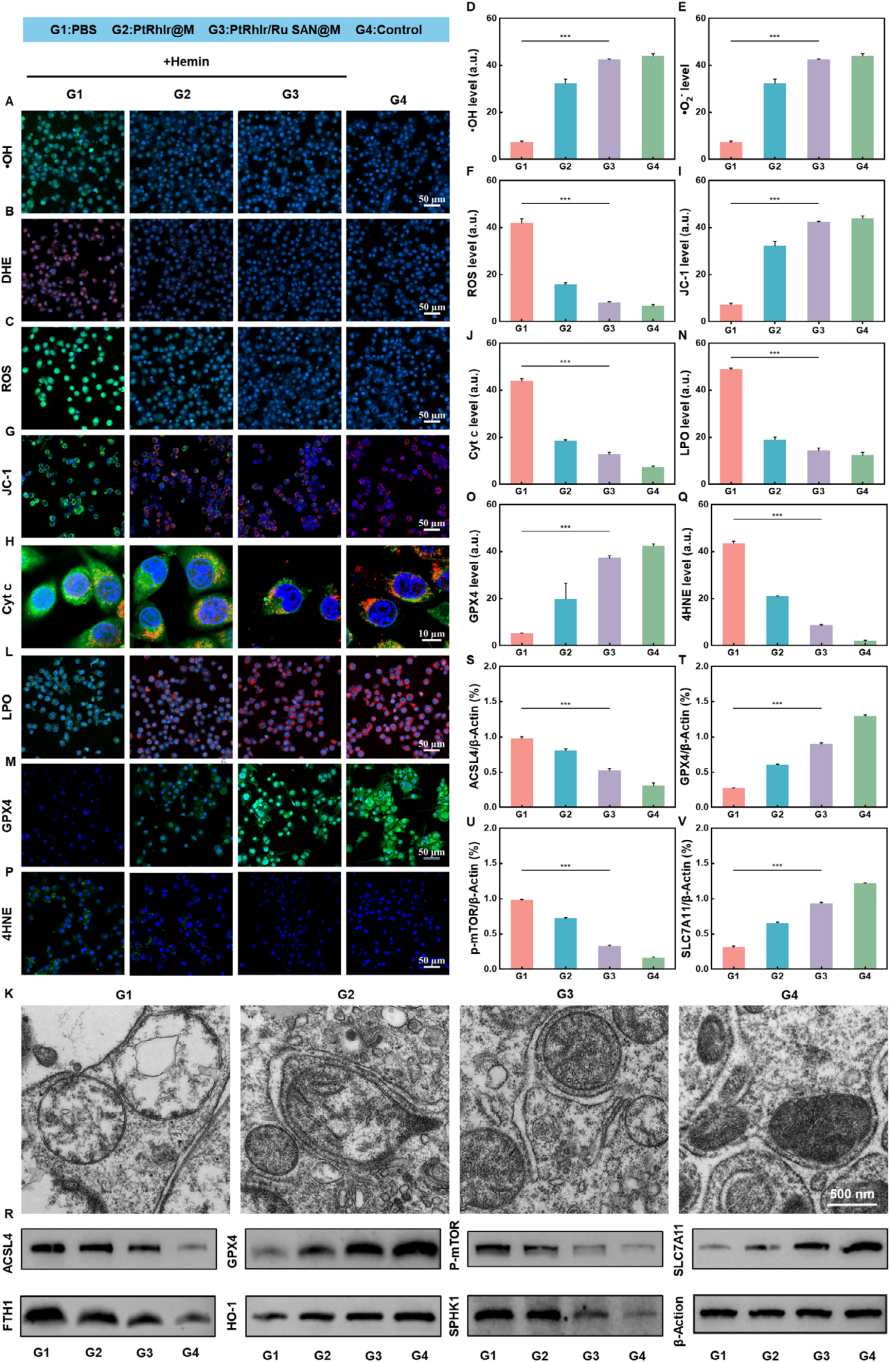
Ferroptosis‐inhibition ability of PtRhIr/Ru SAN@M in vitro. CLSM and bio‐TEM images, fluorescence intensity quantifications, and western blot analysis showing various staining and corresponding expression levels in BV2 cells with different treatments. (A) •OH staining and (B) corresponding quantification. (C) DHE staining and (D) quantification. (E) ROS staining and (F) quantification (n = 3, for each group). (G) jc‐1 and (H) cyt c staining, with corresponding fluorescence intensities of (I) jc‐1 and (J) cyt c. (K) Bio‐TEM images of mitochondrial morphology (n = 3, for each group). (L) LPO and (M) GPX4 staining, with corresponding fluorescence intensities of (N) LPO and (O) GPX4 (n = 3, for each group). (P) 4HNE staining and (Q) quantification (n = 3, for each group). (R) western blot analysis and (s–v) corresponding quantification of ACSL4, GPX4, p‐Mtor and SLC7A11 expression levels in BV2 cells with different treatments (n = 3, for each group). Statistical significance was assessed using a two tailed student's *t*‐test. Data are presented as means ± SD. ^*^ *p* < 0.05, ^**^ *p* < 0.01, ^***^ *p* < 0.001.

ROS generation is a pivotal driver of SBI following ICH, playing a crucial role in LPO, and ultimately triggering ferroptosis [8, 10, 49]. Building on our previous finding that PtRhIr/Ru SAN@M offers cytoprotection in this context, the current study further explores the underlying molecular mechanisms. Specifically, we emphasize the critical role of oxidative stress as a key factor in inflammation‐associated injury during ICH. To assess oxidative stress levels in each group, CLSM was performed using fluorescent probes specific for •OH (O27), •O_2_^−^ (DHE), and total ROS (DCFH) (Figure 5A–C; Figures S17–S19). In the hemin‐induced ICH group, intense green or red fluorescence signals indicated a marked increase in ROS, •OH, and •O_2_^−^ levels, highlighting the severe oxidative stress in this model. In contrast, fluorescence signals in the PtRhIr/Ru SAN@M‐treated group were substantially attenuated, closely resembling those in the control group. Quantitative analysis confirmed that levels of •OH, •O_2_^−^, and total ROS were significantly higher in the ICH group than in the PtRhIr@M, PtRhIr/Ru SAN@M, and control groups (Figure 5D–F). These findings demonstrate that PtRhIr/Ru SAN@M effectively mitigates oxidative stress in BV2 cells subjected to ICH, underscoring its potential as a potent antioxidant therapeutic agent in this context.

To further elucidate the protective mechanisms of PtRhIr/Ru SAN@M, we evaluated mitochondrial membrane potential (MMP) and cytochrome c (Cyt c) subcellular distribution using CLSM with the 5,5',6,6'‐tetrachloro‐1,1',3,3'‐tetraethyl‐imidacarbocyanine iodide (JC‐1) fluorescent probe and Cyt c immunofluorescence (Figure 5G,H; Figures S20 and S21). In the ICH group, JC‐1 predominantly displayed green fluorescence as a monomer, indicating mitochondrial depolarization, which is a hallmark of mitochondrial dysfunction [50, 51]. Cyt c fluorescence spread from the mitochondria to the cytoplasm, signifying mitochondrial membrane damage and Cyt c release, which are key events in the mitochondrial pathway of cell damage [52, 53]. In contrast, the PtRhIr/Ru SAN@M group exhibited a significantly restored red fluorescence from JC‐1, indicating the preservation of MMP. Moreover, Cyt c re‐accumulated in the mitochondrial region, with substantially reduced diffuse cytoplasmic fluorescence, closely resembling that of the control group. In the control group, JC‐1 appeared as red fluorescent aggregates, indicating a stable MMP, with Cyt c mainly localized in the mitochondria, signifying an intact mitochondrial structure and minimal Cyt c release into the cytoplasm. Quantitative analysis confirmed that JC‐1 aggregation and Cyt c mitochondrial localization were significantly reduced in the ICH group compared to the PtRhIr/Ru SAN@M and control groups (Figure 5I,J). These results indicate that PtRhIr/Ru SAN@M preserves MMP integrity and prevents Cyt c release, effectively interrupting the mitochondrial cell damage pathway. This multifaceted protective effect offers robust mitochondrial protection in BV2 cells in the ICH model. Additionally, mitochondrial damage was assessed using bio‐TEM. As shown in Figure 5K, PtRhIr/Ru SAN@M significantly alleviated mitochondrial shrinkage in ICH model cells, a hallmark of ferroptosis. These results suggest that the nanozyme protects mitochondrial integrity and maintains cellular energy metabolism. Collectively, our findings highlight the potential of PtRhIr/Ru SAN@M as a novel therapeutic agent for mitigating oxidative stress and preserving mitochondrial integrity in ICH, thus offering a promising strategy to reduce SBI.

LPO is a hallmark of ferroptosis, a regulated form of cell death characterized by iron‐dependent LPO [49]. Glutathione peroxidase 4 (GPX4) plays a crucial role in scavenging lipid peroxides, thereby preventing ferroptosis [51]. Together, LPO levels and GPX4 expression serve as key indicators of ferroptosis. To assess LPO accumulation and GPX4 expression in each group, we employed CLSM with BODIPY‐based LPO probes and GPX4 immunofluorescence (Figure 5L,M; Figures S22 and S23). In the ICH group, BODIPY probes displayed weak red and bright green fluorescence, indicating significant LPO accumulation following hemin induction. Corresponding GPX4 immunofluorescence signals were notably reduced, indicating suppressed GPX4 expression and loss of the capacity to clear lipid peroxides, thereby triggering ferroptosis. The malondialdehyde (MDA) detection results corroborate these findings, revealing significantly higher MDA levels in the ICH group than in the PtRhIr/Ru SAN@M‐treatment group (Figure S24). After PtRhIr/Ru SAN@M treatment, the red fluorescence from the BODIPY probes increased significantly, whereas the green fluorescence decreased. Quantitative analysis confirmed that LPO levels in the PtRhIr@M group were significantly lower than those in the ICH group (Figure 5N). Additionally, GPX4 immunofluorescence signals were significantly restored, approaching control group levels, with quantitative data showing higher GPX4 expression in the control group than in the ICH group (Figure 5O). Furthermore, CLSM imaging and quantitative analysis of 4‐hydroxynonenal (4‐HNE), an LPO product, further corroborated these findings; 4‐HNE levels were significantly higher in the ICH group than in the PtRhIr/Ru SAN@M and control groups, with PtRhIr/Ru SAN@M effectively reducing 4‐HNE accumulation (Figure 5P,Q; Figure S25). These results suggest that PtRhIr/Ru SAN@M not only directly inhibits LPO but also enhances lipid peroxide clearance by upregulating GPX4 expression. This dual mechanism, “reducing generation” and “enhancing clearance,” blocks ferroptosis and provides key mechanistic support for its protective effect against ICH damage. Based on the above experiments, we conclude that the rescue effect of PtRhIr/Ru SAN@M on BV‐2 cells in the ICH model may be associated with ferroptosis. To elucidate the mechanism by which PtRhIr/Ru SAN@M inhibits ferroptosis, we examined the expression of ferroptosis‐related proteins. We evaluated the expression levels of HO‐1, FTH1, SPHK1, p‐mTOR, SLC7A11, ACSL4, and GPX4 through Western blot analysis (Figure 5R–V; Figure S26) [50, 54], yielding consistent results. In BV‐2 cells from the ICH model, PtRhIr/Ru SAN@M treatment inhibited the expression of FTH1, SPHK1, p‐mTOR, and ACSL4 (promoters of ferroptosis) and upregulated HO‐1, SLC7A11, and GPX4 (inhibitors of ferroptosis). These findings indicate that PtRhIr/Ru SAN@M effectively modulates ferroptosis‐related protein expression, thereby suppressing ferroptosis. This comprehensive regulation of key ferroptosis pathways underscores the multifaceted protective mechanism of PtRhIr/Ru SAN@M in mitigating ICH‐induced cellular damage.

### 2.5 PtRhIr/Ru SAN@M Induces an Anti‐Inflammatory Response in Microglial

**FIGURE 6:**
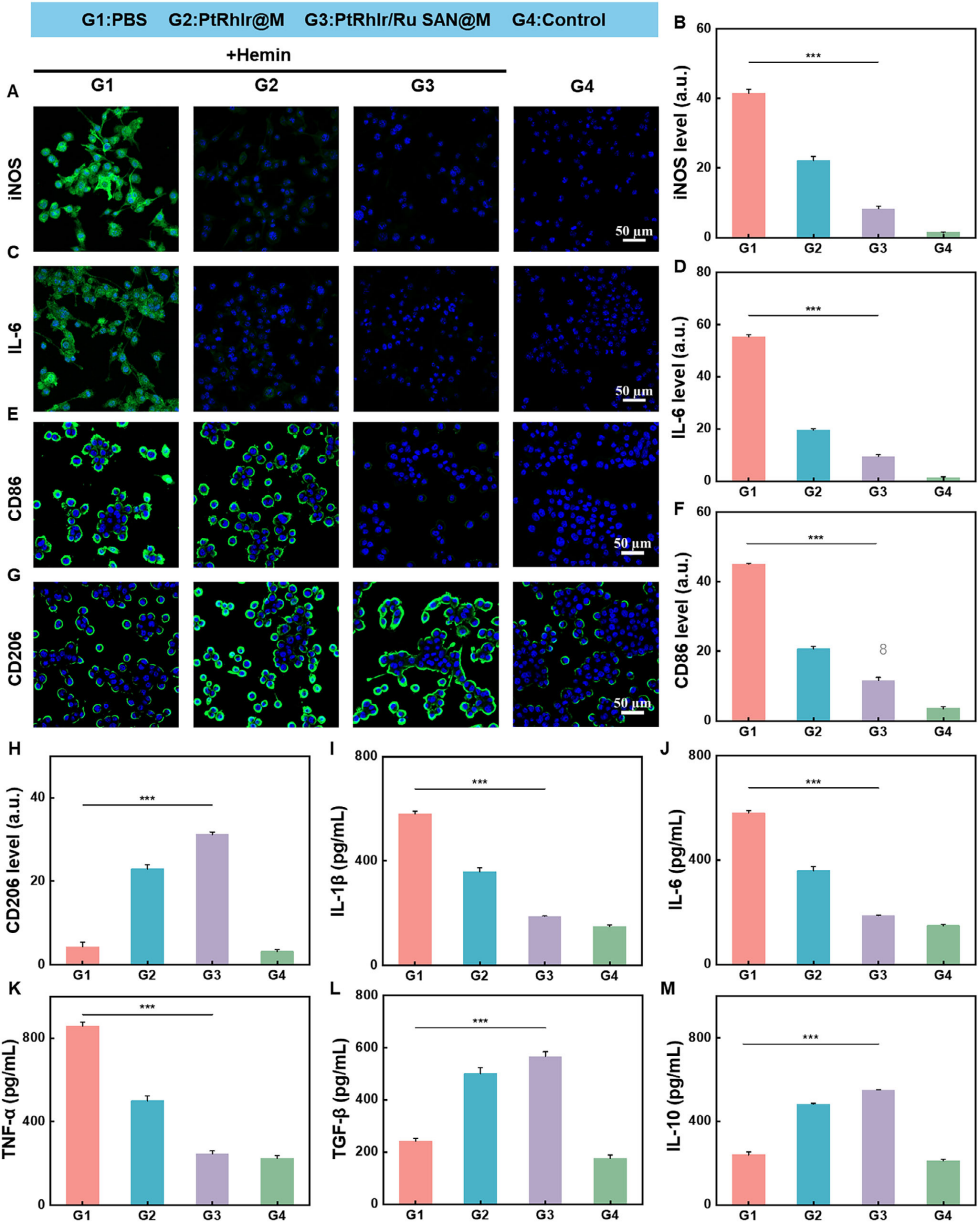
Regulation of BV2 cells phenotype and anti‐inflammatory effects by PtRhIr/Ru SAN@M. CLSM images and corresponding quantification of fluorescence intensity in BV2 cells with different treatments. (A) iNOS staining and (B) quantification. (C) IL‐6 staining and (D) quantification. (E) CD86 staining and (F) quantification. (G) cd206 staining and (H) quantification. (I–M) cytokine levels of IL‐1β, IL‐6, TNF‐α, TGF‐β, and IL‐6 in BV2 cells subjected to hemin after 24‐h treatments (n = 3, for each group). Statistical significance was assessed using a two tailed student's *t*‐test. Data are presented as means ± SD. ^*^ *p* < 0.05, ^**^ *p* < 0.01, ^***^ *p* < 0.001.

Microglia, the resident immune cells of the central nervous system, play a pivotal role in the pathogenesis of ICH. Pro‐inflammatory M_1_‐type microglia exacerbate neuronal damage and amplify the inflammatory hemorrhagic microenvironment by promoting oxidative stress and releasing pro‐inflammatory cytokines [55]. In contrast, anti‐inflammatory M_2_‐type microglia secrete anti‐inflammatory cytokines that alleviate the post‐stroke inflammatory environment and protect neurons [12]. Understanding microglial polarization is crucial for developing therapeutic strategies to mitigate the detrimental effects of ICH. To explore the effect of PtRhIr/Ru SAN@M on microglial polarization, we treated BV2 cells with an in vitro ICH model to mimic stroke injury. CLSM imaging revealed significant changes in microglial phenotypes. The M_2_ marker, CD206, was markedly upregulated in the PtRhIr/Ru SAN@M group, indicating a shift toward an anti‐inflammatory M_2_ phenotype. Meanwhile, the fluorescence signals of the pro‐inflammatory markers inducible nitric oxide synthase (iNOS) and interleukin‐6 (IL‐6) were significantly reduced (Figure 6A–D; Figures S27 and S28). In contrast, in the ICH group, the M_1_ marker CD86 exhibited a significant increase, highlighting the pro‐inflammatory M_1_ phenotype (Figure 6E,F; Figure S29). These results indicate that PtRhIr/Ru SAN@M can effectively reprogram microglia from the M_1_ to the M_2_ phenotype, thereby inhibiting the production and release of pro‐inflammatory molecules (Figure 6G,H; Figure S30). To further validate these findings, we conducted an enzyme‐linked immunosorbent assay to quantify cytokine secretion. Enzyme‐linked immunosorbent assays confirmed that PtRhIr/Ru SAN@M treatment significantly reduced the secretion of pro‐inflammatory cytokines, including tumor necrosis factor‐alpha (TNF‐α), IL‐1β, and IL‐6 (Figure 6I–K). Simultaneously, the treatment significantly enhanced the secretion of anti‐inflammatory cytokines, such as TGF‐β and IL‐10 (Figure 6L,M). These findings underscore the ability of PtRhIr/Ru SAN@M to modulate microglial polarization and cytokine secretion, thereby improving the inflammatory microenvironment post‐ICH and exerting robust protective and anti‐inflammatory effects. In summary, our study demonstrated that PtRhIr/Ru SAN@M mitigates oxidative stress and ferroptosis by modulating microglial polarization, shifting the balance from a pro‐inflammatory M_1_ phenotype to an anti‐inflammatory M_2_ phenotype. This multifaceted mechanism of action suggests that PtRhIr/Ru SAN@M is a promising therapeutic agent for mitigating the detrimental effects of ICH and promoting neuroprotection.

### 2.6 Biocompatibility and Cellular Uptake of PtRhIr/Ru SAN@M in Neurons

A series of meticulously designed experiments was conducted to comprehensively assess the biocompatibility and cellular uptake efficiency of PtRhIr/Ru SAN@M in HT22 neuronal cells. CLSM analysis of the cellular uptake revealed a time‐dependent increase in the red fluorescence signal of PtRhIr/Ru SAN@M in HT22 cells (Figure S31). This time‐dependent increase in the fluorescence intensity clearly indicates the efficient internalization of PtRhIr/Ru SAN@M, driven by its strong cellular affinity. Furthermore, colocalization experiments confirmed that PtRhIr/Ru SAN@M escaped lysosomal sequestration in HT22 cells (Figure S32). This localization clarified its site of action, suggesting that PtRhIr/Ru SAN@M was effectively processed and utilized within cellular compartments. In summary, our comprehensive evaluation of PtRhIr/Ru SAN@M in HT22 neuronal cells demonstrates its excellent biocompatibility and cellular uptake, highlighting its potential as a safe and effective therapeutic agent. Calcein‐AM/PI dual staining was performed to evaluate the cytotoxicity and viability of HT22 cells treated with PtRhIr/Ru SAN@M. This staining approach enabled the concurrent discrimination of live and dead cells, yielding a clear and quantitative evaluation of cell viability. The results showed a significantly higher proportion of live HT22 cells in the PtRhIr/Ru SAN@M‐treated group compared to the ICH and PtRhIr@M‐treated groups (Figure S33). CCK‐8 assays were conducted to assess cell viability, cytotoxicity, and the protective effects of PtRhIr/Ru SAN@M in an in vitro ICH model. The results demonstrated that PtRhIr/Ru SAN@M significantly enhanced cell viability compared to PtRhIr@M (Figure S34A). Both compounds showed minimal cytotoxicity toward HT22 cells (Figure S34B,C), which is consistent with their low toxicity profiles in BV2 cells. This excellent biocompatibility confirms the safety of PtRhIr/Ru SAN@M in vivo and highlights its potential for clinical translation. These findings indicate that PtRhIr/Ru SAN@M offers dual benefits: promotion of cell survival and reduction of toxic damage, particularly by targeting and alleviating necrotic pathways, which are crucial features of neuroprotective agents.

### 2.7 Protective Effects of PtRhIr/Ru SAN@M in HT22 Cells

**FIGURE 7:**
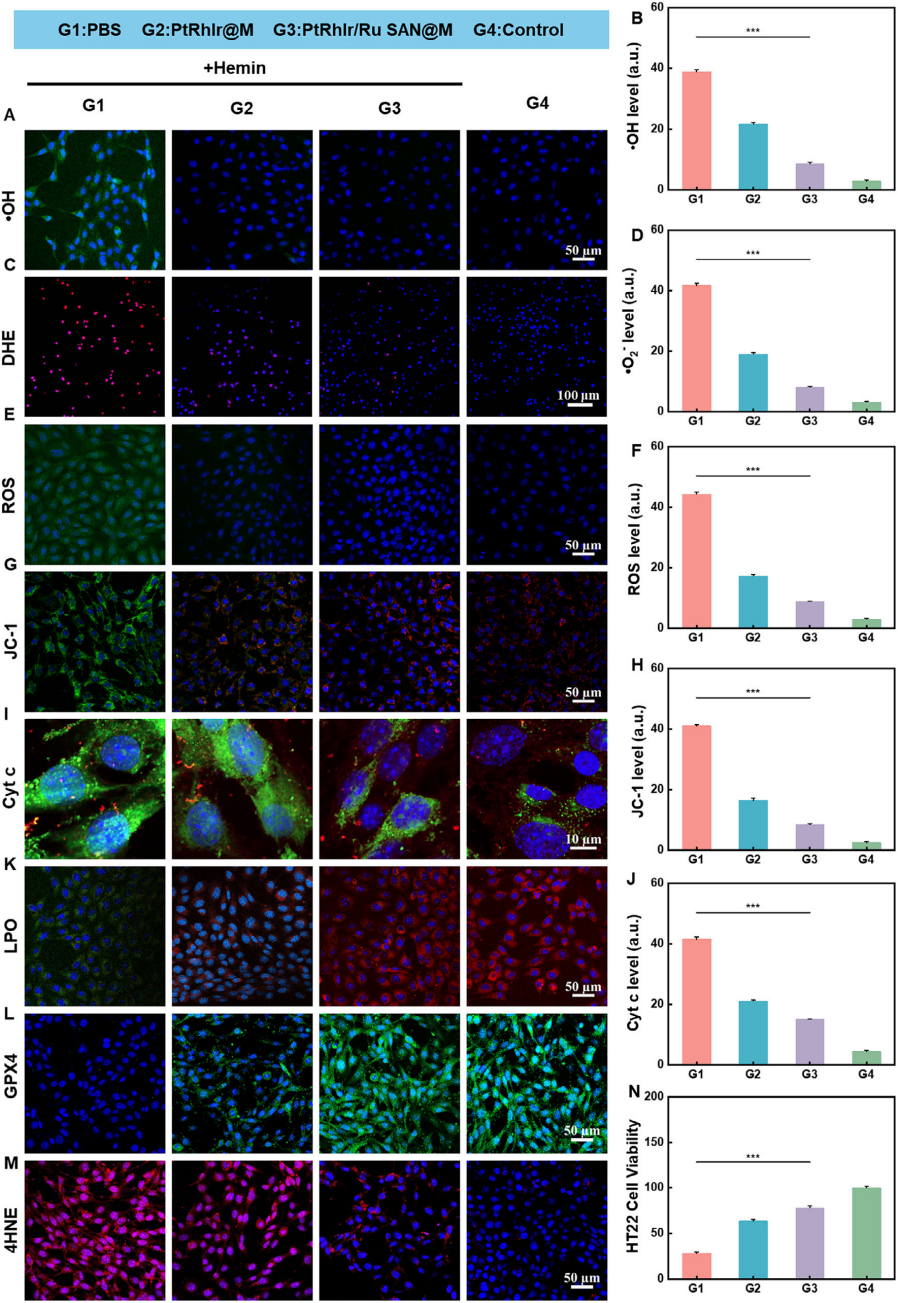
Neuroprotective effects in vitro by ptrhir/ru san@m. clsm images and corresponding fluorescence intensity quantifications in HT22 cells with different treatments. (A) •oh staining and (B) quantification (n = 3, for each group). (C) DHE staining and (D) quantification (n = 3, for each group). (E) ROS staining and (F) quantification (n = 3, for each group). (G) JC‐1 staining and (H) quantification (n = 3, for each group). (I) cyt c staining and (J) quantification (n = 3, for each group). (K) LPO, (L) GPX4, and (M) 4HNE staining in HT22 cells. (N) neuroprotective effects and modulation of microglia‐driven inflammation by PtRhIr/Ru SAN@M in HT22 cells (n = 3, for each group). Statistical significance was assessed using a two tailed student's *t*‐test. Data are presented as means ± SD. ^*^ *p* < 0.05, ** *p* < 0.01, *** *p* < 0.001.

Given that microglia‐driven inflammation and oxidative stress are central to neuronal injury [10,12,54], we next evaluated the neuroprotective effects of PtRhIr/Ru SAN@M in HT22 cells. Our study aimed to elucidate the multifaceted mechanisms by which PtRhIr/Ru SAN@M mitigates neuronal injury in the context of ICH. Levels of •OH, •O_2_^−^, and general ROS were significantly higher in the ICH group than in the control group. Although PtRhIr@M partially reduced these levels, PtRhIr/Ru SAN@M achieved the most pronounced decrease, nearly restoring them to control values (Figure 7A–F; Figures S35–S37). This finding underscores the superior antioxidant capacity of PtRhIr/Ru SAN@M in mitigating oxidative stress, which is a major contributor to neuronal injury. Mitochondrial dysfunction, as reflected by JC‐1 depolarization and Cyt c release, was most pronounced in the ICH group. PtRhIr@M partially mitigated this dysfunction, whereas PtRhIr/Ru SAN@M produced a marked restoration (Figure 7G–J; Figures S38–S39). The JC‐1 assay showed that PtRhIr/Ru SAN@M effectively preserved MMP, whereas Cyt c immunofluorescence revealed reduced cytoplasmic Cyt c release, indicating that mitochondrial integrity was maintained. The preservation of mitochondrial function is crucial for maintaining cellular energy metabolism and preventing apoptosis. The LPO levels were significantly higher in the ICH group than in the control group. PtRhIr/Ru SAN@M exhibited the most pronounced reduction, nearly restoring the levels to those of the control (Figure 7K; Figure S40). MDA levels were markedly elevated in the ICH group, indicating extensive LPO. PtRhIr/Ru SAN@M effectively inhibited MDA accumulation, confirming its potent suppression of LPO (Figure S41). GPX4 expression was markedly reduced in the ICH group, whereas PtRhIr/Ru SAN@M restored GPX4 levels more effectively than PtRhIr@M (Figure 7L; Figure S42). The upregulation of GPX4 is crucial for enhancing the antioxidant capacity of neurons, thereby mitigating oxidative stress and preventing ferroptosis. 4‐HNE levels, a terminal product of LPO, were highest in the ICH group. PtRhIr/Ru SAN@M markedly reduced 4‐HNE accumulation, further confirming its potent suppression of LPO (Figure 7M; Figure S43). This reduction in 4HNE levels indicated a significant decrease in LPO and oxidative damage. Microglial‐driven inflammation and oxidative stress are crucial contributors to neuronal injury. To assess their effect on neuronal viability, the culture medium from the four BV2 groups was transferred to HT22 cells. CCK‐8 assays showed a marked reduction in HT22 cell viability in the ICH group, whereas PtRhIr/Ru SAN@M treatment significantly restored viability (Figure 7N). This indicates that PtRhIr/Ru SAN@M effectively mitigated microglia‐driven neurotoxicity and safeguarded neuronal survival. In summary, our findings indicate that PtRhIr/Ru SAN@M not only reshapes microglial inflammatory phenotypes to improve the brain microenvironment but also directly protects neurons. It counteracts oxidative stress, preserves mitochondrial function, and suppresses LPO. At the same time, it enhances antioxidant capacity through GPX4 upregulation. Consequently, neuronal damage is markedly alleviated in the ICH model.

### 2.8 Study on Molecular Mechanisms

#### 2.8.1 Differential Analysis and Visualization

A variance‐stabilizing transformation from DESeq2 was applied to normalize the RNA‐seq data. A heatmap was generated based on the top 50 differentially expressed genes (DEGs), comprising 25 upregulated and 25 downregulated transcripts. Subsequently, the genes were classified as upregulated, downregulated, or non‐significant based on the log_2_ fold change and adjusted p‐values, with each category visualized using distinct colors. Differences in gene expression and statistical significance were visualized using a volcano plot.

#### 2.8.2 Pathway Enrichment Analysis

Gene Ontology (GO) enrichment analysis of the significantly DEGs was performed using the clusterProfiler package, covering three major categories: biological processes, cellular components, and molecular functions. Significant pathways were determined based on p‐(< 0.05) and q‐values (< 0.2). The Kyoto Encyclopedia of Genes and Genomes (KEGG) pathway enrichment analysis was conducted using the enrichKEGG function with the same significance thresholds of p‐values (< 0.05) and q‐values (< 0.2). The enrichment results were visualized using bar plots. Additionally, the gene‐pathway network relationships for both GO and KEGG enrichment analyses were visualized. A ranked gene list was generated from the DESeq2 results, and Gene Set Enrichment Analysis was performed using hallmark gene sets. A p‐value threshold (< 0.05) was applied, and significantly enriched pathways were visualized.

#### 2.8.3 Differential Gene Expression Analysis

First, the Fragments Per Kilobase of transcript per Million mapped reads data were converted to Transcripts Per Million for normalization to ensure the comparability of gene expression. The Transcripts Per Million data for the target genes were extracted and merged with the sample information table, providing a foundation for subsequent analysis. Next, a t‐test was performed for each target gene to calculate significant differences between the experimental and control groups. Finally, ggplot2 was used to create violin plots to visualize the differences in gene expression.

#### 2.8.4 Bioinformatics Analysis

**FIGURE 8:**
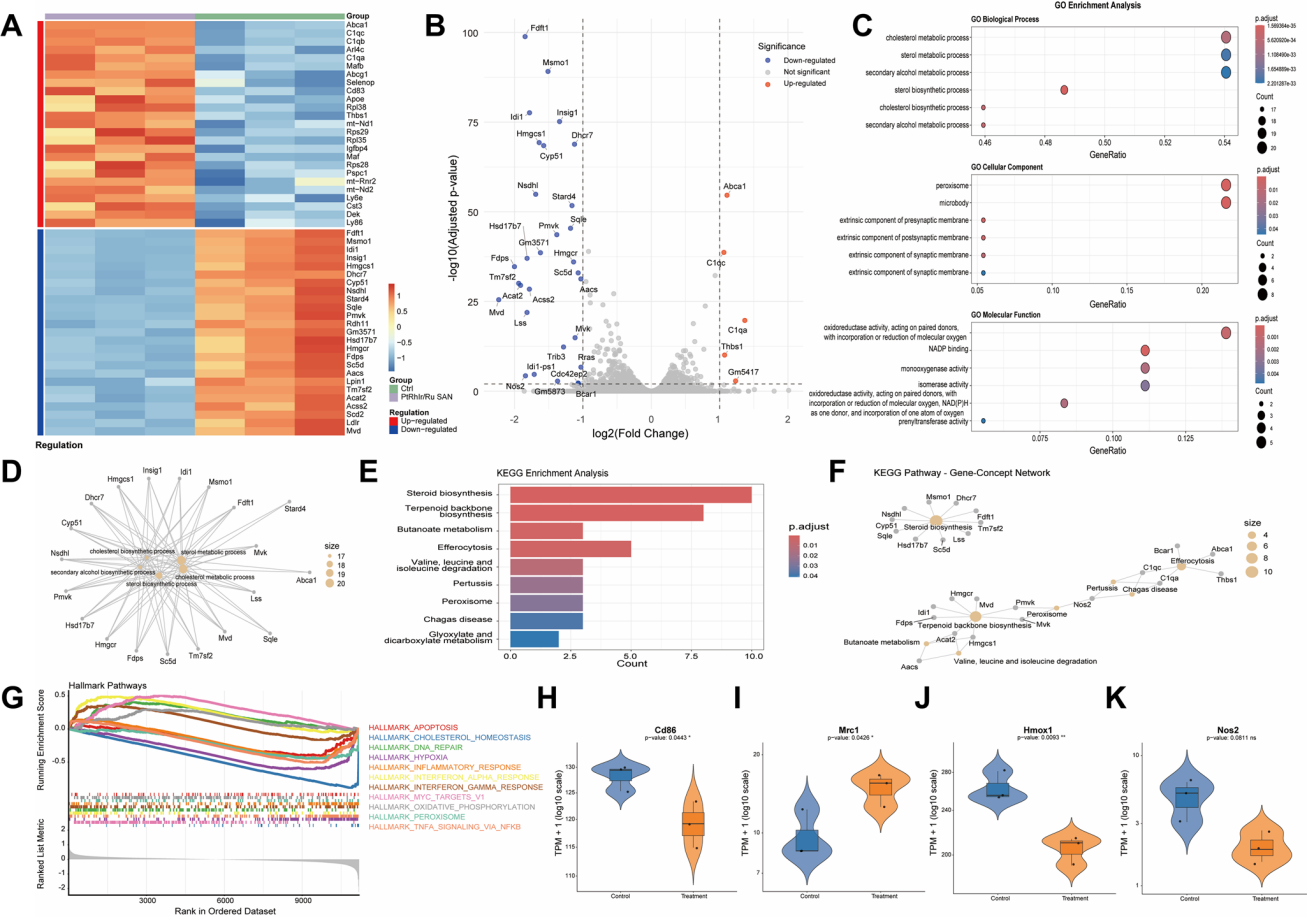
Gene expression and pathway analysis. (A) heatmap displaying gene expression levels between the PtRhIr/Ru SAN@M and control groups. (B) volcano plot illustrating gene expression differences between the PtRhIr/Ru SAN@M and control groups, with significantly upregulated and downregulated genes highlighted by red and blue dots, respectively. (C) Gene ontology enrichment analysis showing the biological processes, cellular components, and molecular functions significantly associated with the differentially expressed genes between the PtRhIr/Ru SAN@M and control groups. (D) gene‐concept network depicting the relationships between key genes and their involvement in significant biological pathways. (E) KEGG enrichment analysis highlighting the most significantly enriched pathways associated with the differentially expressed genes between the PtRhIr/Ru SAN@M and control groups. (F) KEGG pathway gene‐concept network illustrating the connections between enriched pathways and the corresponding differentially expressed genes. (G) pathway enrichment analysis based on hallmark gene sets from the MSIGDB database, comparing the ptrhir/ru san@m and control groups. (H‐L) violin plots showing the distribution of expression levels for the genes cd86, mrc1, hmox1, and nos2, highlighting the differences in expression between the ptrhir/ru san@m and control groups. data are presented as means ± SD (n ≥ 3).

After analyzing the DEGs between the control and PtRhIr/Ru SAN@M groups, we observed significant alterations in gene expression levels and pathway enrichment. The gene expression heatmap displayed clear differences in expression patterns, which were further confirmed using volcano plots (Figure 8A,B). Complement family genes (C1qa, C1qb, and C1qc) were significantly upregulated in the PtRhIr/Ru SAN@M group. The complement system is a core pathway involved in neuroinflammation, suggesting that nanomedicine interventions may modulate the complement pathway to influence the inflammatory response. Mitochondrial functional genes (mt‐Nd1, mt‐Rnr2, and mt‐Nd2) were also upregulated in the PtRhIr/Ru SAN@M group. Mitochondria are central to oxidative stress, and changes in these mitochondrial genes reflect remodeling of mitochondrial function, possibly participating in the compensatory regulation of oxidative stress. GO enrichment analysis revealed that PtRhIr/Ru SAN@M treatment significantly affected metabolic processes, biosynthetic processes, and antioxidant activity, suggesting that PtRhIr/Ru SAN@M treatment may alleviate oxidative stress and inflammation by regulating these biological processes (Figure 8C,D). KEGG pathway analysis further indicated the enrichment of key pathways, such as steroid biosynthesis, butanoate metabolism, and peroxisomal activity, supporting the role of PtRhIr/Ru SAN@M treatment in modulating oxidative stress and inflammation (Figure 8E,F). Additionally, Hallmark pathway analysis highlighted key pathways related to apoptosis, cholesterol homeostasis, and the immune response (Figure 8G). Violin plots showed higher expression of the pro‐inflammatory phenotype marker Cd86 in the control group, whereas the PtRhIr/Ru SAN@M group exhibited higher expression of the anti‐inflammatory phenotype marker Mrc1 (Figure 8H,I). Furthermore, changes in the expression of HMOX1 and NOS2 further validated the effects of PtRhIr/Ru SAN@M treatment on the regulation of inflammation and oxidative stress (Figure 8J,K). These results suggest that PtRhIr/Ru SAN@M treatment may exert neuroprotective effects by modulating multiple molecular pathways associated with oxidative stress and inflammation.

### 2.9 Targeting, Biosafety, and Biodistribution of PtRhIr/Ru SAN@M in ICH

**FIGURE 9:**
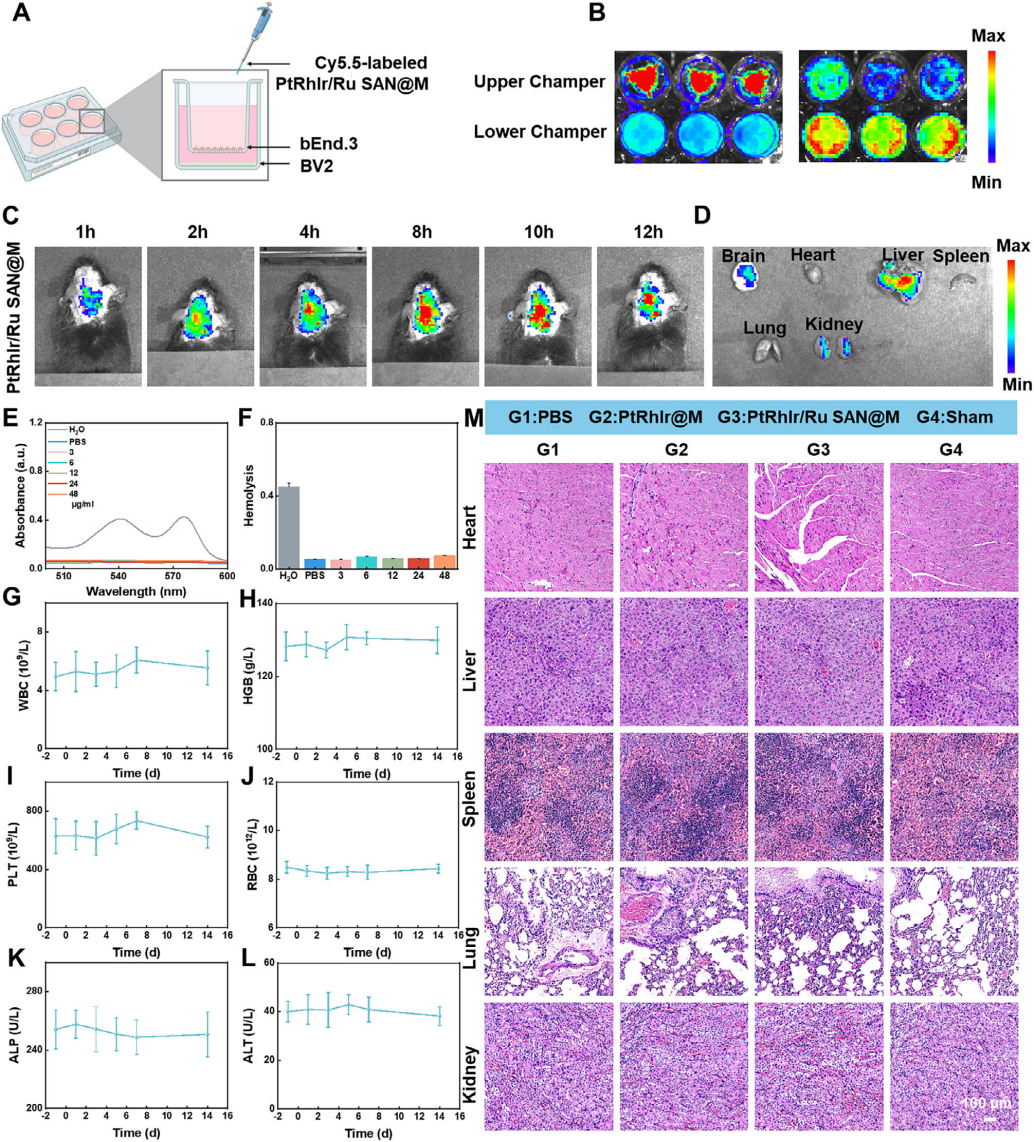
Targeting ability and biodistribution of ptrhir/ru san@m in vivo. (A) schematic of the in vitro bbb model construction. (B) fluorescence images of the upper and lower chambers after treatment with cy5.5‐labeled PtRhIr/Ru SAN@M. (C) In vivo fluorescence images of ICH model mice after intravenous injection of cy5.5‐labeled PtRhIr/Ru SAN@. (D) fluorescence images of main organs (brain, heart, liver, spleen, lung, kidney) 12 h post‐injection. (E,f) hemolysis test (n = 3, for each group). (G–j) hematological analysis of blood samples from each group. (K,l) serum biochemical analysis of blood samples from each group (n = 3, for each group). (M) h&e staining of brain tissue from each group. Data are presented as means ± SD.

To examine the BBB‐penetrating and BBB‐targeting properties of PtRhIr/Ru SAN@M in ICH, an in vitro BBB model was constructed using a Transwell setup. BEnd.3 cells were cultured in the upper chamber to mimic the BBB, whereas the BV2 cells in the lower chamber served as receptor cells (Figure 9A) [56,57]. Fluorescence imaging of Cy5.5‐labeled PtRhIr/Ru SAN@M in BV2 cells was performed at 6 h (left) and 24 h (right) (Figure 9B). At 6 h, fluorescence signals were predominantly observed in the upper chamber with minimal signals in the lower chamber. After 24 h, the fluorescence intensity substantially increased in the lower chamber and eventually exceeded that in the upper chamber. These results confirmed that PtRhIr/Ru SAN@M successfully crossed the BBB and was efficiently internalized by BV2 cells, indicating its potential for targeted delivery.

To further investigate in vivo targeting and biodistribution, Cy5.5‐labeled PtRhIr/Ru SAN@M was intravenously injected into ICH model mice. In vivo imaging (Figure 9C) showed that fluorescence signals in the brain increased progressively at various time points (1, 2, 4, and 8). Fluorescence intensity peaked at 8 h and gradually declined thereafter. These findings suggest the efficient BBB penetration and ICH site accumulation of PtRhIr/Ru SAN@M, with fluorescence initially increasing and clearing over time. This pattern indicates targeted delivery to ICH‐related inflammatory regions, likely driven by inflammation‐induced chemotaxis. At 12 h, the fluorescence imaging of the heart, liver, spleen, lungs, kidneys, and brain showed the highest signal intensity in the ICH region, with notable accumulation in the liver and kidneys (Figure 9D). These results highlight the efficient distribution of PtRhIr/Ru SAN@M in ICH.

Biosafety was evaluated through hemolysis and hematological, biochemical, and histological analyses. Hemolysis assays showed that even at 48 µg/mL, the hemolysis rate of PtRhIr/Ru SAN@M remained below 5%, indicating minimal erythrocyte disruption (Figure 8E,F). Hematological analysis of the PtRhIr/Ru SAN@M group revealed no significant abnormalities, suggesting a minimal impact on the systemic blood parameters (Figure 8G–J; Figure S44). Serum biochemical markers, including alkaline phosphatase, alanine aminotransferase, aspartate aminotransferase, blood urea nitrogen, and creatinine, remained within normal ranges (Figure 8K,L; Figure S45), indicating no significant hepatic or renal toxicity. Hematoxylin and eosin (H&E) staining of the major organs (heart, liver, spleen, lungs, and kidneys) revealed no observable pathological damage (Figure 9M). These findings confirmed the excellent biocompatibility and biosafety of PtRhIr/Ru SAN@M. Nanoparticles are metabolized and cleared primarily by the liver and kidneys, minimizing systemic accumulation and toxicity.

In conclusion, PtRhIr/Ru SAN@M exhibits efficient BBB penetration in vitro and significantly targets the ICH region in vivo. Its exceptional biocompatibility and biosafety make it a promising candidate for targeted ICH therapy. The combination of BBB crossing, ICH‐region targeting, and low systemic toxicity highlights PtRhIr/Ru SAN@M as a versatile therapeutic platform for ICH.

### 2.10 In Vivo Therapeutic Efficacy of PtRhIr/Ru SAN@M in ICH Model

**FIGURE 10:**
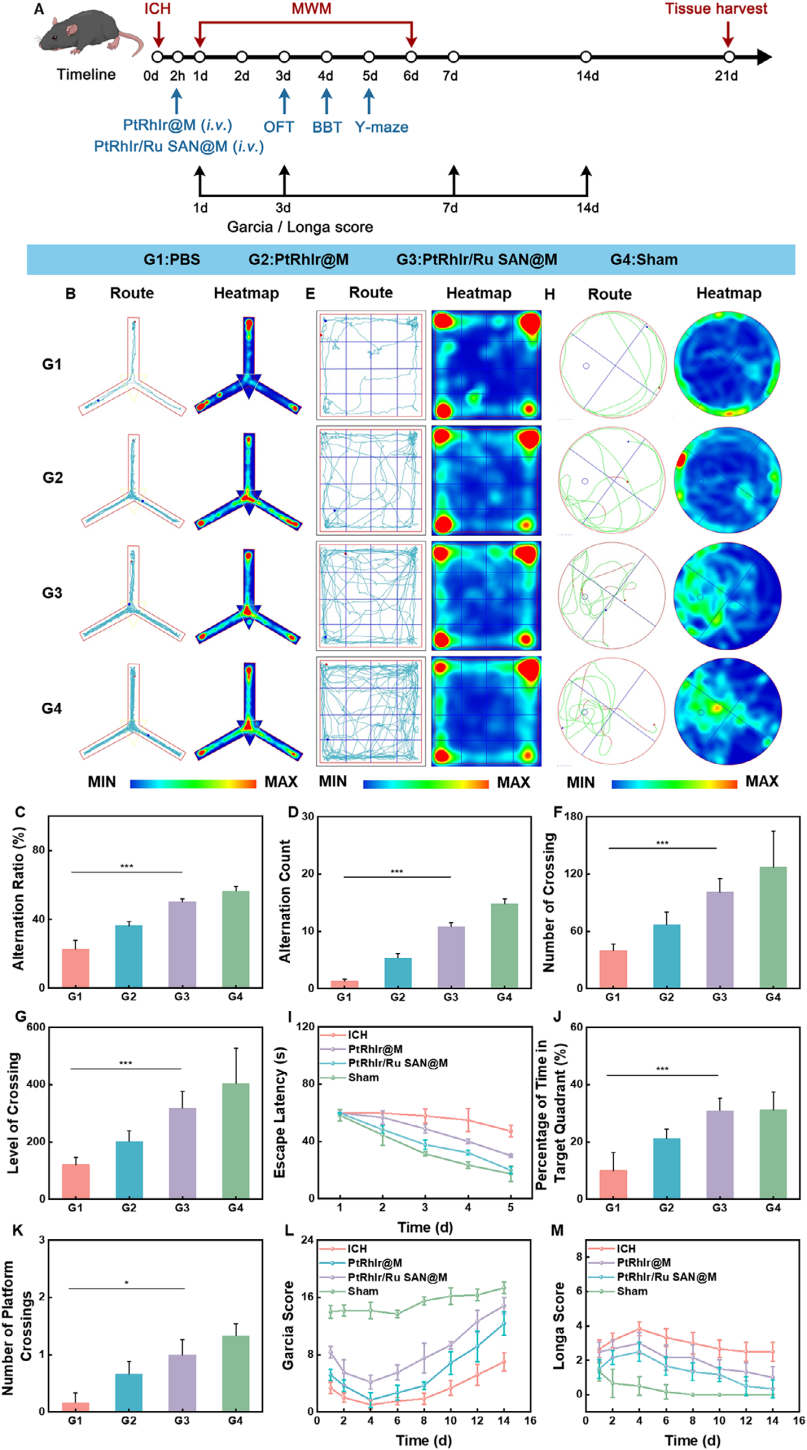
Therapeutic effect of PtRhIr/Ru SAN@M in vivo. (A) schematic of the experimental protocol for ich treatment with PtRhIr/Ru SAN@M. (B) Representative YMT trajectories and heat maps for rats in four groups. Quantification of YMT: c) alternation ratio and d) alternation count (n = 6, for each group). E) representative of trajectories and heat maps. Quantification of OFT: F) number of crossings and G) level of crossing (n = 6, for each group). H) representative mwm trajectories and heat maps. Quantification of mwm: (I) escape latency, (J) time in target quadrant, and (K) platform crossing frequency (n = 6, for each group). (L) Modified Garcia score and (M) Longa score after different treatments (n = 6, for each group). Statistical significance was assessed using a two tailed student's *t*‐test. Data are presented as means ± SD. *p < 0.05, **p < 0.01, ***p < 0.001.

The in vivo pharmacodynamics protocol is shown in Figure 10A. PtRhIr/Ru SAN@M was intravenously administered 2 h after ICH induction. Neurological assessments were conducted from days 1 to 7 post‐ICH, and brain tissue was collected on day 21 for further analysis. Behavioral tests validated the neuroprotective effects of PtRhIr/Ru SAN@M on cognition and motor function. In the Y‐maze test (Figure 10B), mice with ICH displayed disordered exploration with dispersed activity hotspots, indicative of impaired spatial working memory [58]. The alternation ratio and alternative count were significantly lower than those of the PtRhIr/Ru SAN@M and sham groups. Conversely, mice treated with PtRhIr/Ru SAN@M showed orderly exploration patterns and focused activity hotspots, along with markedly improved scores, suggesting a substantial recovery of spatial working memory (Figure 9C,D) [58]. In the open field test (OFT) (Figure 10E), ICH mice exhibited short peripheral movement trajectories, with the number of crossings and level of crossing significantly lower than those in the PtRhIr/Ru SAN@M and sham groups. Mice treated with PtRhIr/Ru SAN@M exhibited extended, widely distributed movement patterns and elevated vertical activity, reflecting improved motor coordination and spontaneous behavior (Figure 9F–G) [56, 57, 58]. In the Morris water maze (MWM) (Figure 10H), ICH mice displayed prolonged escape latencies (Figure 10I). After platform removal, they spent less time in the target quadrant and crossed the platform fewer times (Figure 9J,K) [50, 59]. PtRhIr/Ru SAN@M‐treated mice showed more directed exploration of the target quadrant, with distinct activity hotspots, approaching those of the sham group and confirming restoration of spatial learning and memory. In the balance beam test (Figure S46), ICH mice took longer to cross, reflecting impaired motor coordination. PtRhIr/Ru SAN@M‐treated mice crossed the beam more quickly, showing improved motor coordination similar to the sham group performance. Weight monitoring from days 1 to 14 post‐ICH revealed significant weight loss in the ICH group, indicating pronounced physiological stress. In contrast, the PtRhIr/Ru SAN@M group demonstrated a slower rate of weight loss, approaching the sham group levels (Figure S47). This suggests that the PtRhIr/Ru SAN@M treatment mitigates the physiological stress caused by ICH and aids in restoring stability. Building on the in vitro evidence that PtRhIr/Ru SAN@M modulates microglial inflammation and protects neurons, we further examined its effects on neurological recovery in an ICH in vivo model. The neurological function scores (Figure 9L,M) showed persistently low Garcia scores and elevated Longa scores in the ICH group, indicating severe impairment. Mice treated with PtRhIr/Ru SAN@M exhibited increased Garcia scores and markedly reduced Longa scores, indicating substantial recovery from ICH‐induced neurological dysfunction. In summary, PtRhIr/Ru SAN@M demonstrates strong therapeutic potential for ICH, mitigating physiological decline and promoting neurological recovery across the motor and cognitive domains. This multifaceted approach offers a promising strategy for clinical recovery after ICH.

### 2.11 Anti‐Inflammatory and Neuroprotective Mechanisms of PtRhIr/Ru SAN@M In Vivo

**FIGURE 11:**
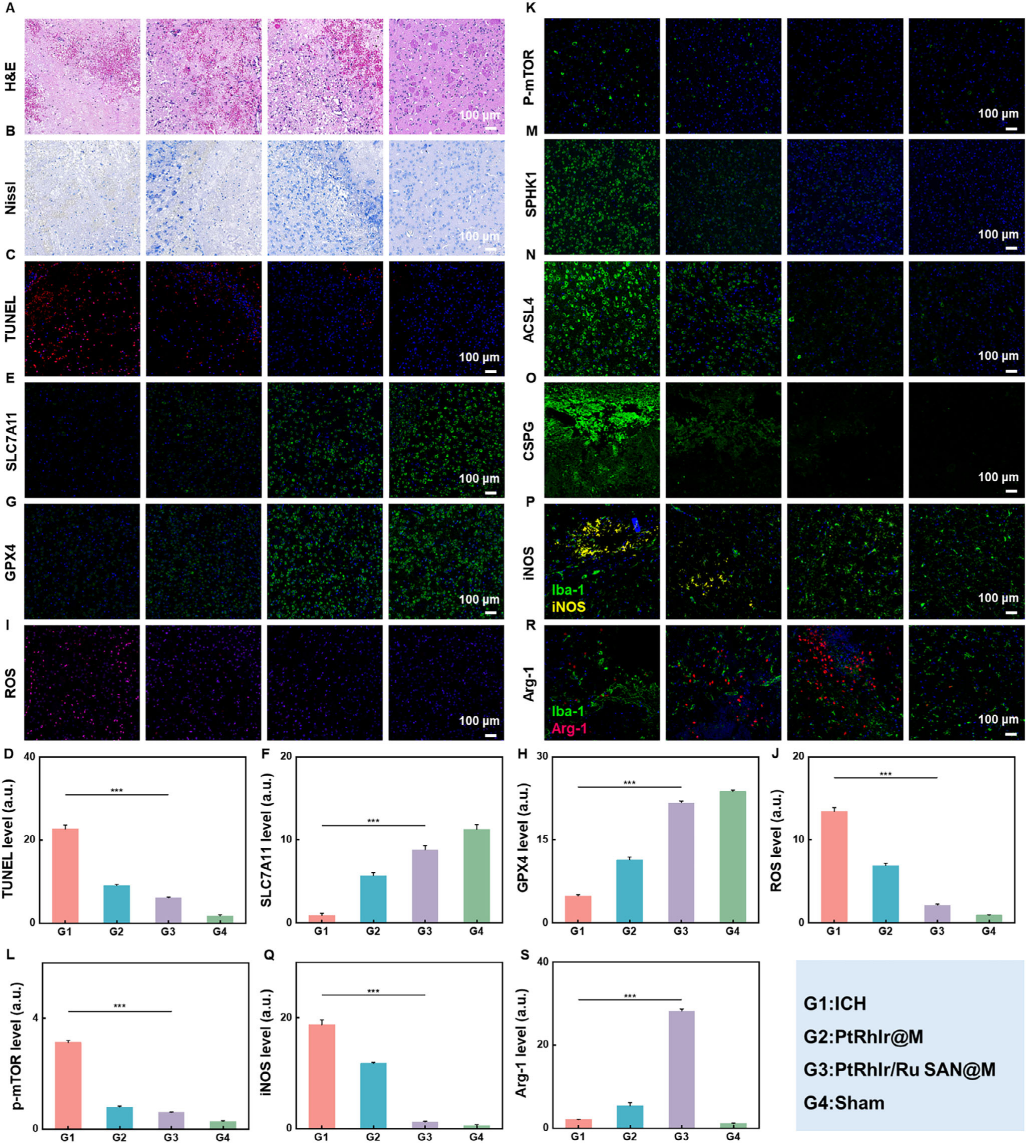
Therapeutic effects of PtRhIr/Ru SAN@M on ich in vivo. (A) H&E and (B) Nissl staining of brain slices from each group in the ich lesion area. (C) Tunel staining with (D) corresponding fluorescence intensity quantification (n = 3, for each group). (E) SLC7A11 staining and (F) quantification (n = 3, for each group). (G) GPX4 staining and (H) quantification (n = 3, for each group). (I) ROS staining and (J) quantification (n = 3, for each group). (K) p‐MTOR staining and (L) quantification (n = 3, for each group). (M) SPHK1 and (N) ACSL4 staining of brain slices in the ICH lesion area. (O) Representative images of scar‐forming astrocytes detected by GFAP staining. (P) Co‐immunofluorescence staining of IBA‐1, INOS, and DAPI in the ich lesion area with (Q) corresponding fluorescence intensity quantification (n = 3, for each group). (R) Co‐immunofluorescence staining of IBA‐1, ARG‐1, and DAPI in the ICH lesion area with (s) corresponding fluorescence intensity quantification (n = 3, for each group). Statistical significance was assessed using a two tailed student's t‐test. Data are presented as means ± SD. ^*^ *p* < 0.05, ^**^ *p* < 0.01, ^***^ *p* < 0.001.

Having shown that PtRhIr/Ru SAN@M drives M_2_ polarization and modulates inflammatory cytokines in vitro, we assessed its potential to elicit similar anti‐inflammatory and neuroprotective effects in vivo. First, we assessed brain tissue repair by H&E staining. In the collagenase‐induced ICH model, PtRhIr@M produced only a modest reduction in injury, whereas PtRhIr/Ru SAN@M markedly decreased both lesion area and hematoma volume relative to the ICH and PtRhIr@M groups (Figure 11A). Moreover, mice treated with PtRhIr/Ru SAN@M exhibited reduced inflammatory infiltration, suggesting that the nanoplatform promoted brain repair by suppressing inflammation. Nissl staining was performed to assess neuronal damage around the hematoma (Figure 11B). In the ICH group, neurons displayed significant deformation, shrinkage, and marked loss of NISSL bodies. PtRhIr@M treatment produced noticeable improvements in neuronal morphology. However, the PtRhIr/Ru SAN@M group showed the most pronounced neuroprotection, with most neurons maintaining a healthy morphology and an increased number of Nissl bodies, suggesting superior neuroprotection. TUNEL staining was used to evaluate cell death. The ICH group exhibited the highest level of cell death, particularly in the injured areas. PtRhIr/Ru SAN@M treatment markedly reduced cell death compared to the PtRhIr@M group (Figure 10C,D). Given the link between microglial phenotype transformation and ferroptosis, we examined the brain tissue from ICH mice using immunofluorescence for ferroptosis markers. The ICH group displayed reduced expression of SLC7A11 and GPX4, indicating compromised antioxidant defense and impaired cysteine transport (Figure 10E–H). Elevated levels of ROS, p‐mTOR, SPHK1, and ACSL4, all key markers of ferroptosis, were also observed. PtRhIr/Ru SAN@M treatment restored GPX4 expression and markedly reduced ROS, p‐mTOR, SPHK1, and ACSL4 levels, bringing them closer to the levels observed in the sham group (Figure 10I–N; Figure S48). These findings confirm that PtRhIr/Ru SAN@M effectively inhibits ferroptosis in ICH mice, disrupting the harmful feedback loop between ferroptosis and M_1_ microglial phenotype transformation. Chondroitin sulfate proteoglycan (CSPG) staining revealed significant astrocyte scar formation in the ICH group, evidenced by increased CSPG‐positive staining (Figure 10O; Figure S48) [25]. PtRhIr/Ru SAN@M treatment markedly reduced the CSPG staining, indicating decreased astrocyte scar formation. Inflammatory cytokines triggered by hematoma infiltration activate A_1_ reactive astrocytes. Astrocytes contribute to glial scar formation, help restore tissue integrity, and impede neuronal recovery. PtRhIr/Ru SAN@M reduced inflammation and oxidative stress while promoting microenvironmental remodeling. These combined effects likely underlie its ability to mitigate astrocyte scar formation and enhance neuronal recovery. To investigate the effects of PtRhIr/Ru SAN@M on microglial polarization in ICH mice, we performed immunofluorescence staining for Iba‐1, the M_1_ marker iNOS, and the M_2_ marker Arg‐1 in the injured region (Figure 10P–S). Compared to the Sham group, the ICH group exhibited significantly higher expression of both iNOS and Arg‐1, suggesting microglial activation. All drug‐loaded nanoparticle groups (PtRhIr@M and PtRhIr/Ru SAN@M) downregulated iNOS and upregulated Arg‐1. Notably, the PtRhIr/Ru SAN@M group showed a significantly higher number of Arg‐1^+^/Iba‐1^+^ double‐positive cells and fewer iNOS^+^/Iba‐1^+^ double‐positive cells than the PtRhIr@M group. In conclusion, microglia exhibit remarkable phenotypic plasticity in adapting to the inflammatory microenvironment of the brain. PtRhIr/Ru SAN@M can promote a shift from the pro‐inflammatory M_1_ phenotype to the anti‐inflammatory M_2_ phenotype, offering strong neuroprotection. This multifaceted approach reduces inflammation, inhibits ferroptosis, and promotes neuronal repair, making PtRhIr/Ru SAN@M a highly promising candidate for the treatment of ICH.

## 3 Conclusion

In conclusion, we report the successful development of PtRhIr/Ru SAN@M nanozymes that integrate multi‐enzyme‐like activity with the capacity to modulate microglial phenotypes. This innovative biomimetic nanocarrier exhibited excellent biocompatibility and efficiently targeted microglial injury sites, accumulating specifically in hemorrhagic regions to enable precise targeted treatment of damaged brain tissues. Our comprehensive findings demonstrate that PtRhIr/Ru SAN@M significantly enhanced the delivery of PtRhIr/Ru SAN across the BBB, leading to increased accumulation at the hematoma site. Given that ROS generated from hematoma and damaged tissue cause severe oxidative damage, we showed that PtRhIr/Ru SAN@M significantly reduced oxidative stress both in vitro and in vivo. This reduction in oxidative stress is critical for mitigating SBI following ICH. Furthermore, PtRhIr/Ru SAN@M effectively downregulates the production of neurotoxic cytokines, including IL‐1β, IL‐6, and TNF‐α. This downregulation is essential for reducing inflammation and promoting a favorable microenvironment for neuronal recovery. The significant improvement in functional outcomes and tissue repair observed in our study underscores the potential of PtRhIr/Ru SAN@M for the treatment of ICH. The multifaceted protective actions of PtRhIr/Ru SAN@M, including the attenuation of oxidative stress, modulation of microglial polarization, inhibition of ferroptosis, and promotion of neuronal repair, suggest that it is a highly promising candidate for developing neuroprotective therapies against both ischemic and hemorrhagic brain injuries. The combination of efficient BBB penetration, targeted delivery to inflamed brain regions, and minimal systemic toxicity renders PtRhIr/Ru SAN@M a promising therapeutic platform. We believe that PtRhIr/Ru SAN@M has the potential to transform the treatment of ICH and other neurodegenerative conditions, offering new hope to patients with these debilitating diseases.

## 4 Experimental Section

### 4.1 SOD‐Like Activity

The SOD‐like activity of the PtRhIr/Ru SAN was evaluated using a WST‐based assay. Nanoparticles were diluted to 0–48 µg/mL in assay buffer. Aliquots of 20 µL sample were added to a 96‐well plate containing 200 µL of WST solution. The reaction was initiated by adding 20 µL of enzyme solution. Samples were incubated at 37°C for 30 min, and the absorbance was measured at 350–525 nm using a Synergy H1 reader.

### 4.2 •OH Scavenging Assay

The •OH‐scavenging activity of PtRhIr and PtRhIr/Ru SAN was determined using a TMB–H_2_O_2_ colorimetric assay in NaAc–HAc buffer (pH 4.5). Nanoparticles (0–48 µg/mL) were mixed with TMB, where H_2_O_2_ oxidation generated oxTMB. Absorbance was measured at 550–750 nm using a Synergy H1 reader. To further confirm •OH scavenging, ESR analysis was performed using DMPO as a spin trap. A mixture containing 24 µg/mL PtRhIr/Ru SAN, 10 µm FeCl_3_, 1 mm H_2_O_2_, and 100 mm DMPO was prepared and transferred to a quartz tube for ESR detection.

### 4.3 CAT‐Like Activity Assay

PtRhIr/Ru SAN nanoparticles were diluted in PBS (pH 7.4) to final concentrations of 0–24 µg/mL. A YSI 5100 dissolved oxygen meter equipped with a Clark‐type electrode was calibrated prior to use. For each assay, 10 mL of 0.3% H_2_O_2_ was added to the measurement chamber. The reaction was initiated by introducing 10 µL of the nanoparticle suspension. Oxygen production was recorded for 5 min, with readings collected every 10 s.

### 4.4 Cell Modeling of ICH and Grouping

BV‐2 and HT‐22 cells treated with 10 mm hemin to establish an in vitro ICH model. Cells were assigned to four groups: (1) Control: Cultured under standard conditions. (2) Hemin: Treated with 10 mm hemin plus PBS for 12 h. (3) PtRhIr@M: hemin‐treated cells incubated with PtRhIr@M for 12 h. (4) PtRhIr/Ru SAN@M: hemin‐treated cells incubated with PtRhIr/Ru SAN@M for 12 h.

### 4.5 Cellular Uptake and Lysosomal Colocalization

Cells (1 × 10^5^ per glass‐bottom dish) were incubated with Cy5‐labeled PtRhIr/Ru SAN@M (24 µg/mL) for 0, 1, 2, and 4 h. After incubation, cells were stained with Hoechst 33342 and LysoTracker Green for 30 min in the dark. Samples were then washed with PBS and imaged using a Zeiss 800 confocal laser‐scanning microscope. Images were acquired from randomly selected fields.

### 4.6 Cell Viability Analysis

Cytotoxicity and cell viability were assessed using the CCK‐8 assay. BV2 and HT22 cells were seeded for 24 h, then treated with varying concentrations of PtRhIr@M or PtRhIr/Ru SAN@M for 24 h. After treatment, the medium was replaced with fresh medium containing 10 µL of CCK‐8 solution. Absorbance at 450 nm was recorded using a microplate reader.

### 4.7 Viability Assessment under ICH‐Mimicking Conditions

Hemin‐treated BV2 and HT22 cells were exposed to PtRhIr@M or PtRhIr/Ru SAN@M (0–48 µg/mL) for 24 h. Cell viability was determined using the CCK‐8 assay, as described above, and expressed relative to untreated controls.

### 4.8 Live/Dead Staining

To visualize treatment‐induced cytotoxicity, BV2 and HT22 cells were plated in confocal dishes, treated with PtRhIr/Ru SAN@M for 24 h, and stained with Calcein‐AM and PI for 20 min. Fluorescence images were acquired using a confocal microscope.

### 4.9 In Vitro ROS Detection

BV2 and HT22 cells were seeded and incubated for 24 h before treatment with the indicated formulations. For superoxide (•O2‐) detection, cells were stained with dihydroethidium for 30 min in the dark, washed with PBS, and imaged using CLSM.

For hydroxyl radical (•OH) detection, cells were incubated with the O27 probe under the same conditions, washed with PBS, and imaged.

For total intracellular ROS, cells were stained with 10 µm DCFH‐DA and 10 µm Hoechst for 20 min, rinsed with DMEM, and imaged by CLSM.

### 4.10 Immunofluorescence Analysis

BV2 and HT22 cells were seeded in confocal dishes, incubated for 24 h, and treated with the corresponding formulations for 12 h. After fixation and blocking, cells were incubated with primary antibodies against:

BV2 cells: Cyt c, GPX4, 4‐HNE, iNOS, IL‐6, CD86, CD206

HT22 cells: Cyt c, GPX4, 4‐HNE

Fluorescent secondary antibodies were applied, and signals were visualized using confocal microscopy to assess protein expression and subcellular localization.

### 4.11 TEM of Mitochondrial Ultrastructure

Cells were seeded in 6‐well plates and treated with the indicated formulations. After washing with PBS, cells were fixed with 3% butyl glycol at 4°C for 2 h, followed by post‐fixation in 1% osmium tetroxide for 1 h. Samples were rinsed with PBS, dehydrated through a graded series of organic solvents, and processed using critical‐point drying. The dried specimens were coated and examined by TEM to visualize mitochondrial ultrastructure.

### 4.12 Western Blot Analysis

Protein expression levels of HO‐1, FTH1, GPX4, SLC7A11, ACSL4, SPHK1, and p‐mTOR were analyzed by western blotting. Cells from the Control, Hemin, PtRhIr@M, and PtRhIr/Ru SAN@M groups were collected and lysed in a buffer containing protease inhibitors. Total protein was extracted, separated by sodium dodecyl sulfate‐polyacrylamide gel electrophoresis, and transferred onto polyvinylidene difluoride membranes. Membranes were blocked with 5% non‐fat milk, incubated with primary antibodies overnight at 4°C, and then with HRP‐conjugated secondary antibodies. Protein bands were visualized using an ECL system, and band intensities were quantified by densitometry and normalized to β‐actin.

### 4.13 In Vitro BBB Transport Assay

An in vitro BBB model was established using Transwell inserts. bEnd.3 cells were seeded in the upper chamber to form a confluent barrier, whereas BV2 cells were cultured in the lower chamber. Cy5.5‐labeled PtRhIr/Ru SAN@M was added to the upper chamber and incubated for 6 or 24 h. After incubation, the fluorescence in both chambers was quantified using a PerkinElmer IVIS Lumina III system to evaluate nanoparticle transport across the barrier.

### 4.14 Animal Grouping and Treatment

All animal procedures adhered to institutional guidelines and were approved by the Ethics Committee of Fujian Medical University (Approval Number: IACUC FJMU2022‐0608). An ICH model was established via collagenase injection. Two hours after induction, animals were randomized into four groups and treated via tail vein injection: (1) ICH + PtRhIr/Ru SAN@M group: 1.2 mg/kg; (2) ICH + PtRhIr@M group: same dosage as PtRhIr/Ru SAN @M, (3) ICH group: equal volume of normal saline, and (4) same surgical procedures without collagenase injection, followed by normal saline injection.

### 4.15 Hematological, Biochemical, and Histological Analyses

To assess systemic responses to treatment, blood samples were collected from the retro‐orbital venous plexus at baseline (day −1) and on days 1, 3, 5, 7, and 14 after PtRhIr/Ru SAN@M administration for routine hematological and biochemical analysis. After 21 days of treatment, the mice were euthanized, and the major organs, including the heart, liver, spleen, lungs, kidneys, and brain (ICH lesion region), were harvested for histological evaluation.

### 4.16 Y‐Maze Test

The Y‐maze consisted of three arms (A, B, and C). Each mouse was placed at the end of arm A and allowed to explore freely for 8 min. Arm entry was defined as all four paws into the arm. A spontaneous alternation was recorded when the mouse entered three different arms consecutively (e.g., A→B→C). The alternation percentage was calculated as: Alternation (%) [number of spontaneous alternations ÷ (total arm entries − 2)] × 100%.

### 4.17 OFT Analysis

The OFT was used to assess locomotor activity and anxiety‐like behaviors. Each mouse was placed in a square arena, and its movements were recorded for 300 s. The total distance traveled and the time spent in the center versus the periphery were quantified. Grid crossings, defined as full passages over intersection lines, were counted throughout each session. Locomotor activity was classified as low, medium, or high, based on the crossing frequency derived from the group distributions.

### 4.18 MWM Analysis

The MWM was used to evaluate spatial learning and memory. Mice were trained in a circular pool filled with opaque water with a hidden platform submerged in quadrant 4. Training continued for 5 days, and the escape latency was recorded during each 120‐s trial. On day six, the platform was removed for probe testing. Memory performance was assessed based on the time spent in the target quadrant and the number of platform crossings.

### 4.19 In Vivo Therapeutic Efficacy

Mice were euthanized 21 days after ICH induction. After transcardial perfusion with cold saline and fixation with paraformaldehyde, the brains were harvested, postfixed overnight, and sectioned. Histopathology was assessed using H&E and Nissl staining. Intracerebral ROS levels were measured with fluorescent probes, and TUNEL staining was performed to identify apoptotic cells. Immunofluorescence was used to evaluate the expression of iNOS/Iba‐1, Arg‐1/Iba‐1, SLC7A11, GPX4, ACSL4, SPHK1, p‐mTOR, and CSPG. Sections were blocked, incubated with specific primary antibodies, and exposed to fluorescent secondary antibodies. Fluorescence images were obtained by confocal microscopy to assess protein expression within the ICH lesion area.

### 4.20 Statistical Analysis

All experiments were expressed as mean ± S.D. Statistical analyses were performed using Origin 2024 software and Image J. For experiments requiring statistical analysis, at least three separate experiments were conducted (*n* ≥ 3). Statistical significance was assessed using a two‐tailed Student's *t*‐test, with a threshold of *p <* 0.05 considered statistically significant. The levels of significance are denoted as follows: *p <* 0.05 (^*^), *p <* 0.01 (^**^), *p <* 0.001 (^***^). Unless otherwise specified, all comparisons were made relative to the control group.

## Author Contributions

J. L., P. W., and Y. P. contributed equally to this study. Y. G., Y. Z., and D. W. conceived and supervised the study. J. L., P. W., and Y. P. contributed to the writing of the article and specific experiments. H. Z., J. H., Q. C., and Z. S. analyzed the data. Y. H., J. W., F. L., and F. C. provided support for cell and animal experiments. C. D., W. F., Y. L., and D. K. corrected the grammar in the text and provided writing advice.

## Conflicts of Interest

The authors declare no conflict of interest.

## Supporting information




**Supporting File**: smll72255‐sup‐0001‐SuppMat.docx.

## Data Availability

The data that support the findings of this study are available in the supplementary material of this article.
