# Supplementary material for: Microglial Membranes Wrapped Ultrasmall Medium‐Entropy Ru Single‐Atom Nanozyme: Enhanced Catalysis for Accelerating Inflammation/Redox Microenvironment Regulation in Intracerebral Hemorrhage
Source: Adv Sci (Weinh). 2026 Jan 4;13(18):e20714. doi: 10.1002/advs.202520714 (PMC13042521; doi:10.1002/advs.202520714)
Supplement: Supplementary file 1 — Supporting File: smll72255‐sup‐0001‐SuppMat.docx. [file ADVS-13-e20714-s001.docx]

Supporting Information

**Microglial Membranes Wrapped Ultrasmall Medium-Entropy Ru Single-Atom Nanozyme: Enhanced Catalysis for Accelerating Inflammation/Redox Microenvironment Regulation in Intracerebral Hemorrhage**

*Jiebo Li^1,2,3,4†^, Penghui Wei^1,2,3,4†^, Yuanbo Pan^5†^, Hongjia Zheng^1,2,3,4^, Jiajun Hu^1,2,3,4^, Qianxi Chen^1,2,3,4^, Zhongyuan Shen^1,2,3,4^, Yongrui Hu^1,2,3,4^, Jiajun Wu^1,2,3,4^, Fuxin Lin^1,2,3,4^, Fuxiang Chen^1,2,3,4^, Chenyu Ding^1,2,3,4^, Wenhua Fang^1,2,3,4^, Yuanxiang Lin^1,2,3,4^, Dezhi Kang^1,2,3,4^, Yuxiang Gu^1,2^*, Yang Zhu**^1,2,3,4^*, Dengliang Wang^1,2,3,4^**

**Experimental Procedures**

**Chemicals**

All experiments were performed with high-quality reagents and materials. Ruthenium trichloride (RuCl_3_), potassium hexachloroplatinate (IV) (K_2_PtCl_6_), rhodium chloride (RhCl_3_), iridous chloride (IrCl_3_), sodium borohydride (NaBH_4_), polyvinylpyrrolidone (PVP) are purchased from Sinopharm Chemical Reagents (Shanghai, China). Hemin was acquired from YuanYe BioTechnology (Shanghai, China), while Cy5.5 was obtained from Beyotime (Shanghai, China). The fixative for electron microscopy was supplied by Bergolin Biotechnology (Dalian, China). 3,3',5,5'-tetramethylbenzidine (TMB), propidium iodide (PI), and C11-BODIPY^581/591^ were sourced from Sigma-Aldrich (St. Louis, USA). Hoechst 33342, 2’,7’-dichlorofluorescin diacetate (DCFH-DA), the cell count kit-8 (CCK-8), and JC-1 were purchased from Beyotime (Shanghai, China). Antibodies against Glutathione Peroxidase 4 (GPX4), Anti-Cytochrome c antibody (Cyt c), 4-Hydroxynonenal Antibody (4HNE), Anti-Ferritin heavy chain antibody (FTH1), Anti-Heme Oxygenase 1 antibody (HO-1), Anti-IL-6 antibody (IL-6), Anti-iNOS antibody (iNOS), Anti-NG2 antibody, Anti-FACL4 (ACSL4) and beta Actin and secondary antibodies goat anti-rabbit were procured from Abcam (Cambridge, UK); Antibodies against Sphingosine Kinase 1 (SPHK1), Phospho-mammalian target of rapamycin (p-mTOR), CD86, CD206, Arginase (Arg)-1 and CD206 were procured from Thermo Fisher Scientific (Waltham, USA); Antibodies against xCT/SLC7A11 (SLC7A11) were procured from Cell Signaling Technology (Danvers, MA, USA). Deionized (DI) water was supplied by a Milli-Q water purification system.

**Instruments**

Powder X-ray diffraction patterns of samples were recorded on a Rigaku Miniflex-600 operating at 40 kV voltage and 15 mA current with Cu Kα radiation (λ=0.15406 nm). TEM images were taken by Hitachi-7700 working at 100 kV. The HAADF-STEM was recorded by JEM-ARM200F (JEOL) TEM/STEM with a spherical aberration corrector working at 200 kV. The EDS mapping was measured by JEM-2100F working at 200 kV. X-ray photoelectron spectroscopy (XPS) was collected on scanning X-ray microprobe (PHI 5000 Verasa, ULAC-PHI) using Al Ka radiation and the C1s peak at 284.8 eV as internal standard. XAFS spectra at the Ni K-edge was collected at BL14W1 station in Shanghai Synchrotron Radiation Facility (SSRF). The electron storage ring of SSRF was operated at 3.5 GeV with a maximum current of 250 mA. The Ni K-edge XANES data were recorded in a fluorescence mode. Ru foil was used as references. The UV absorbance of samples is measured by UV-vis spectrophotometer (8453, Agilent). Metal content analysis by using inductively coupled plasma mass spectrometer (ICP-MS, PlasmaQuad 3, Thermo Elemental). Dynamic light scattering (DLS) and Zeta potential were obtained by using a Brook haven. The cell apoptosis and necrosis were monitored by flow cytometer (CytoFLEX, Beckman). Oxygen levels were measured using an Oxygen Meter (Leici, China). Fluorescence imaging was performed with a Zeiss 800 confocal laser scanning microscope (CLSM). Luminescence data were collected using a SYNERGY H1 microplate reader (Biotek). In vivo optical and X-ray imaging of small animals were conducted with a PerkinElmer IVIS Lumina III system to explore biological interactions. Molecular separation and visualization were carried out by gel electrophoresis on a Bio-Rad electrophoresis analyzer, with imaging on a Bio-Rad ChemDoc XRS. Centrifugation was performed with an ultra-high-speed centrifuge (Eppendorf) to maintain sample integrity. Behavioral parameters were recorded using VisuTrack software (Xinruan Information Technology, Shanghai, China).

**Table S1. EXAFS data fitting results of Samples.**

| **Sample** | **Path** | **CN^a^** | **R(Å)^b^** | **σ^2^ (Å^2^)^c^** | **ΔE_0_(eV)^d^** | **R factor** |
| --- | --- | --- | --- | --- | --- | --- |
| **Ru K-edge (Ѕ_0_^2^=0.789)** | | | | | | |
| **Ru foil** | **Ru-Ru** | **12.0*** | **2.676±0.002** | **0.0038** | **4.8±0.6** | **0.0035** |
| **RuCl_3_** | **Ru-Cl** | **6.3±0.6** | **2.342±0.013** | **0.0051** | **-1.7±2.8** | **0.0100** |
| **RuO_2_** | **Ru-O** | **5.6±0.3** | **1.973±0.006** | **0.0030** | **0.9±1.5** | **0.0087** |
|  | **Ru-O-Ru** | **1.9±0.2** | **3.052±0.006** | **0.0027** | **11.1±1.5** |  |
|  | **Ru-O-Ru** | **8.7±0.3** | **3.624±0.005** |  |  |  |
| **PtRhIr/Ru SAN** | **Ru-N** | **2.9±0.2** | **1.990±0.015** | **0.0049** | **-6.0±2.7** | **0.0132** |

*^a^CN*, coordination number; *^b^R*, the distance between absorber and backscatter atoms; *^c^σ*^2^, the Debye Waller factor value; *^d^ΔE*_0_, inner potential correction to account for the difference in the inner potential between the sample and the reference compound; *R* factor indicates the goodness of the fit. *S*0^2^ was fixed to 0.789, according to the experimental EXAFS fit of Ru foil by fixing *CN* as the known crystallographic value. * This value was fixed during EXAFS fitting, based on the known structure of Ru. Fitting conditions: *k* range:3.0 - 10.0; *R* range: 1.0-3.0; fitting space: R space; *k*-weight = 2. A reasonable range of EXAFS fitting parameters: 0.700 < *Ѕ*_0_^2^ < 1.000; *CN >* 0; *σ*^2^ > 0 Å^2^; |Δ*E*_0_| < 15 eV; *R* factor < 0.02.


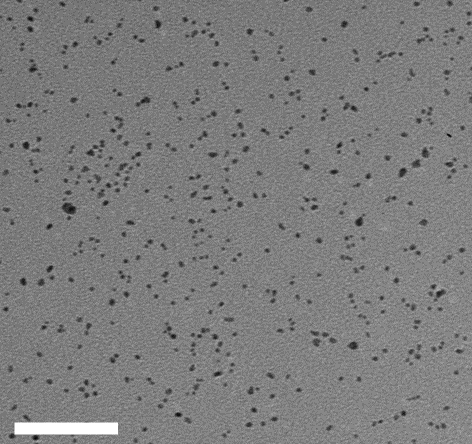


**Figure S1**. TEM image of PtRhIr alloy. Scale bar = 100 nm.


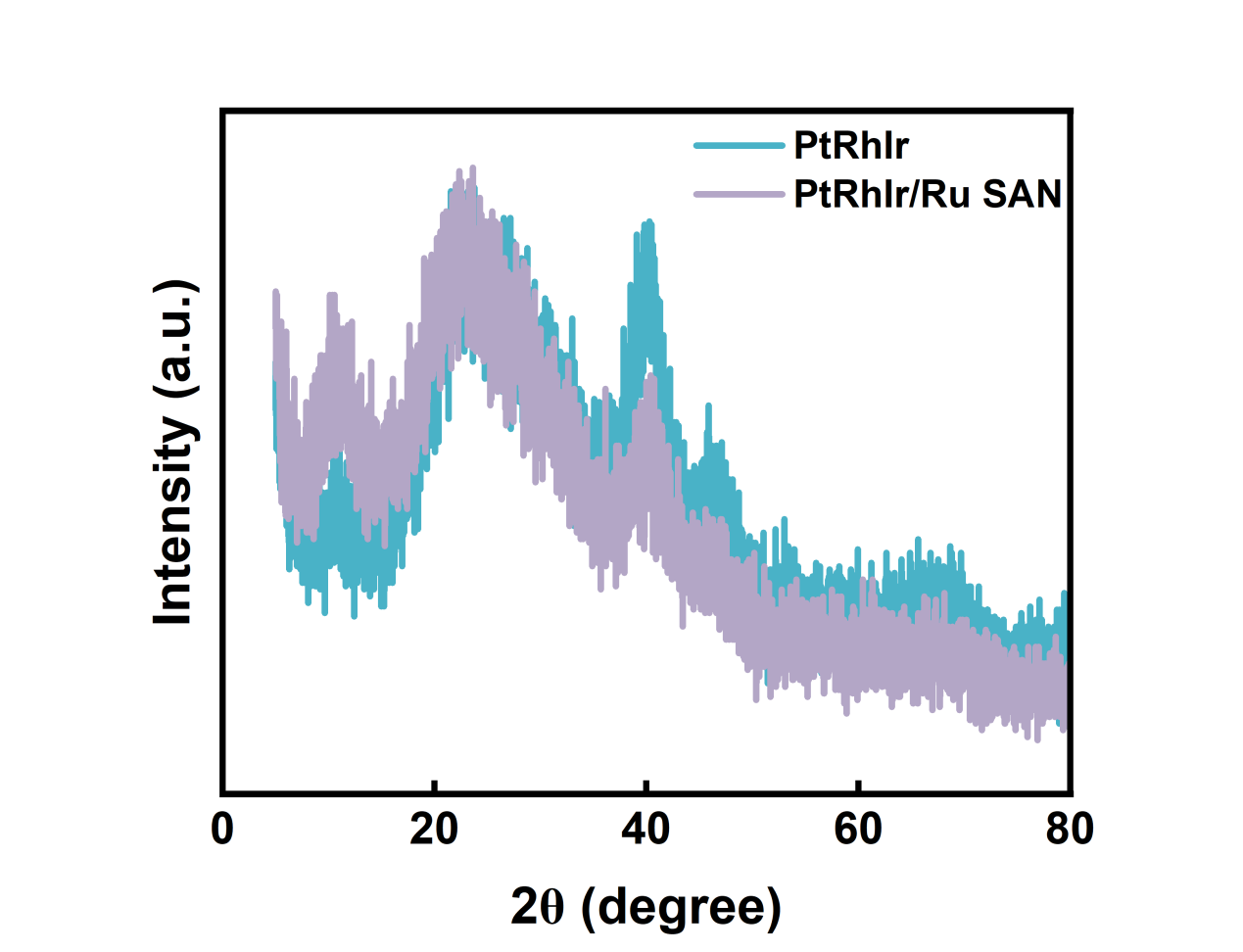


**Figure S2**. XRD spectrum of PtRhIr and PtRhIr/Ru SAN.


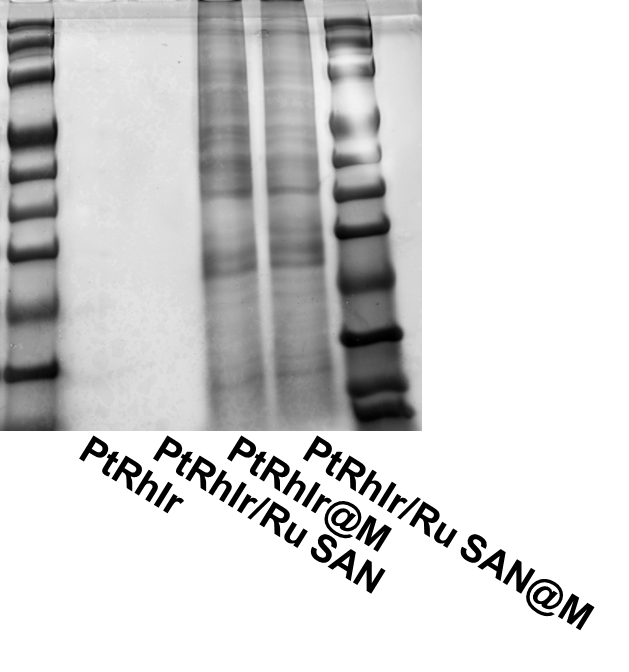
 **Figure S3**. SDS-PAGE analysis of total proteins isolated from PtRhIr and PtRhIr/Ru SAN.


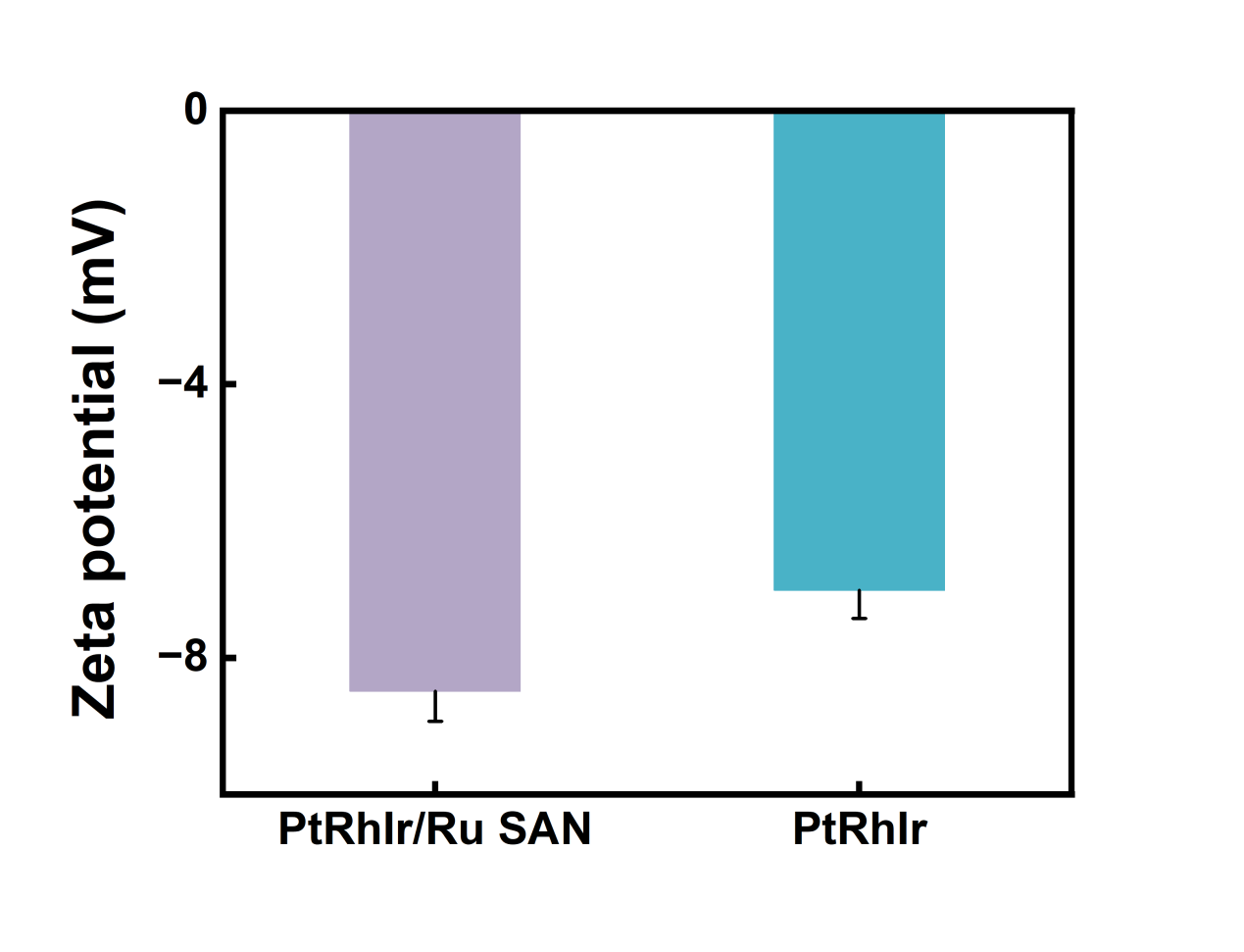


**Figure S4**. Zeta potential distribution of PtRhIr/Ru SAN and PtRhIr. (n = 3, for each group).


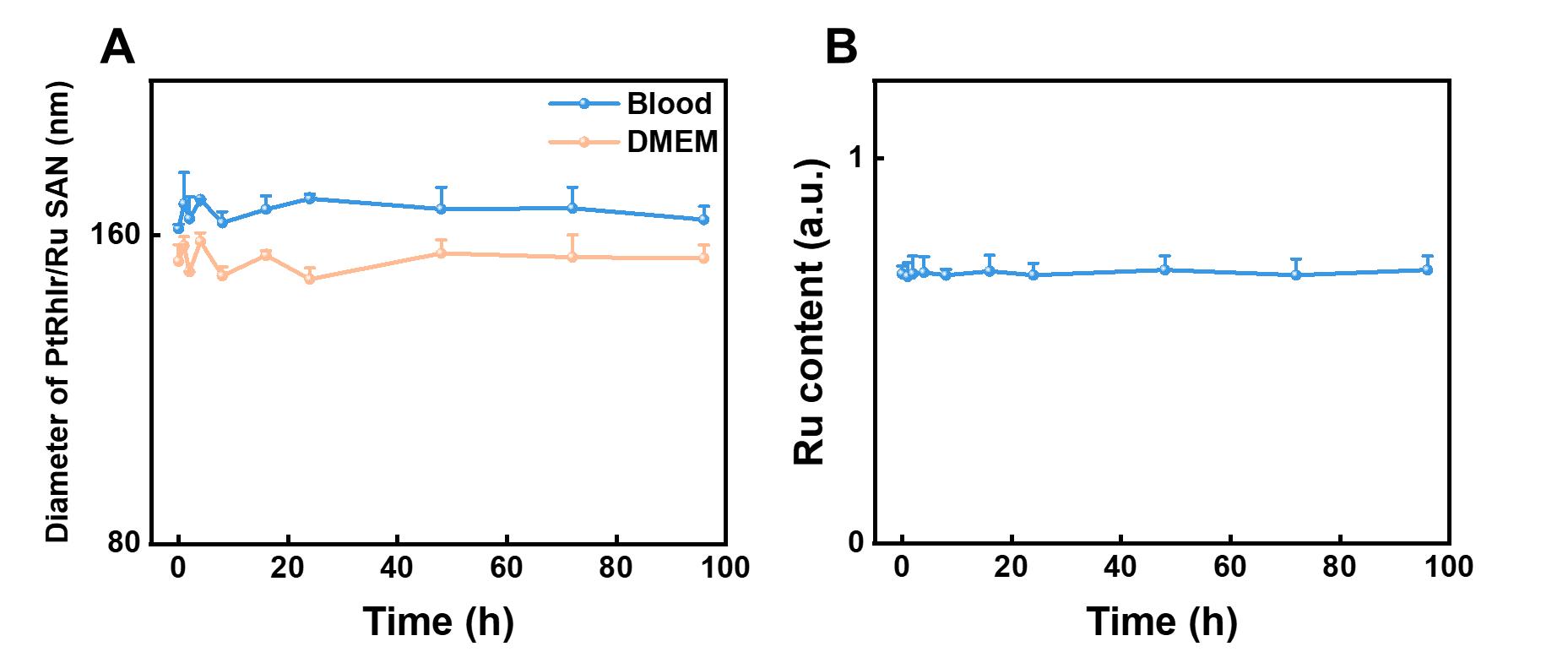


**Figure S5**. A) The long-term stability of PtRhIr/Ru SAN and B) Ru atoms leakage. (n = 3, for each group).

**
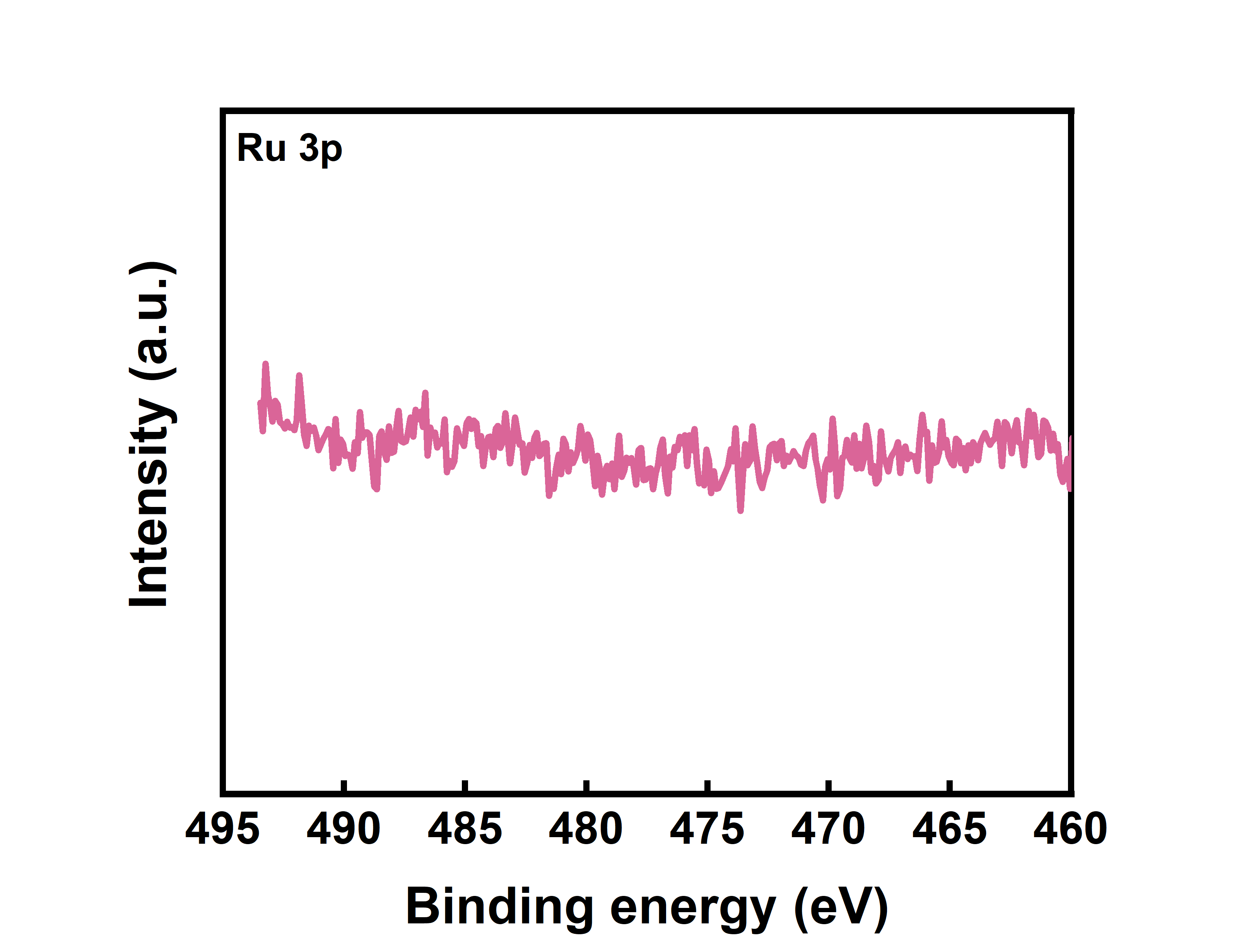
**

**Figure S6**. High-resolution XPS spectrum of PtRhIr/Ru SAN.


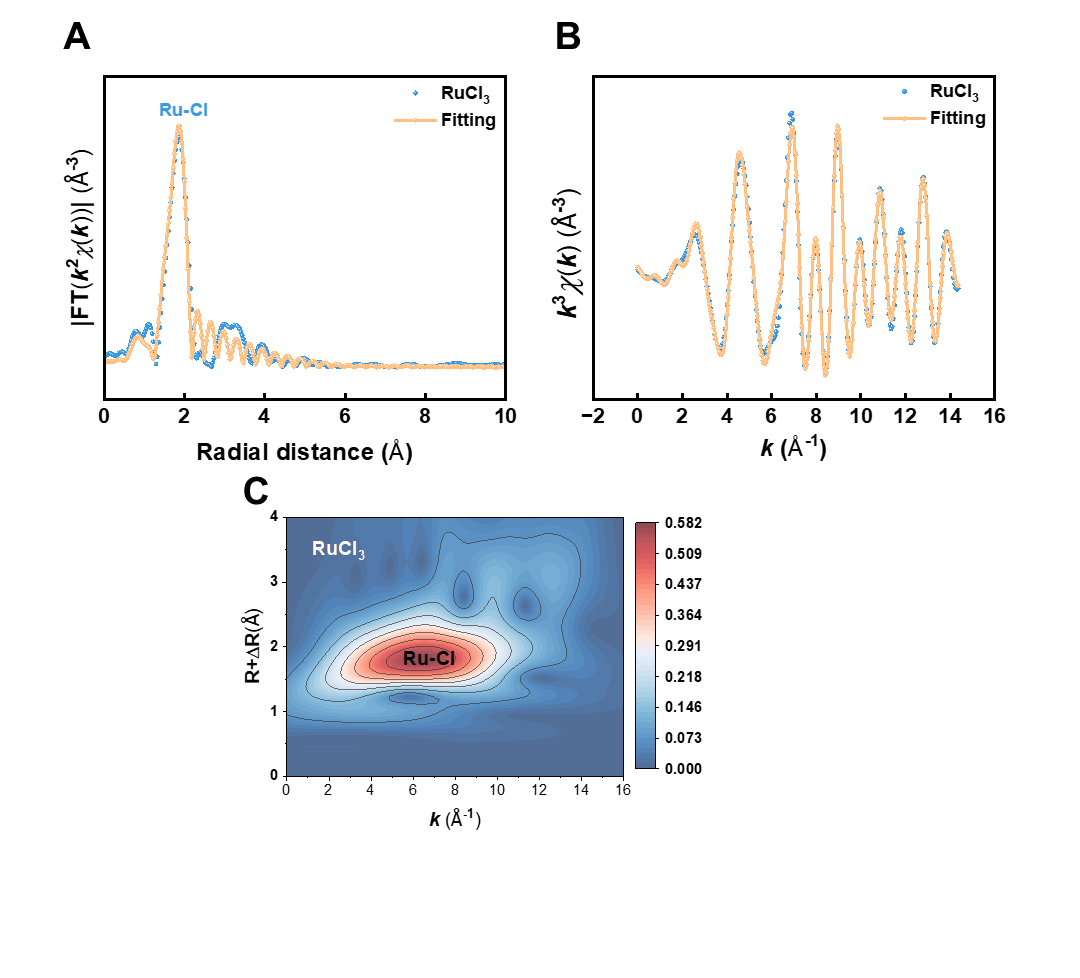


**Figure S7**. (D) EXAFS fitting curves of RuCl_3_ at the R space. (G) EXAFS fitting curve of RuCl_3_ at the k space. (J) Wavelet transformation of RuCl_3_.

.


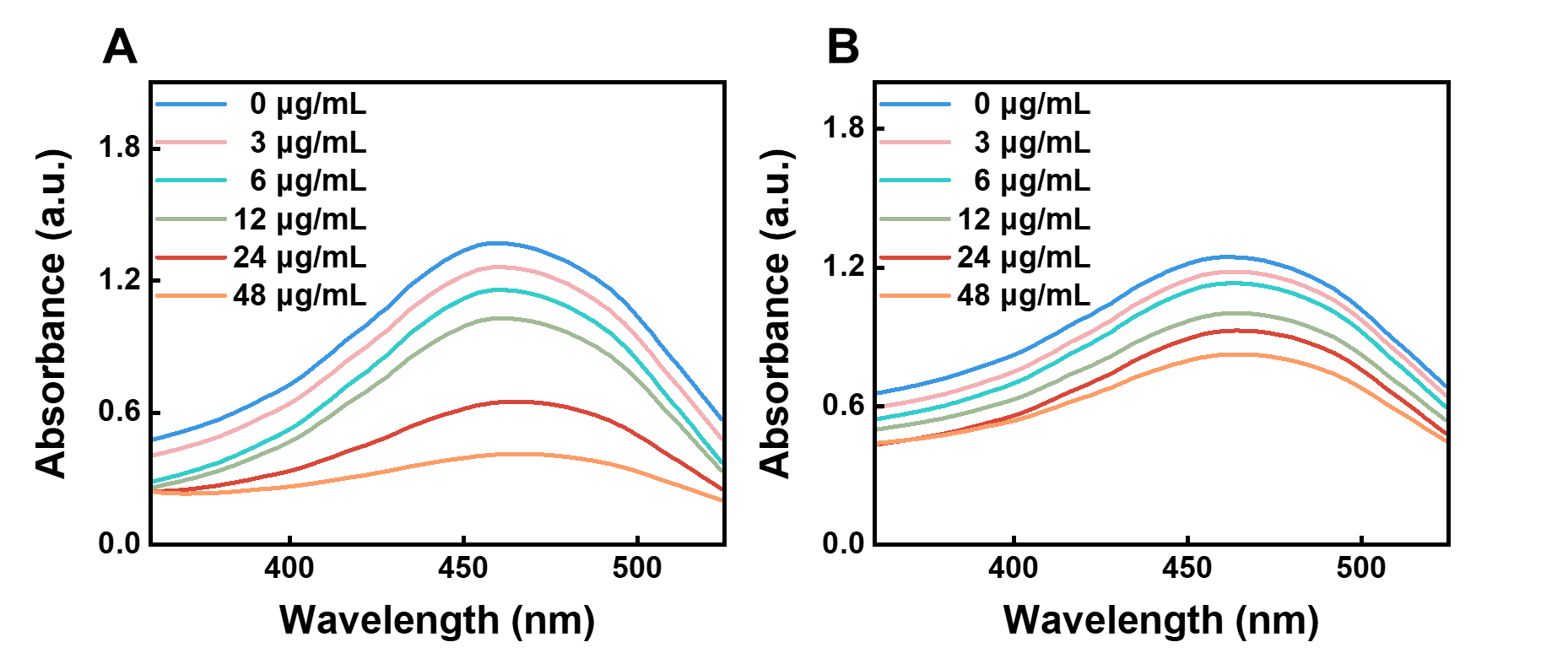


**Figure S8**. SOD-like activity of A) PtRhIr/Ru SAN and B) PtRhIr at different concentrations.


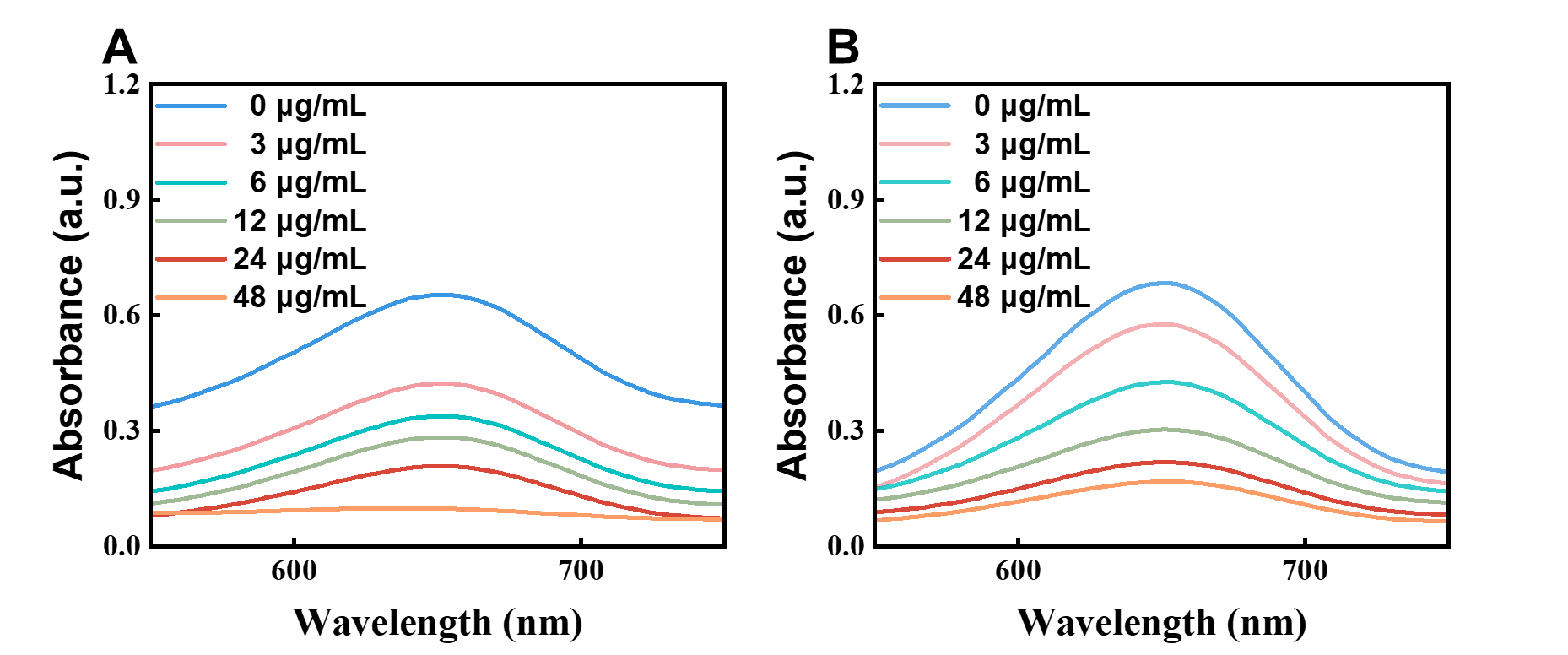


**Figure S9**. •OH scavenging activity of A) PtRhIr/Ru SAN and B) PtRhIr at different concentrations.


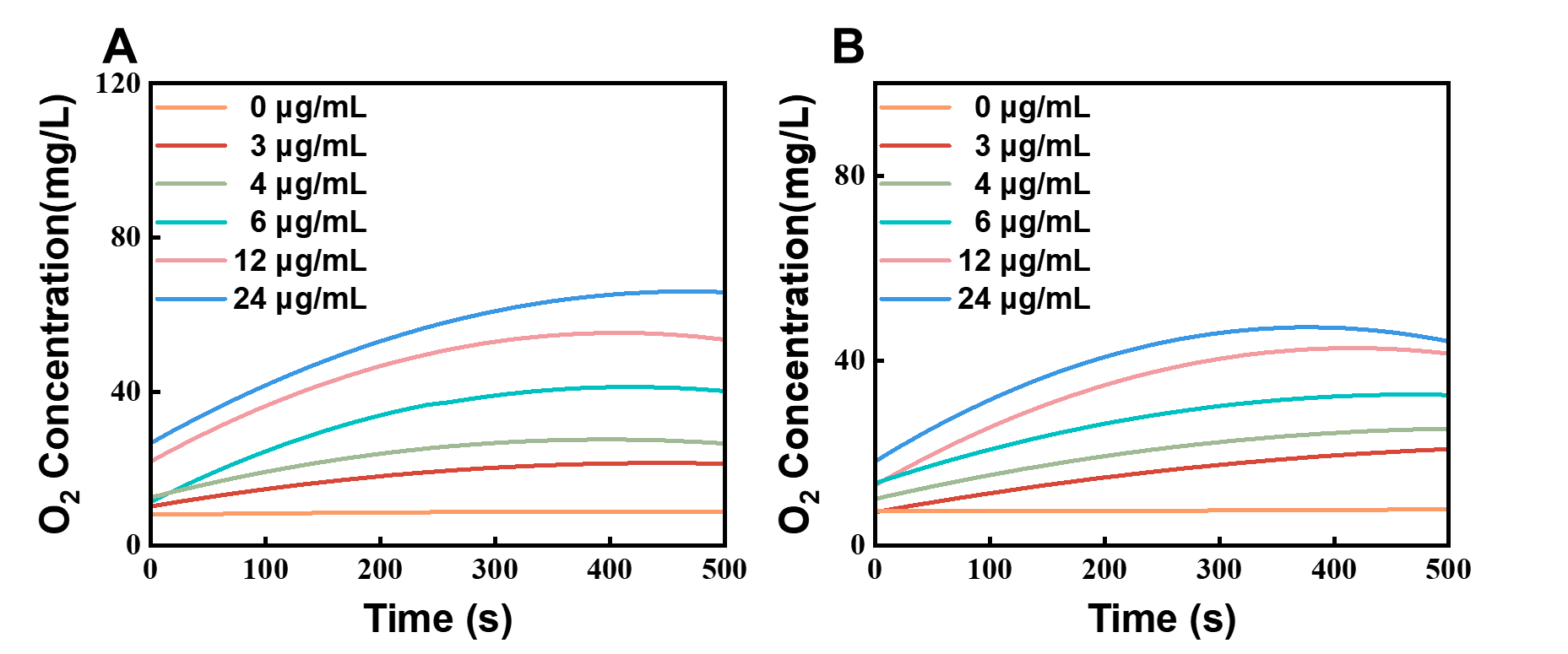


**Figure S10**. CAT-like activity of A) PtRhIr/Ru SAN and B) PtRhIr at different concentrations.


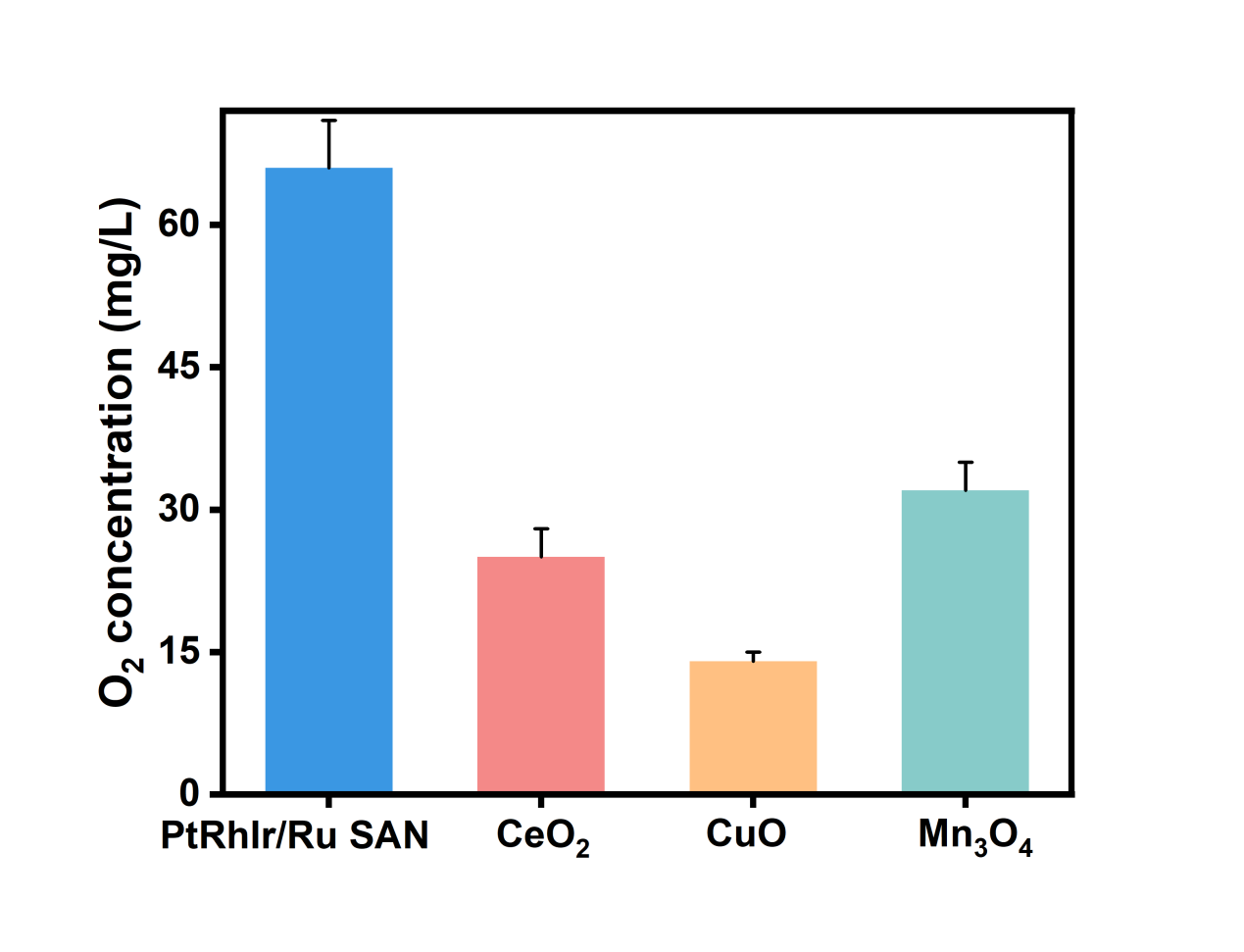


**Figure S11.** CAT-like Activity of PtRhIr/Ru SAN@M and Other Nanozymes. (n = 3, for each group).


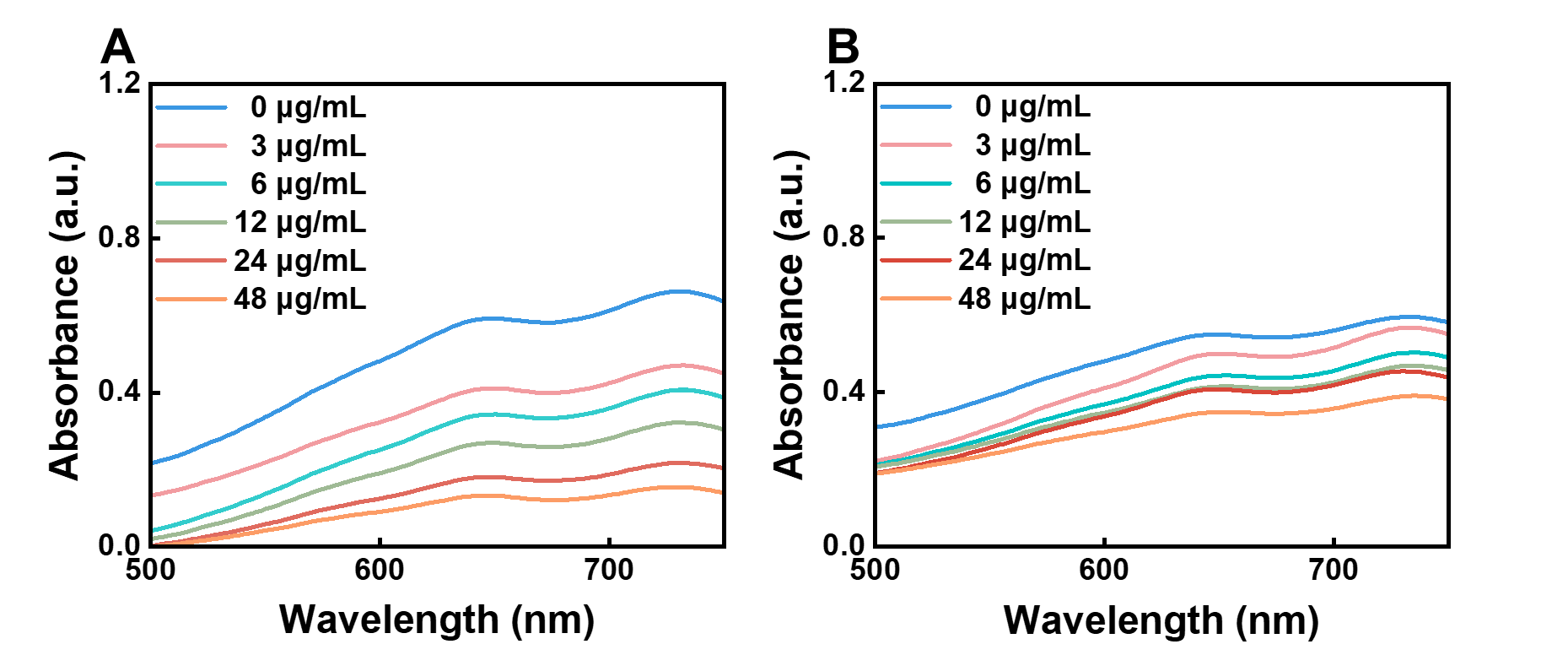


**Figure S12**. ABTS-like activity of A) PtRhIr/Ru SAN and B) PtRhIr at different concentrations.


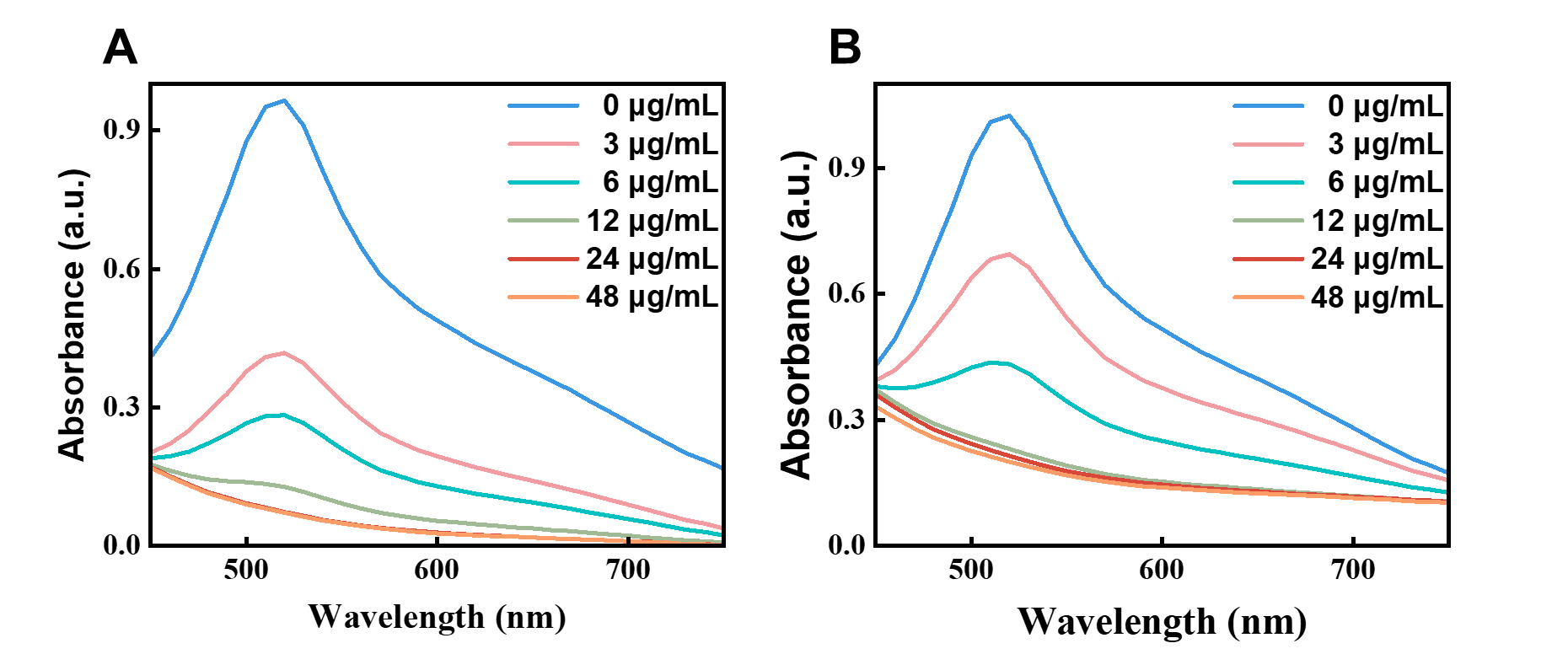


**Figure S13**. DPPH-like activity of A) PtRhIr/Ru SAN and B) PtRhIr at different concentrations.


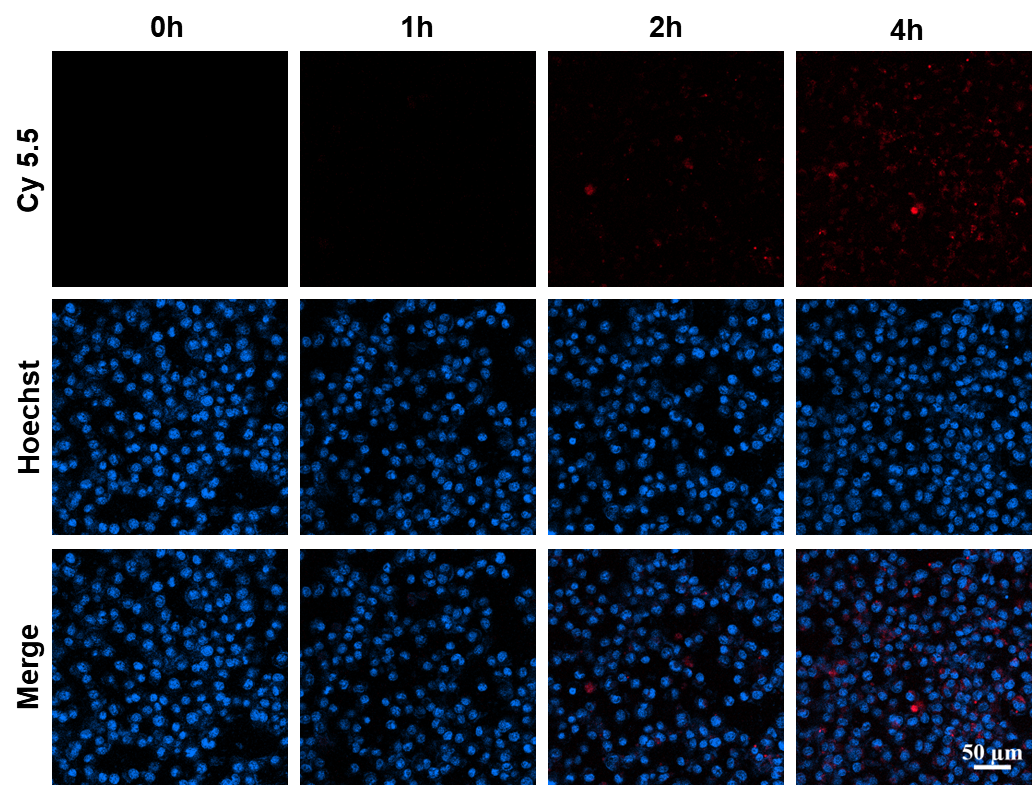


**Figure S14**. CLSM images showing cellular uptake in BV2 cells.


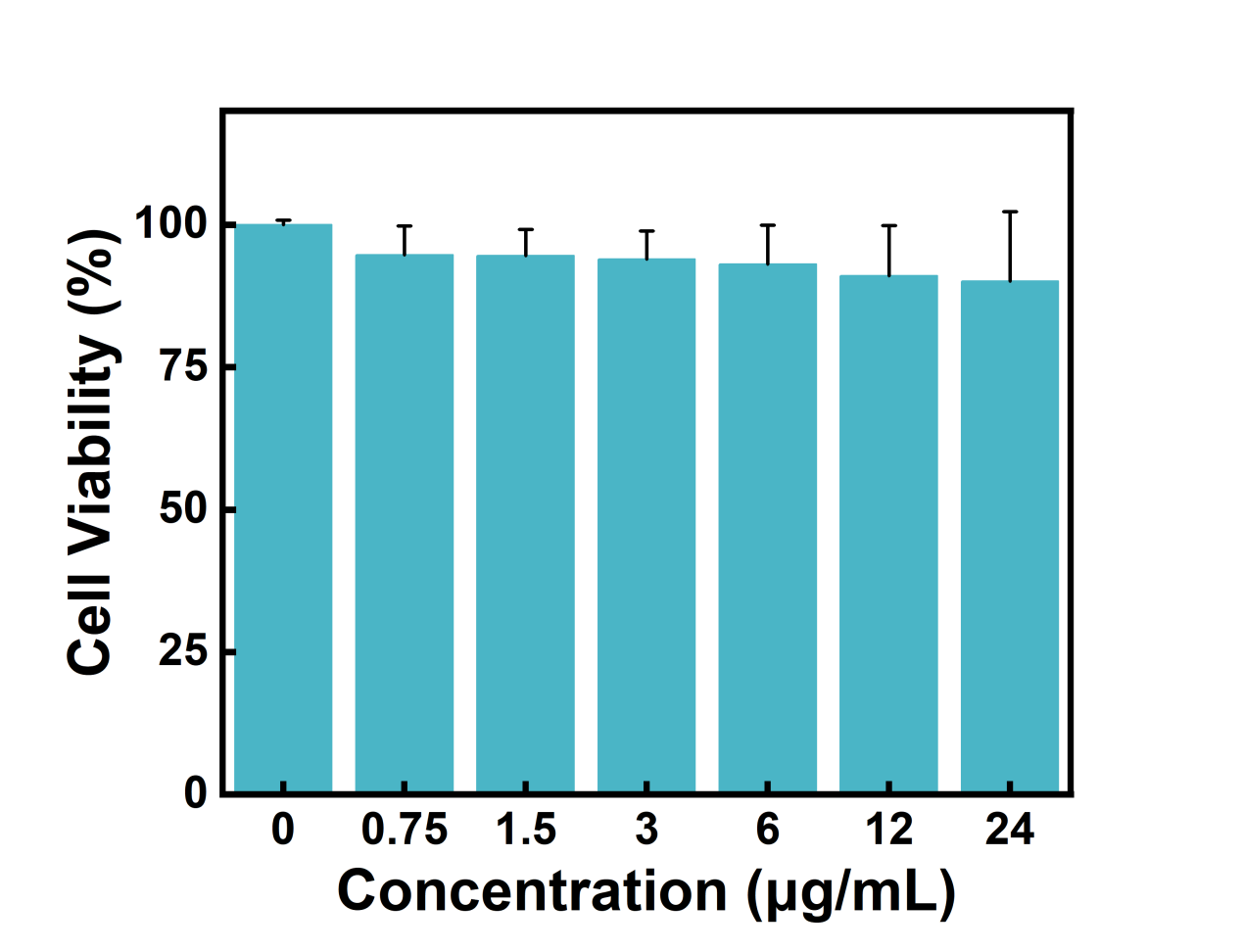
 **Figure S15**. Cell viability of BV2 cells treated with different concentrations of PtRhIr. (n = 4, for each group).


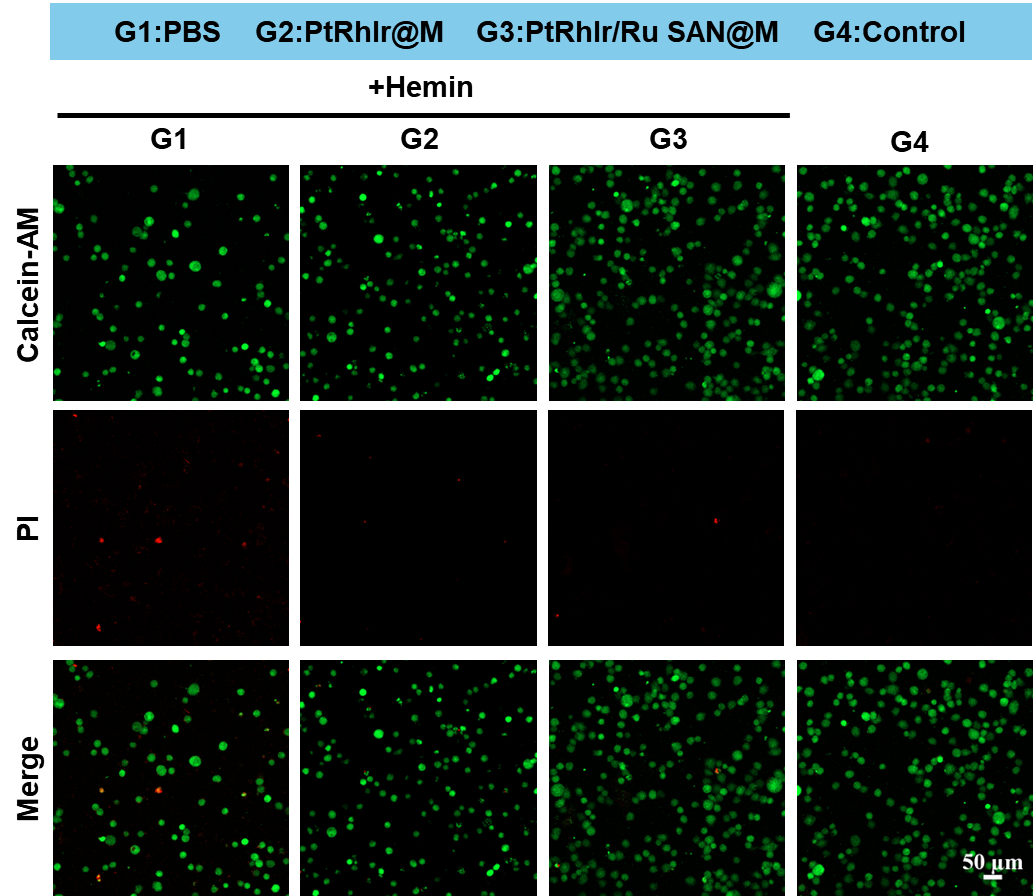


**Figure S16**. CLSM images depicting Calcein-AM/PI co-staining of BV2 cells with different treatment.


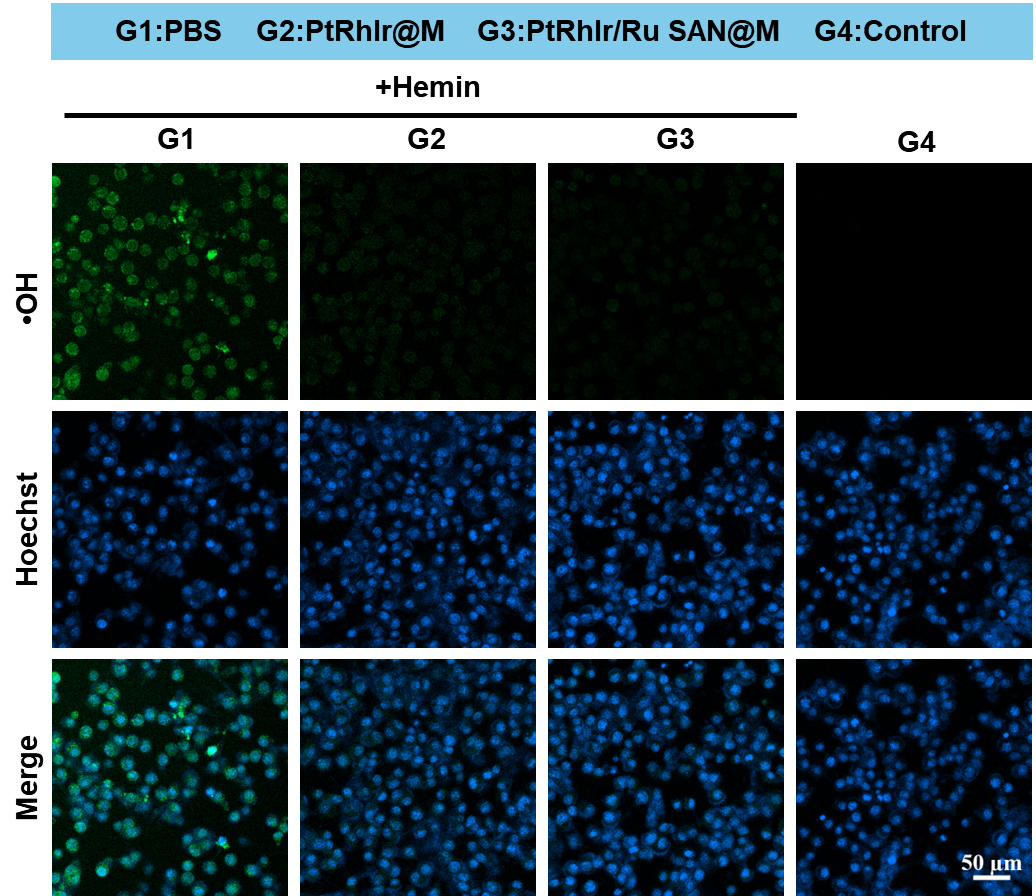


**Figure S17**. CLSM images of •OH staining in BV2 cells with different treatment.


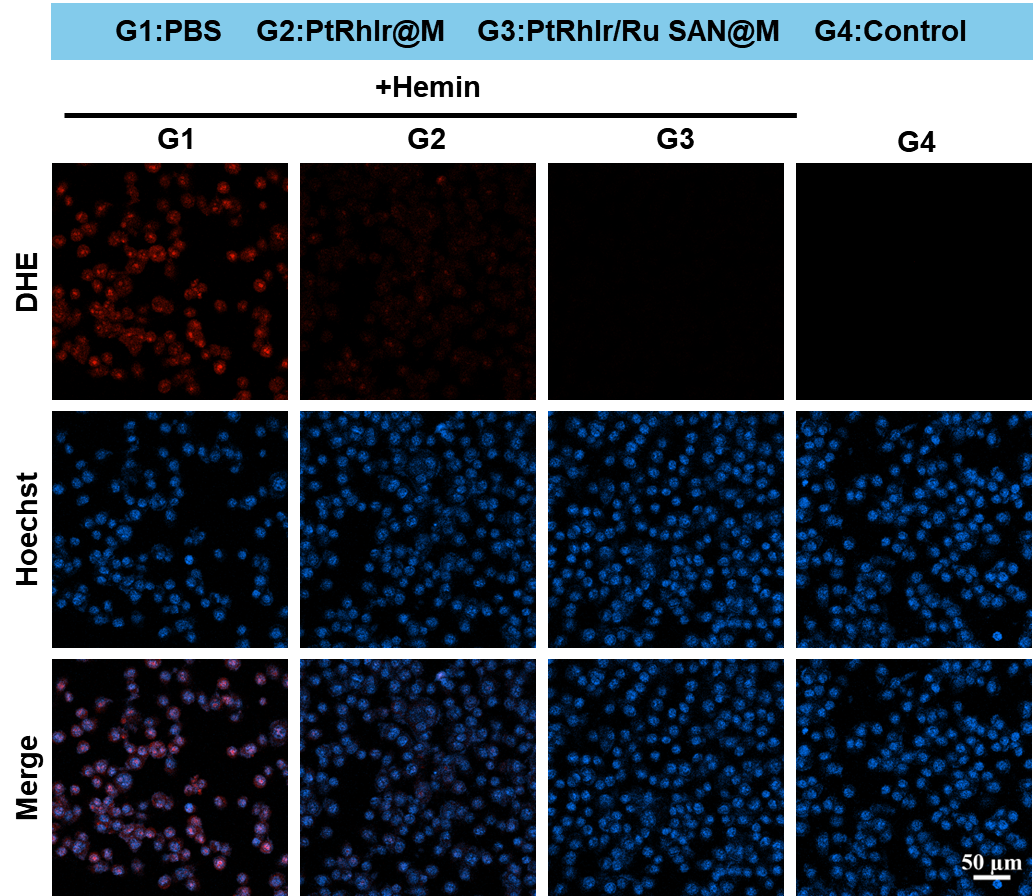


**Figure S18**. CLSM images of DHE staining in BV2 cells with different treatment.


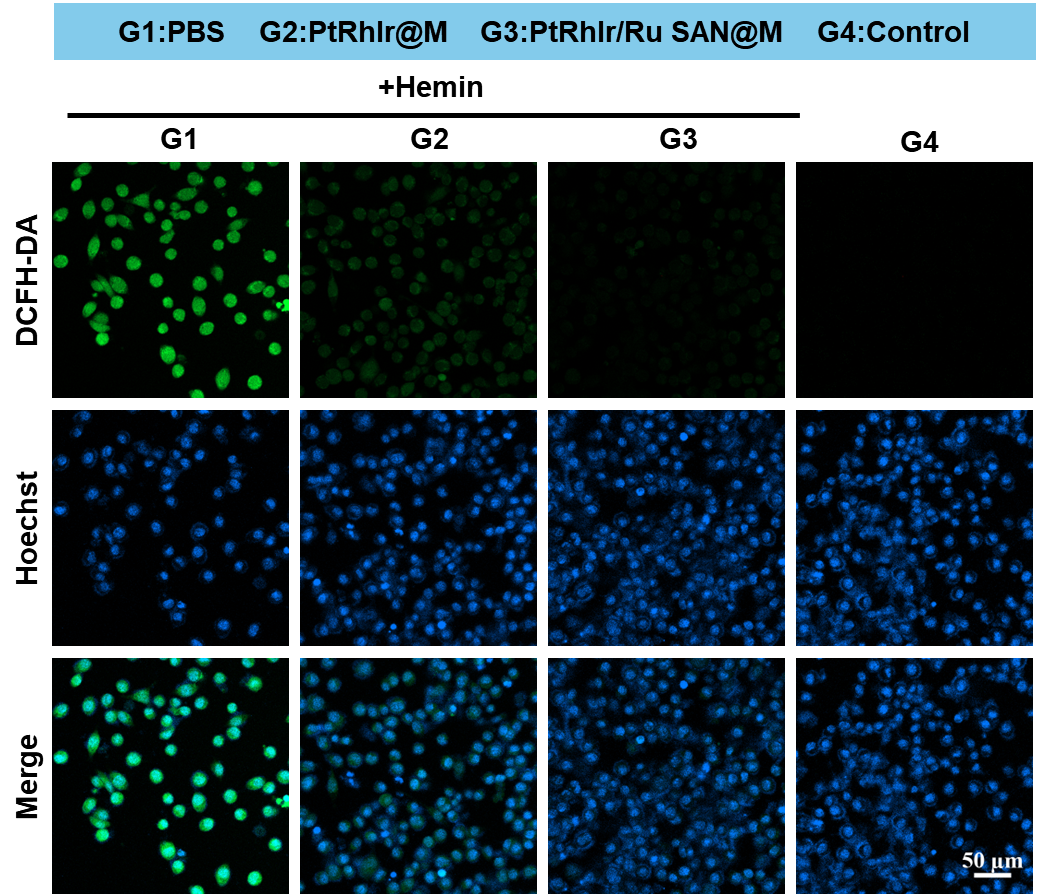


**Figure S19**. CLSM images of ROS staining in BV2 cells with different treatment.


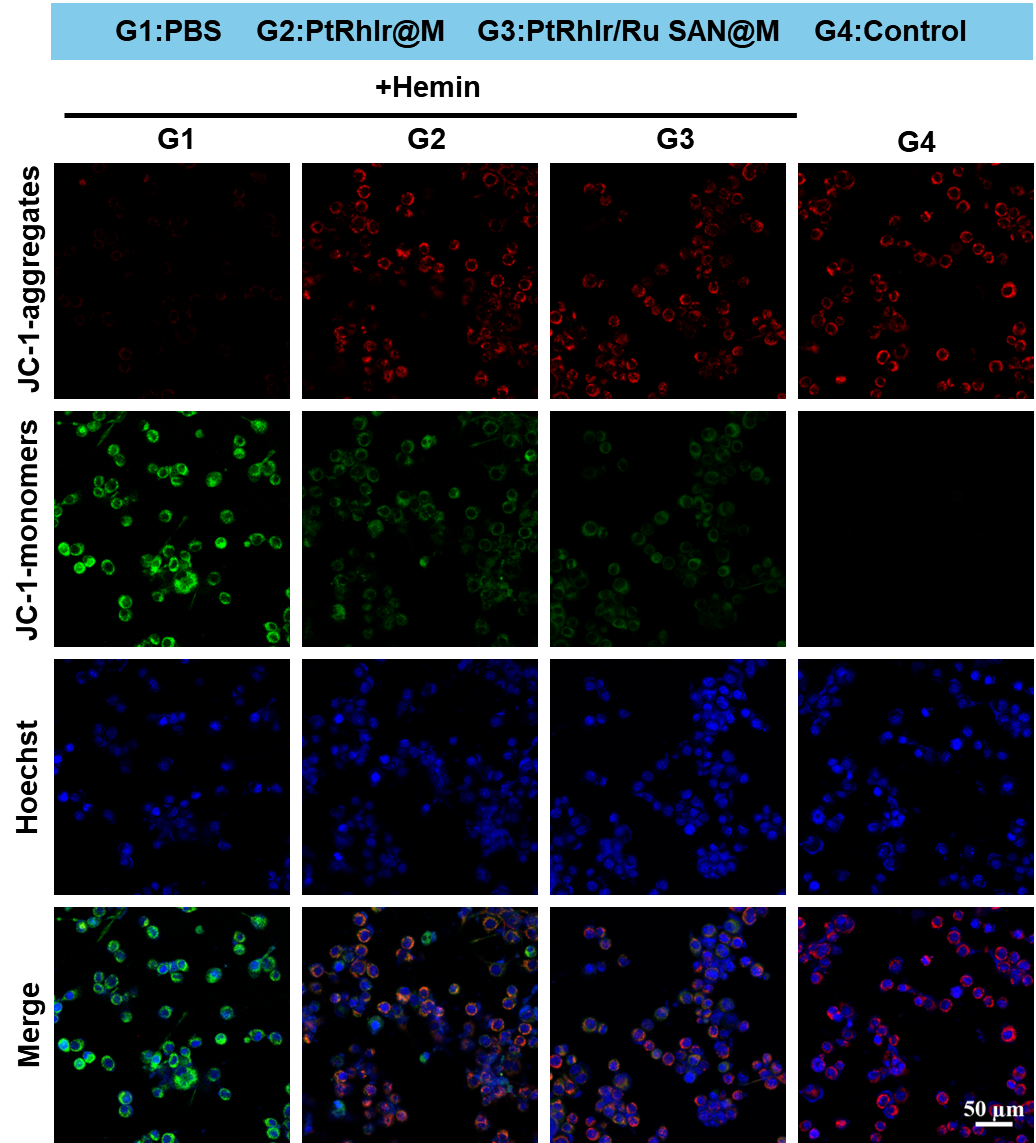


**Figure S20**. CLSM images of JC-1 staining in BV2 cells with different treatment.


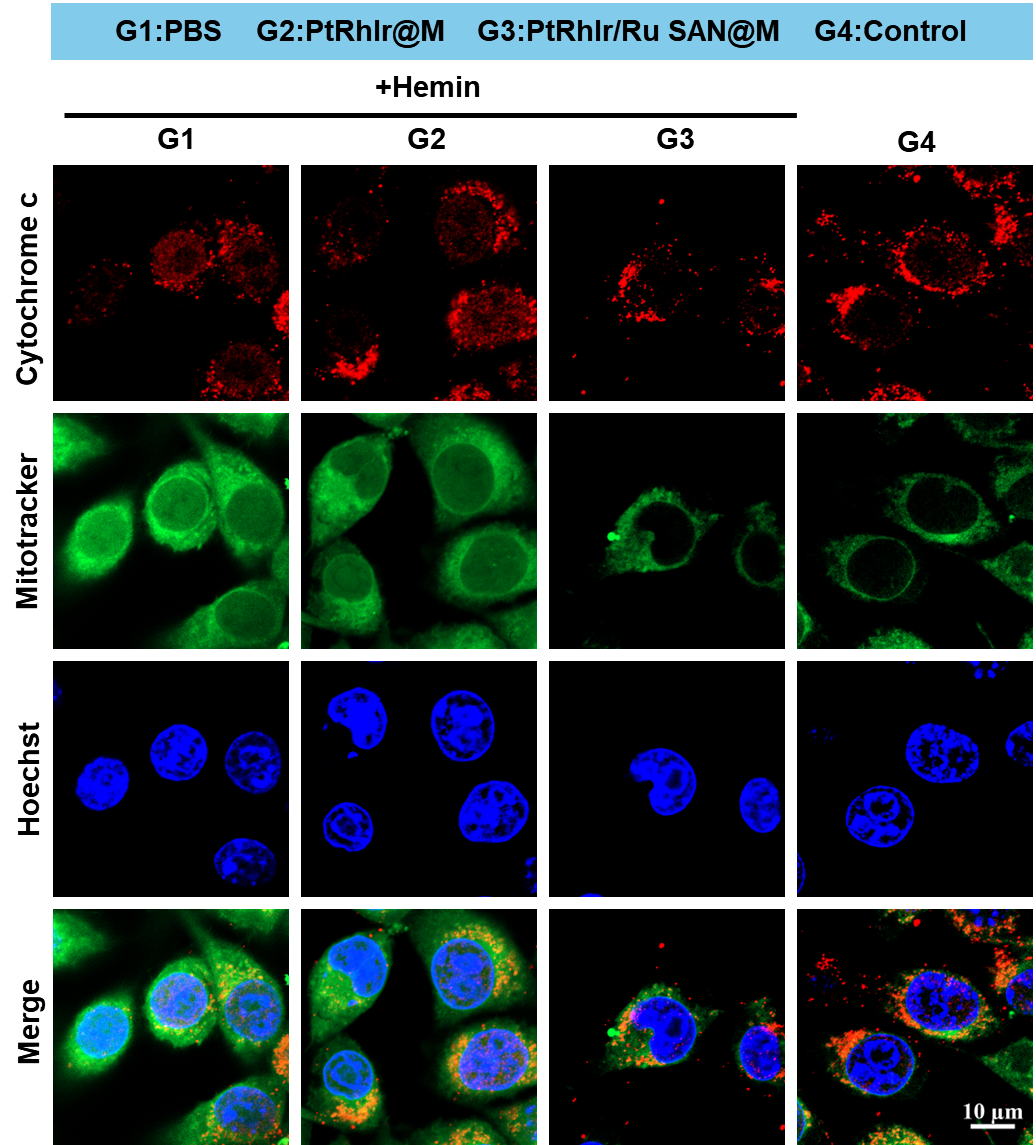


**Figure S21**. CLSM images of Cyt c staining in BV2 cells with different treatment.


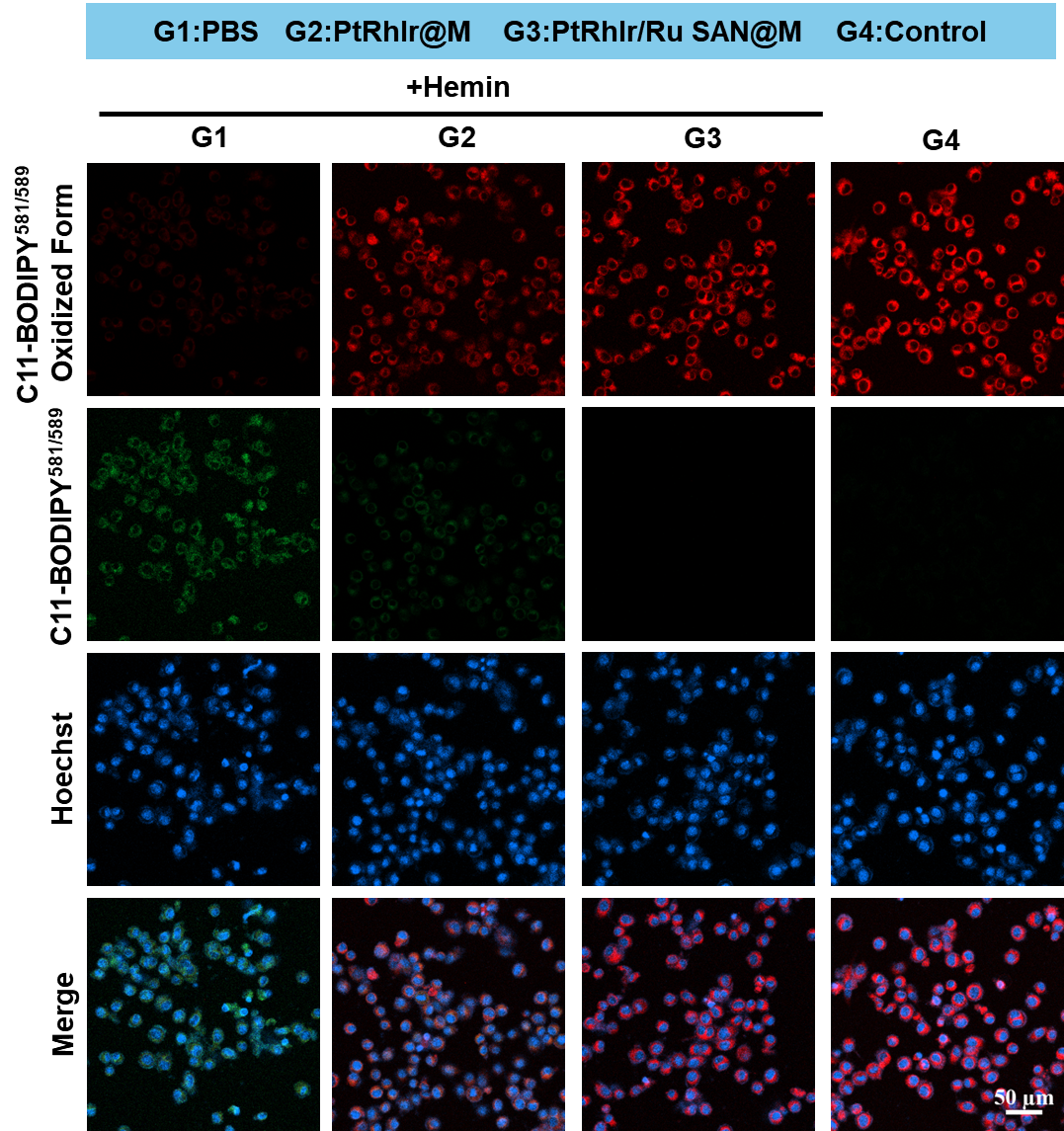


**Figure S22**. CLSM images of LPO staining in BV2 cells with different treatment.


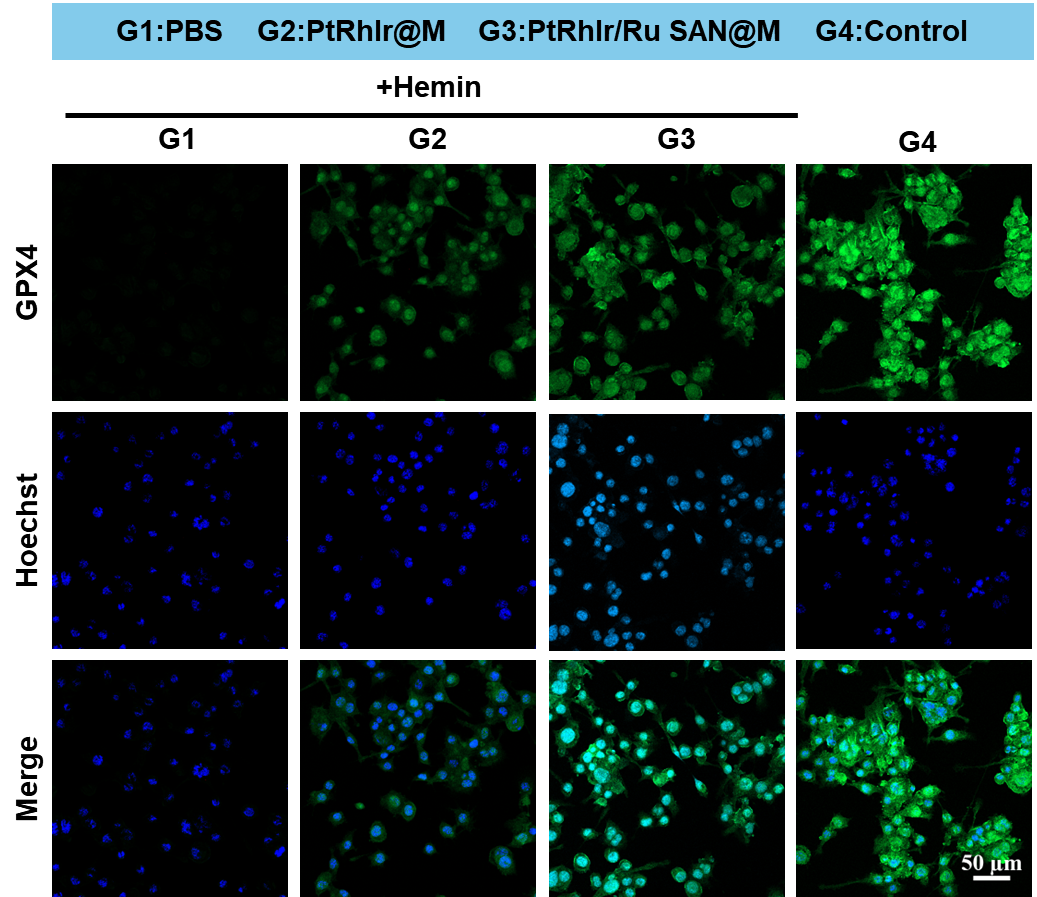


**Figure S23**. CLSM images of GPX4 staining in BV2 cells with different treatment.


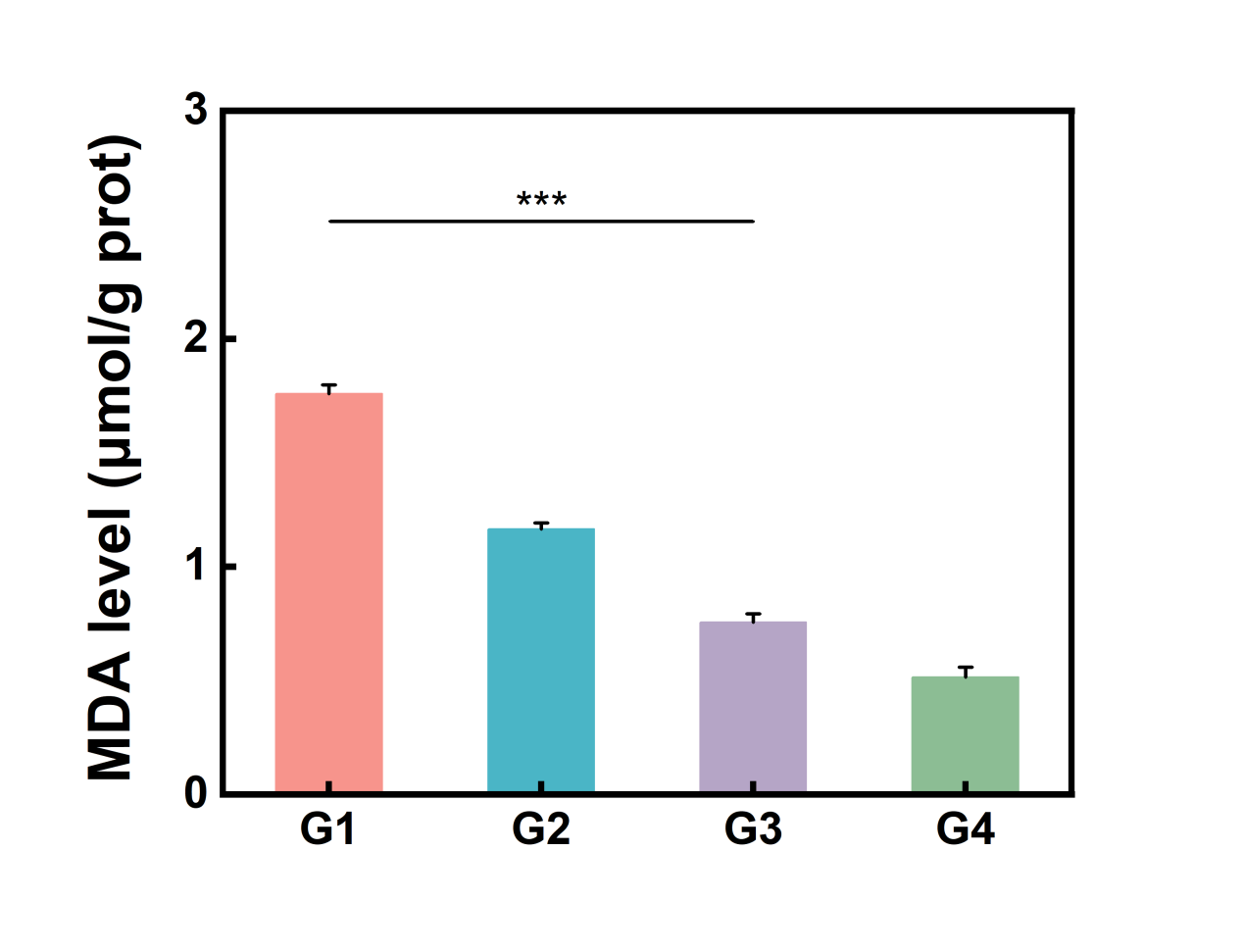


**Figure S24**. MDA detection in BV2 cells with different treatment. (n = 3, for each group). Statistical significance was assessed using a two tailed Student’s *t*-test. Data are presented as means ± SD. **p* < 0.05, ***p* < 0.01, ****p* < 0.001.


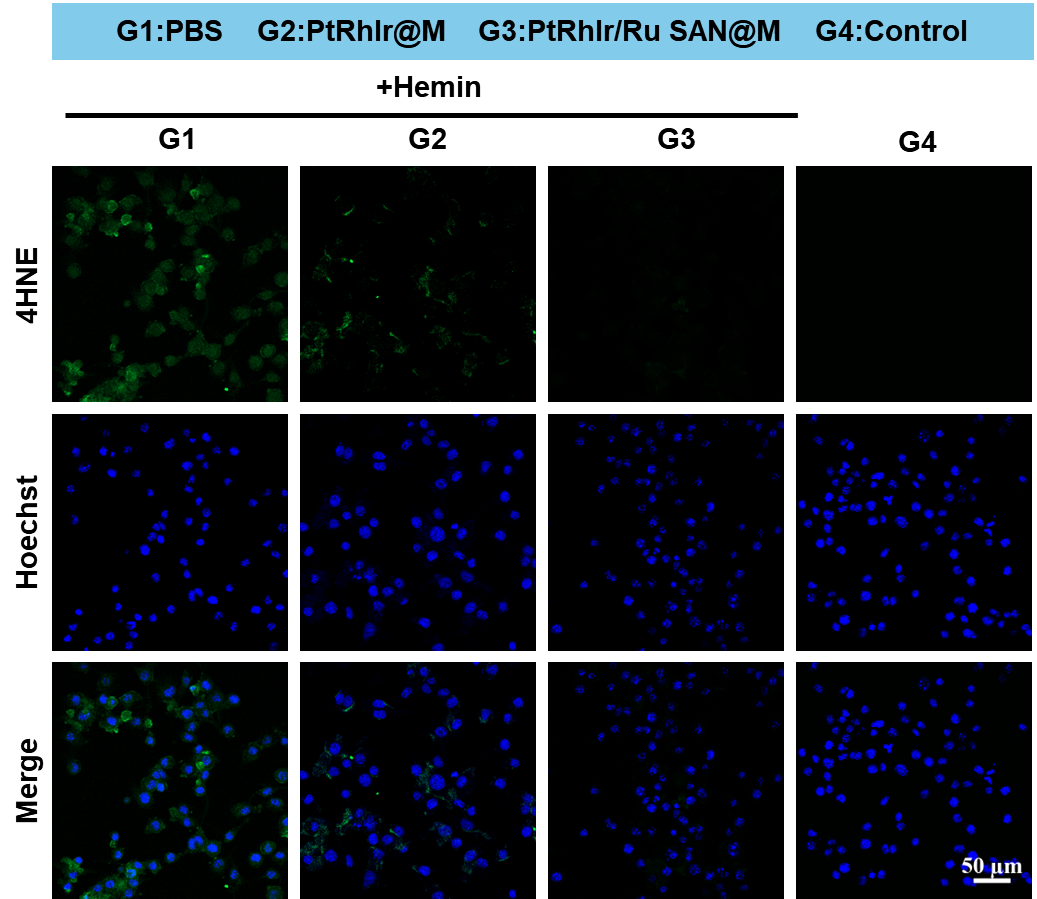


**Figure S25**. CLSM images of 4HNE staining in BV2 cells with different treatment.


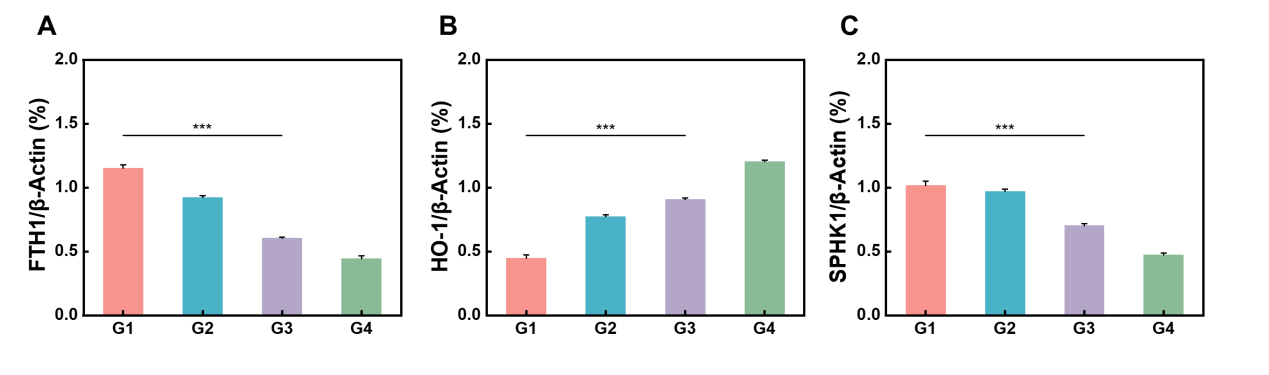


**Figure S26**. Corresponding quantification of the expression levels of A) FTH1, B) HO-1 and C) SPHK1 in BV-2 cells with different treatment. (n = 3, for each group). Statistical significance was assessed using a two tailed Student’s *t*-test. Data are presented as means ± SD. **p* < 0.05, ***p* < 0.01, ****p* < 0.001.


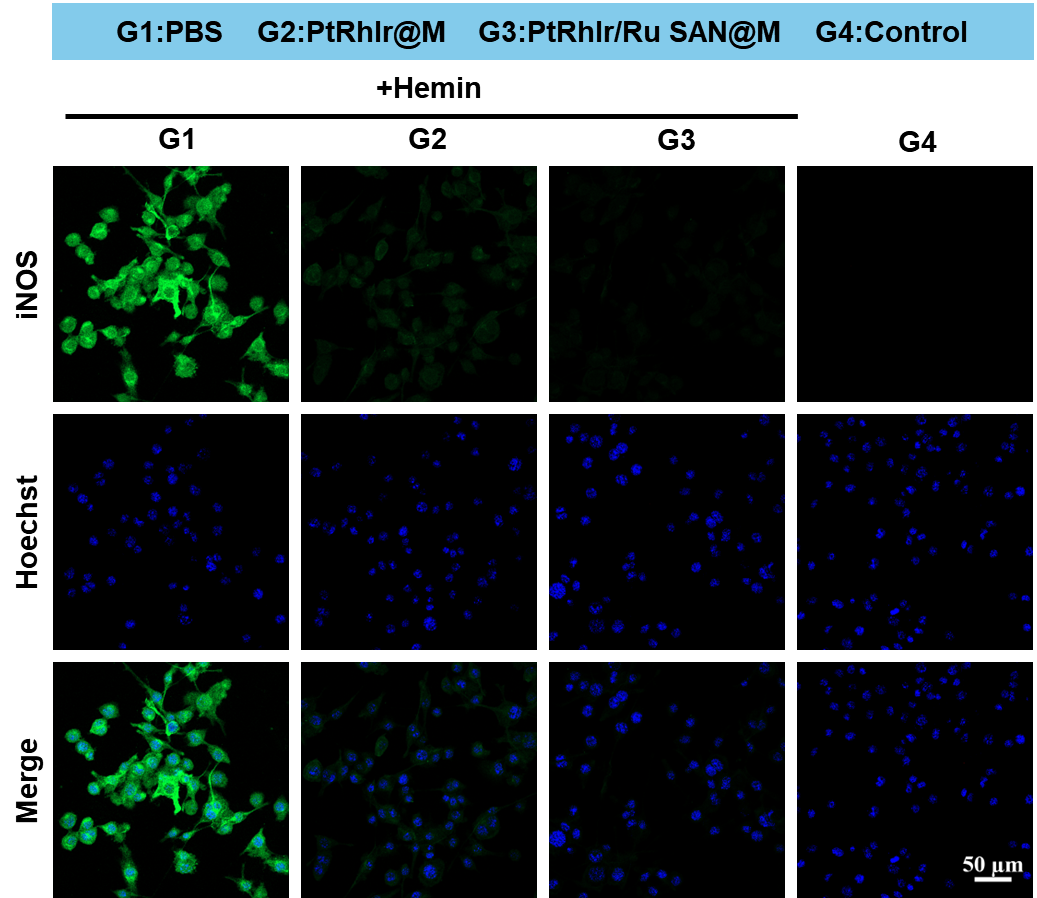


**Figure S27**. CLSM images of iNOS staining in BV2 cells with different treatment.


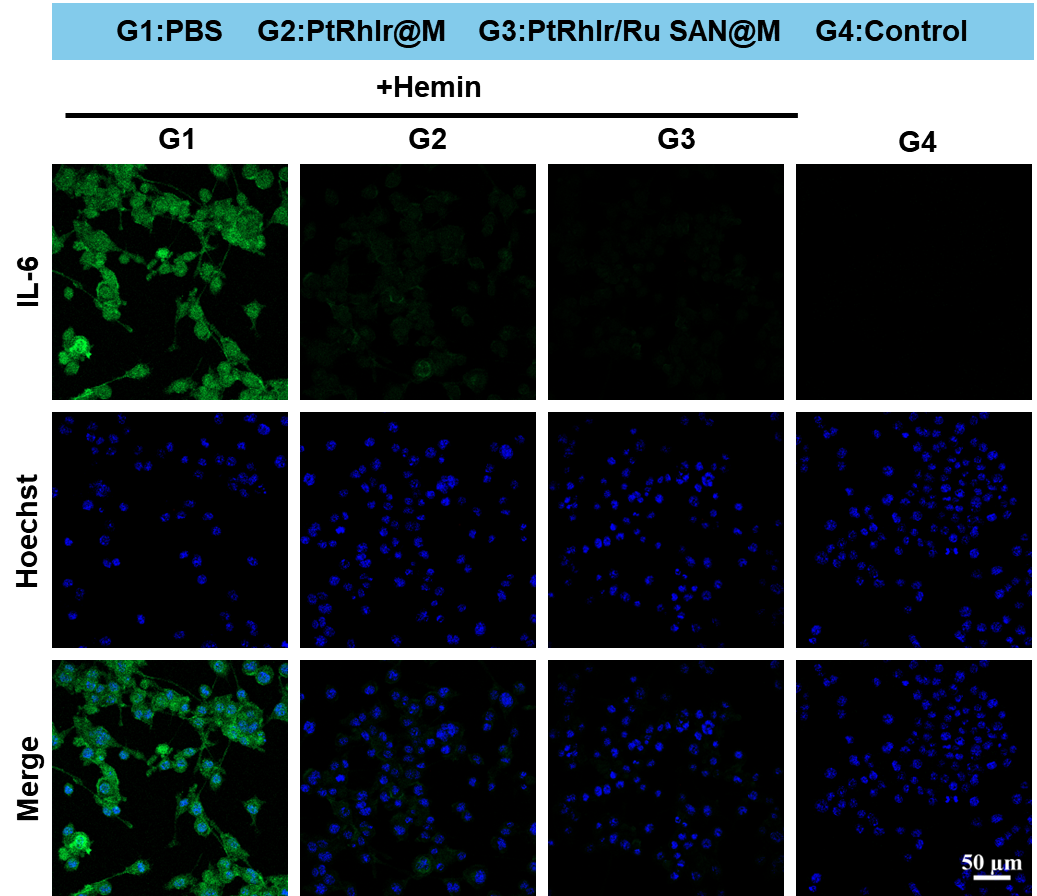


**Figure S28**. CLSM images of IL-6 staining in BV2 cells with different treatment.


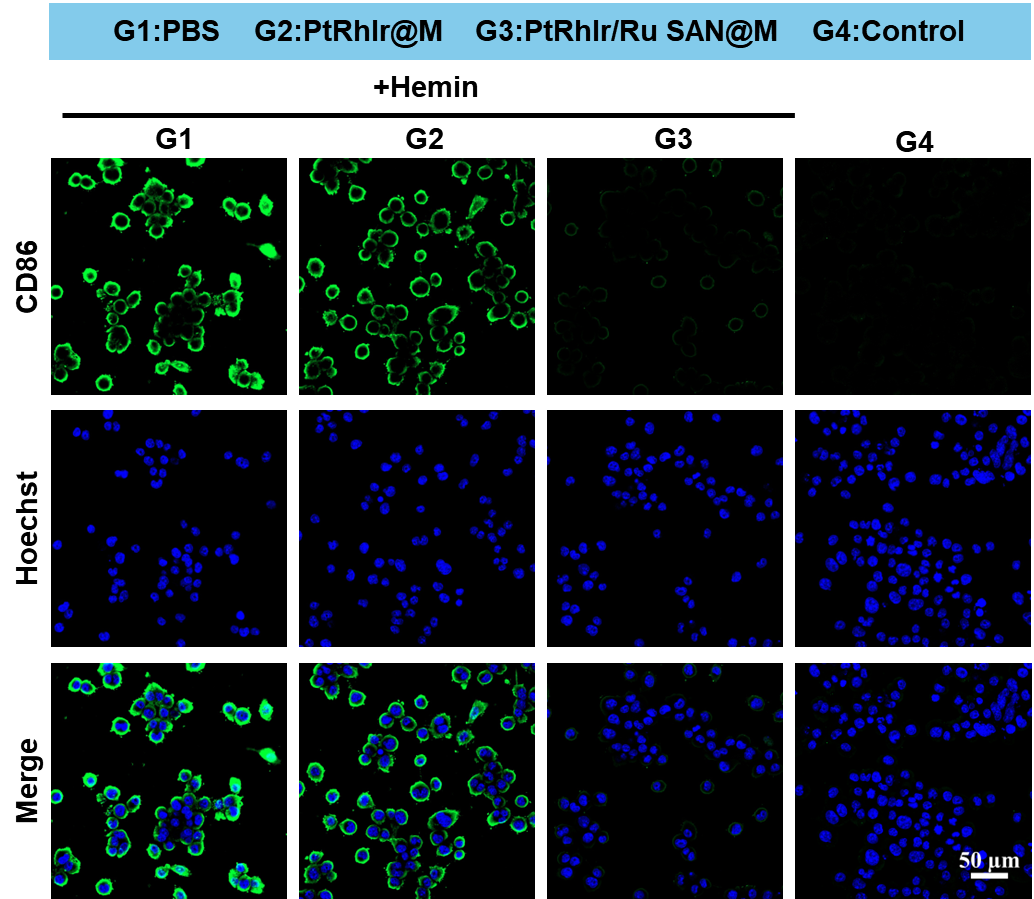


**Figure S29**. CLSM images of CD86 staining in BV2 cells with different treatment.


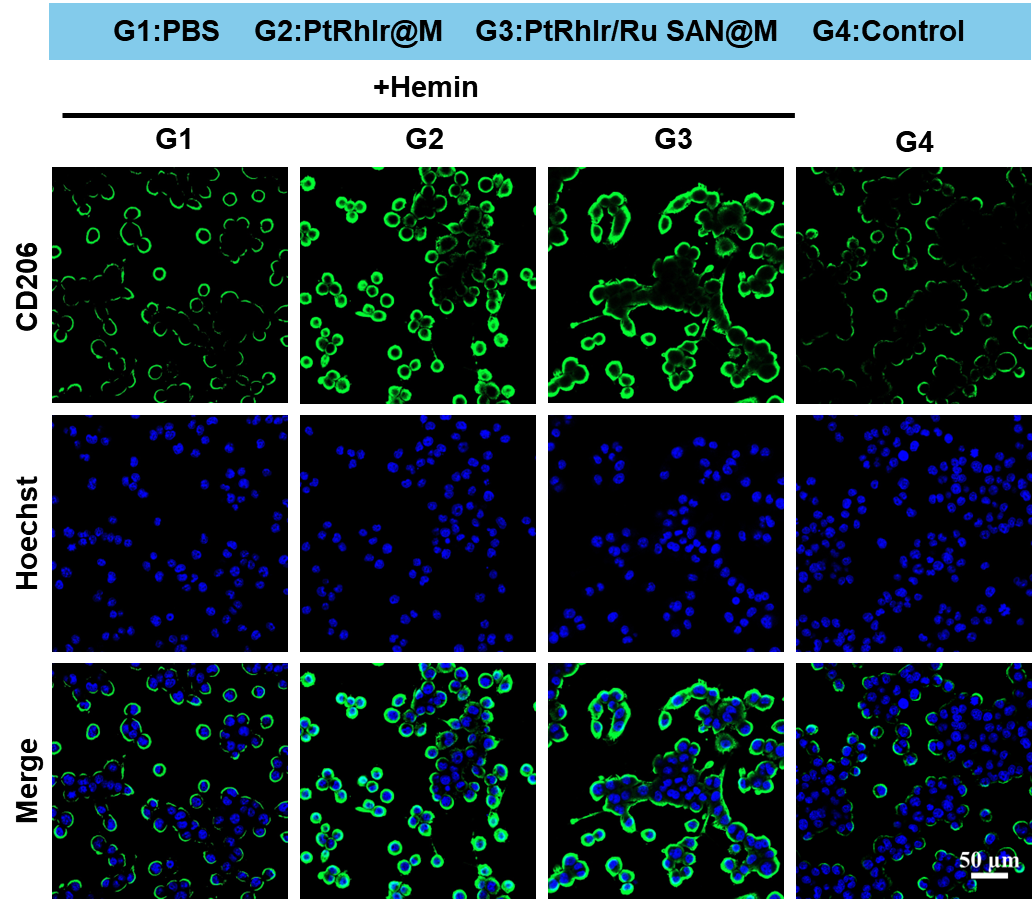


**Figure S30**. CLSM images of CD206 staining in BV2 cells with different treatment.


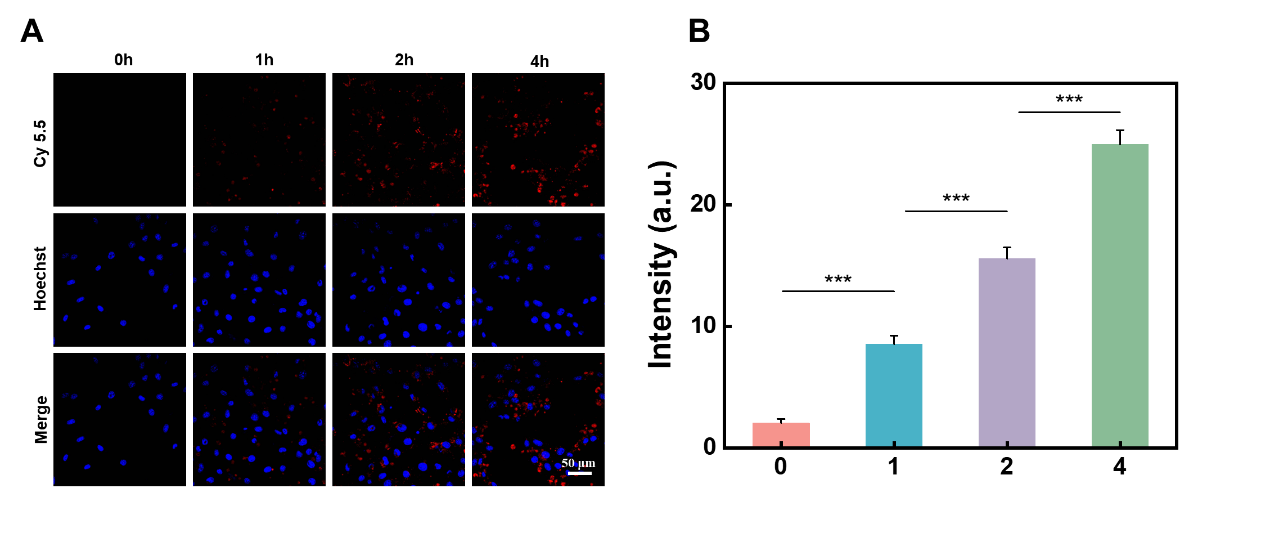


**Figure S31**. A) CLSM images showing cellular uptake and B) corresponding quantification of fluorescence intensity in HT22 cells. (n = 3, for each group). Statistical significance was assessed using a two tailed Student’s *t*-test. Data are presented as means ± SD. **p* < 0.05, ***p* < 0.01, ****p* < 0.001.


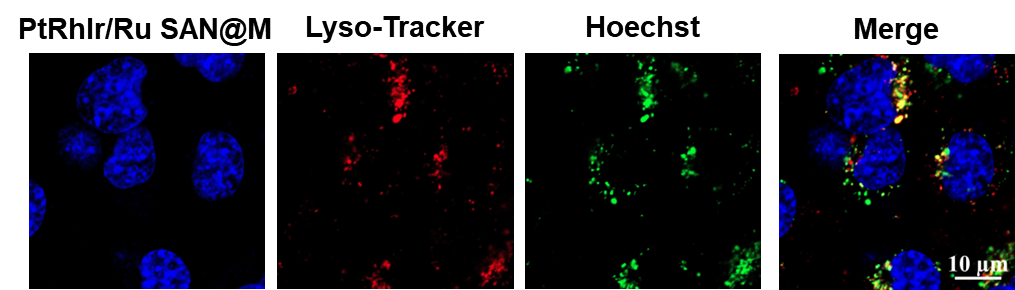


**Figure S32**. CLSM images of colocalization between the lysosome tracker and PtRhIr/Ru SAN@M in HT22 cells.


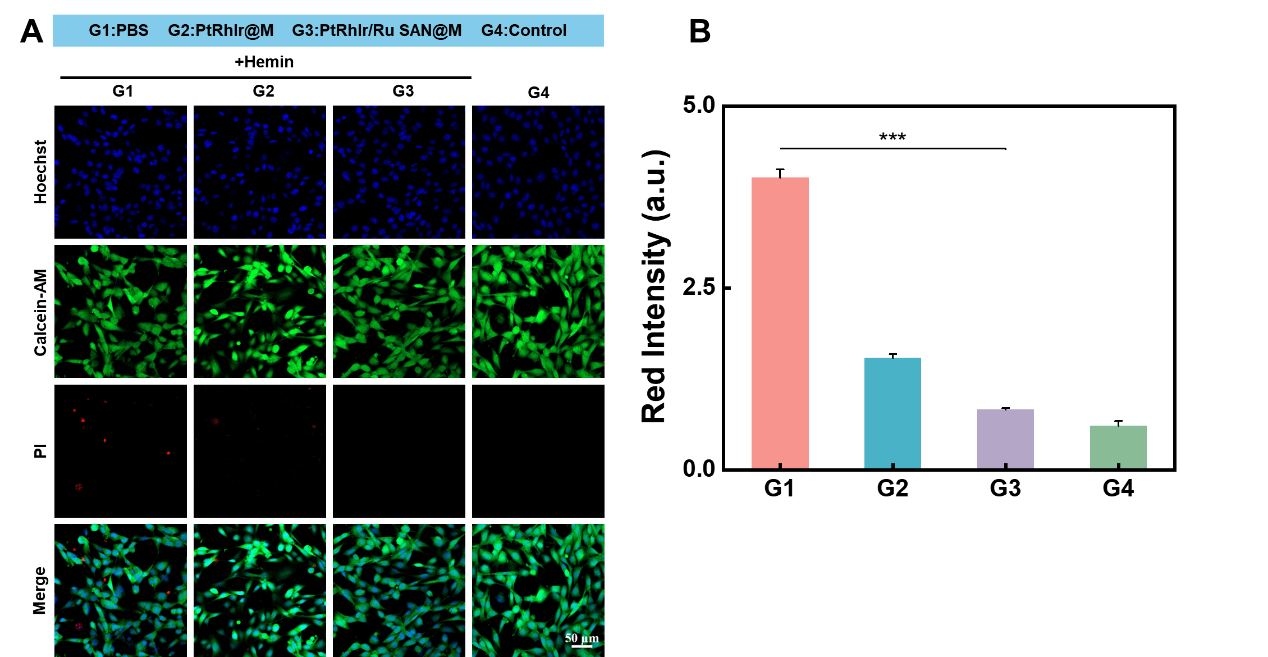


**Figure S33**. A) CLSM images depicting Calcein-AM/PI co-staining and B) corresponding quantification of fluorescence intensity in HT22 cells with different treatment. (n = 3, for each group). Statistical significance was assessed using a two tailed Student’s *t*-test. Data are presented as means ± SD. **p* < 0.05, ***p* < 0.01, ****p* < 0.001.


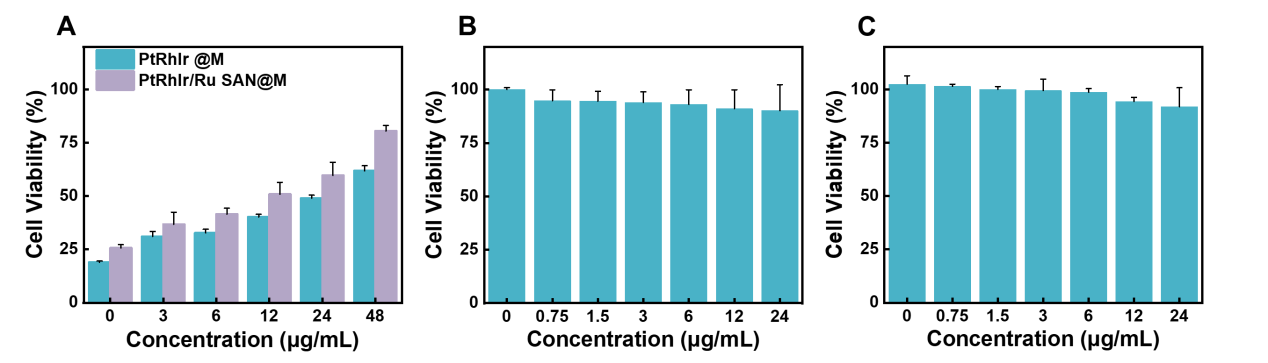


**Figure S34**. A) Cell viability of Hemin-induced BV-2 cells following 24 h treatment with various concentrations of PtRhIr@M and PtRhIr/Ru SAN. Cell viability of BV2 cells treated with different concentrations of B) PtRhIr/Ru SAN@M and C) PtRhIr@M. (n = 4, for each group) Data are presented as means ± SD.


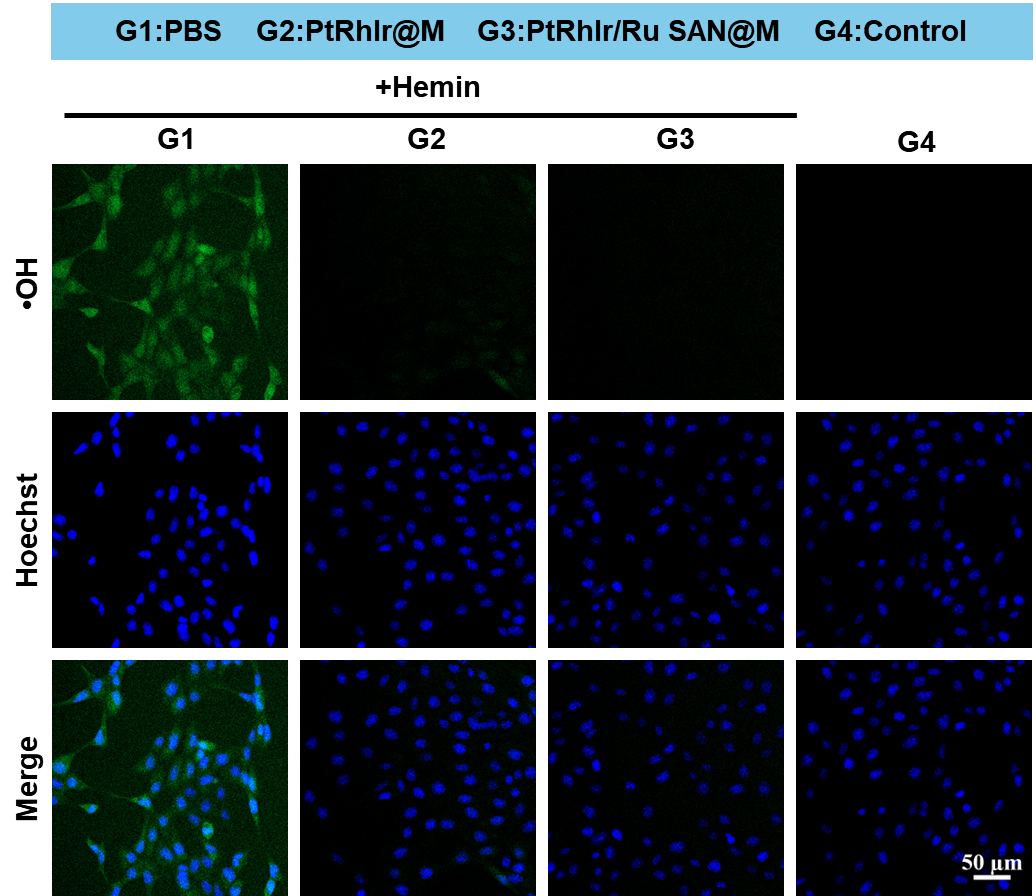


**Figure S35**. CLSM images of •OH staining in HT22 cells with different treatment.


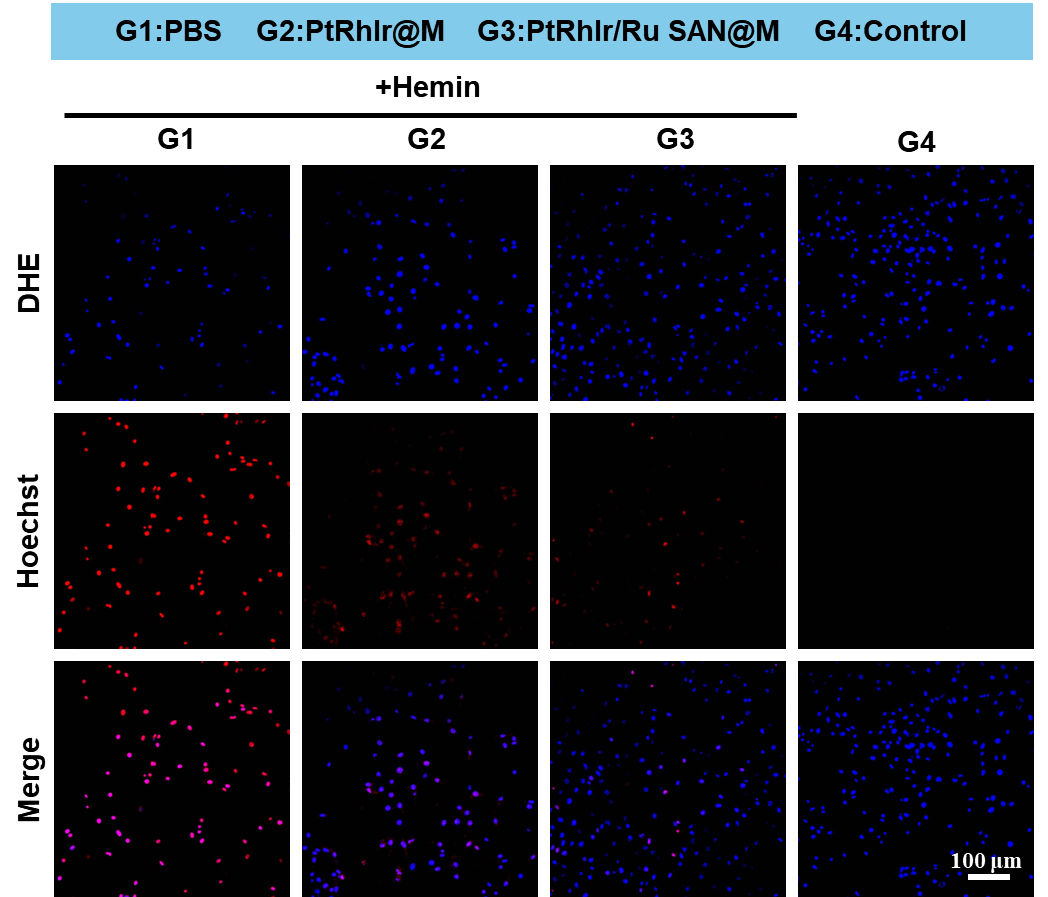


**Figure S36**. CLSM images of DHE staining in HT22 cells with different treatment.


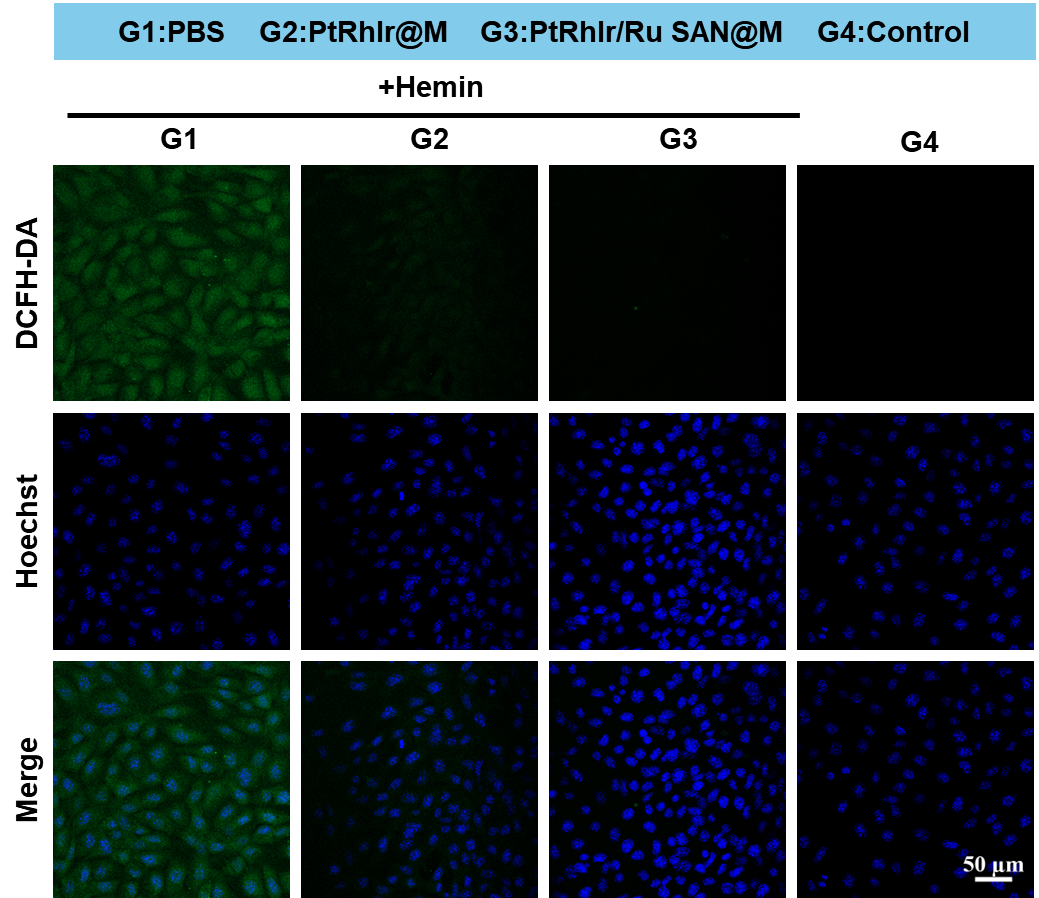


**Figure S37**. CLSM images of ROS staining in HT22 cells with different treatment.


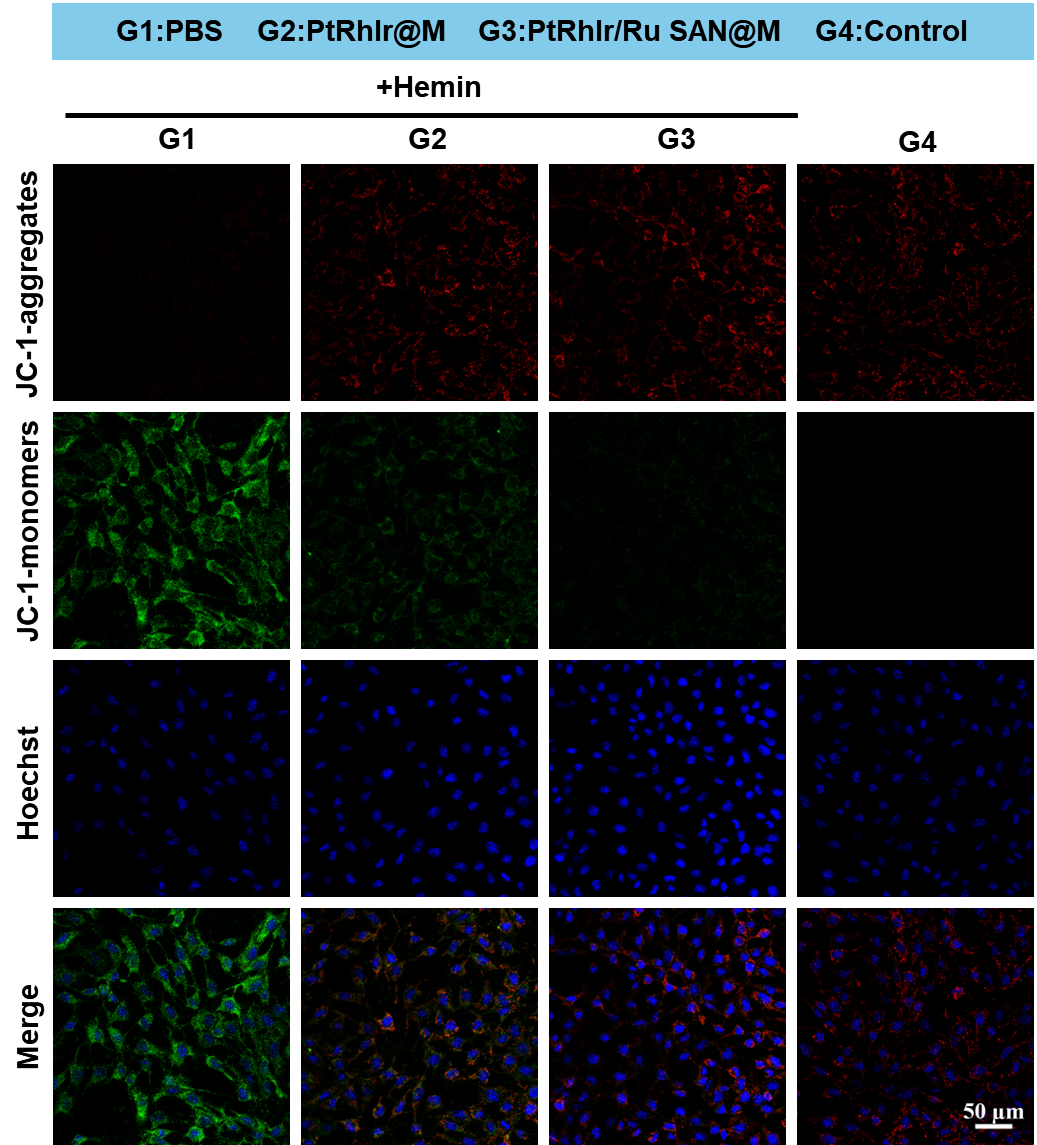


**Figure S38**. CLSM images of JC-1 staining in HT22 cells with different treatment.


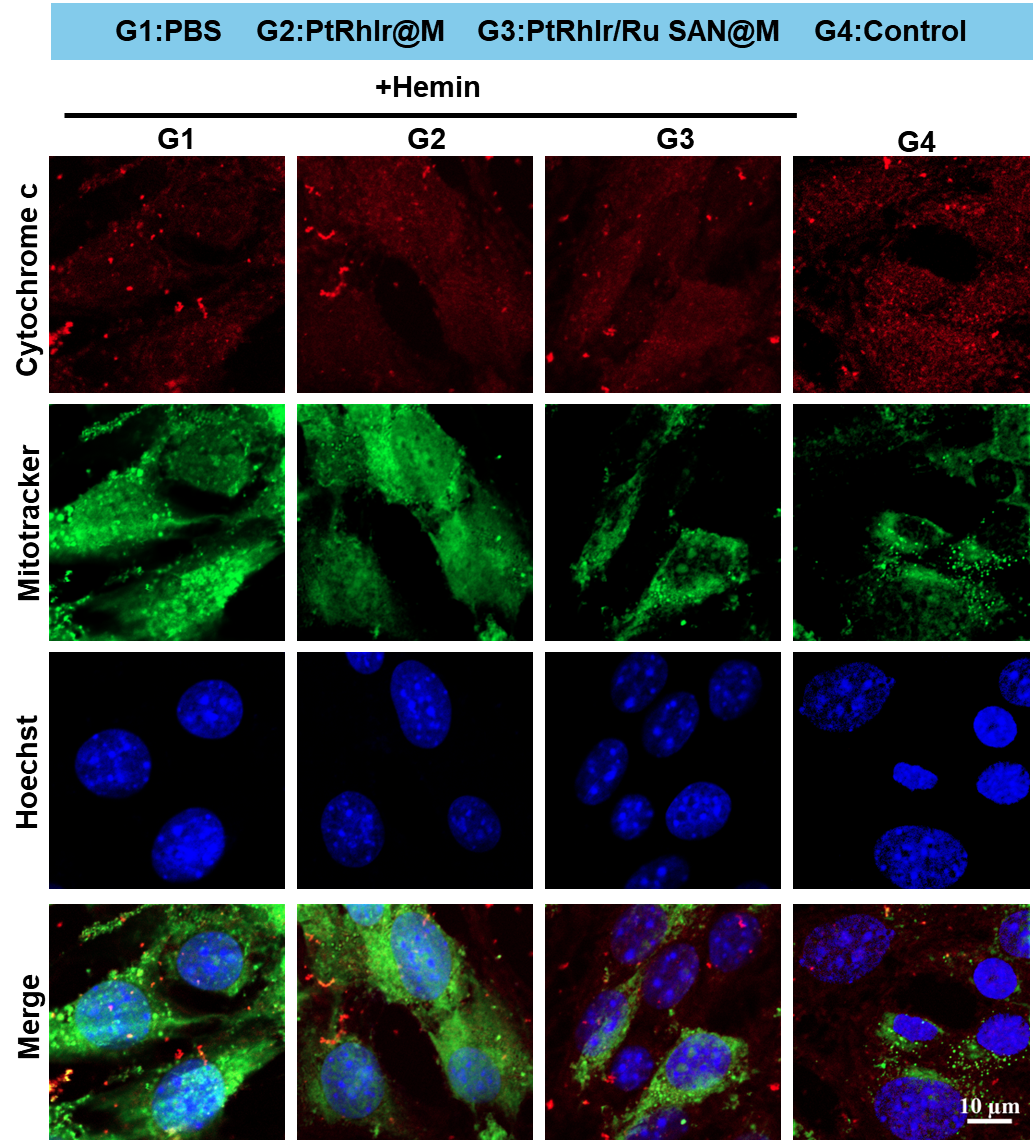


**Figure S39**. CLSM images of Cyt c staining in HT22 cells with different treatment.


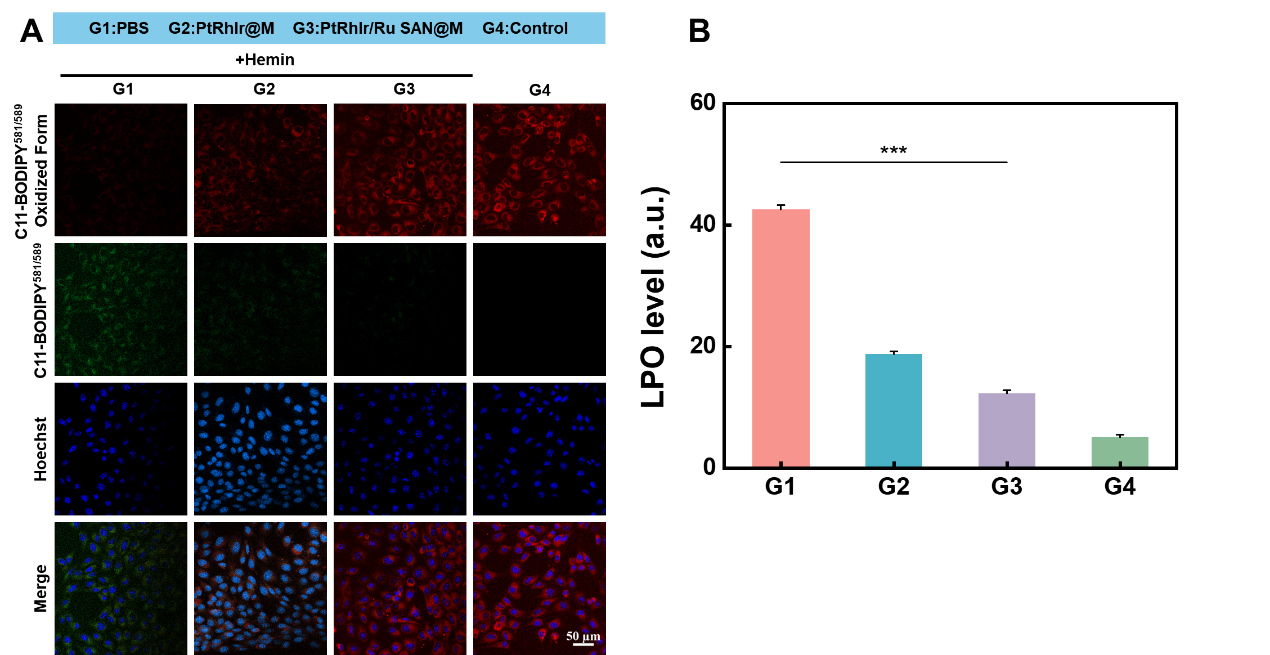


**Figure S40**. A) CLSM images and B) corresponding quantification of LPO staining fluorescence intensity in HT22 cells with different treatments. (n = 3, for each group). Statistical significance was assessed using a two tailed Student’s *t*-test. Data are presented as means ± SD. **p* < 0.05, ***p* < 0.01, ****p* < 0.001.


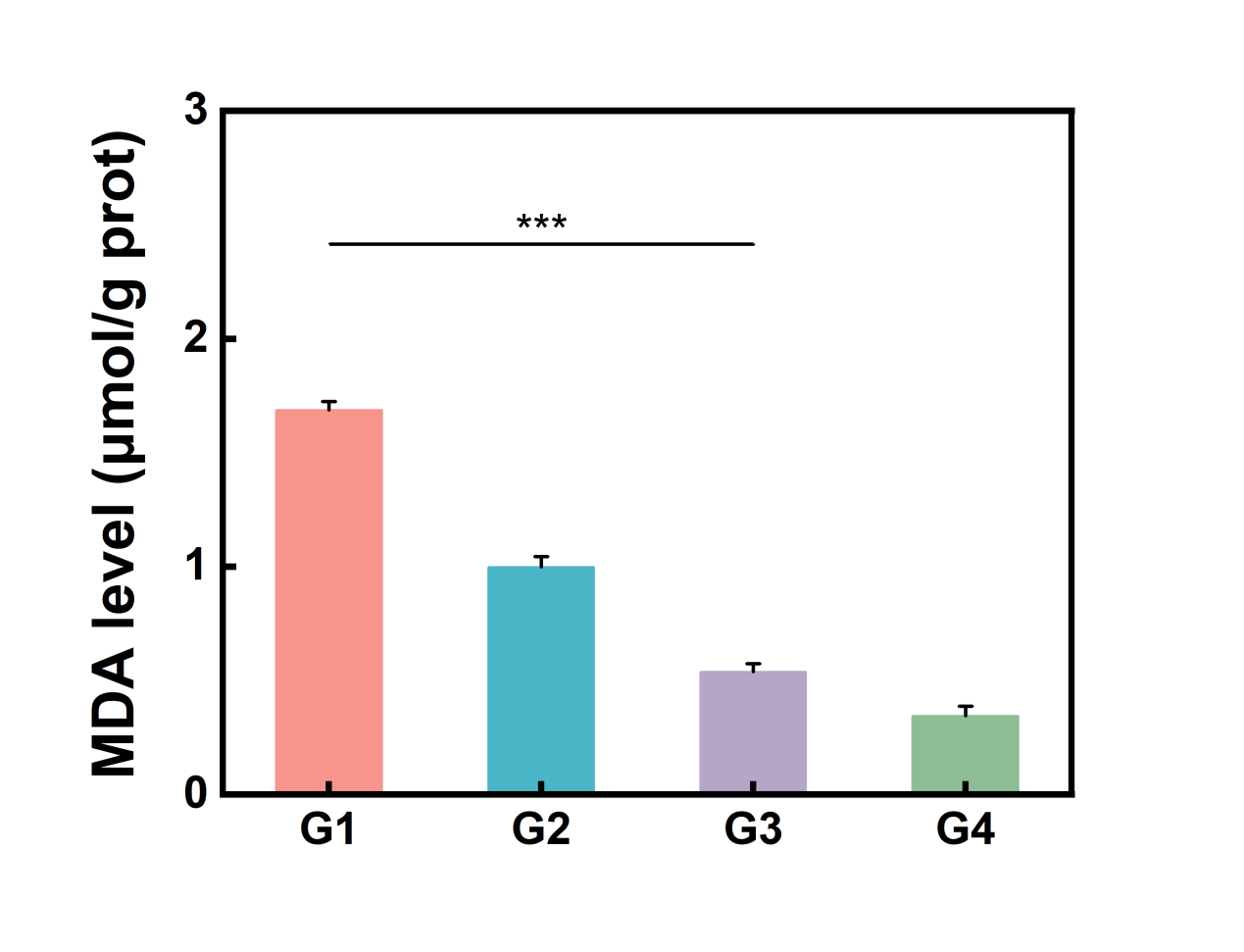


**Figure S41**. MDA detection in HT22 cells with different treatment. (n = 3, for each group). Statistical significance was assessed using a two tailed Student’s *t*-test. Data are presented as means ± SD. **p* < 0.05, ***p* < 0.01, ****p* < 0.001.


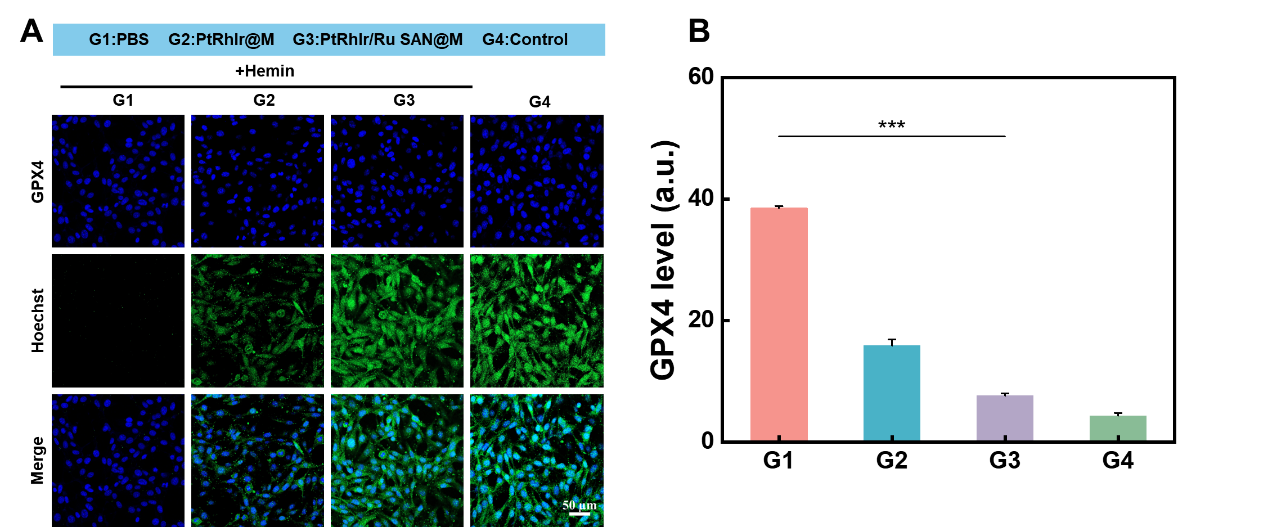


**Figure S42**. A) CLSM images and B) corresponding quantification of GPX4 staining fluorescence intensity in HT22 cells with different treatments. (n = 3, for each group). Statistical significance was assessed using a two tailed Student’s *t*-test. Data are presented as means ± SD. **p* < 0.05, ***p* < 0.01, ****p* < 0.001.


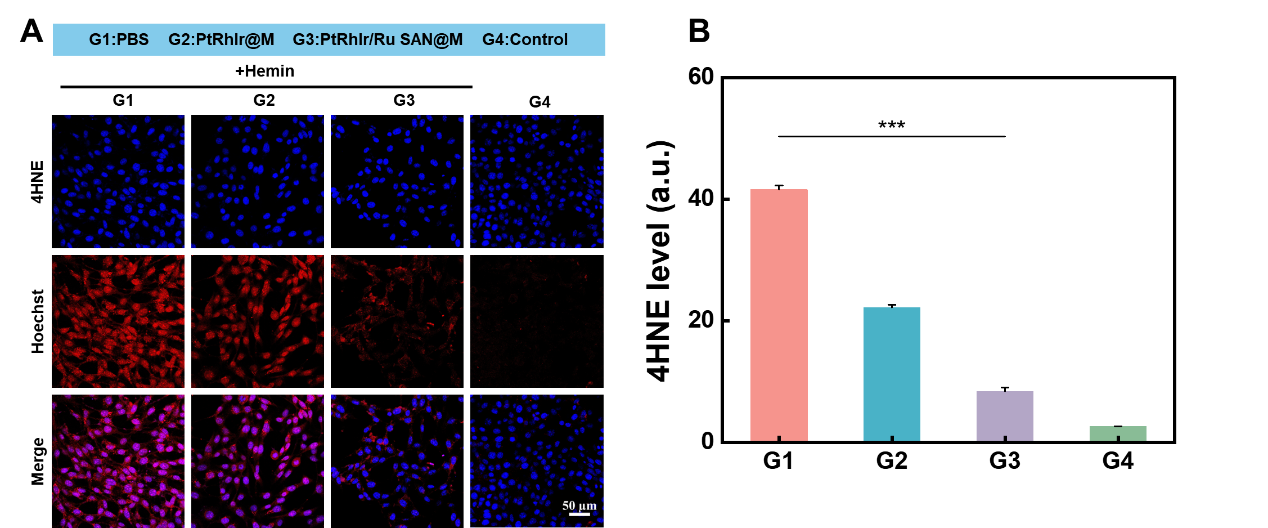


**Figure S43**. A) CLSM images and B) corresponding quantification of 4HNE staining fluorescence intensity in HT22 cells with different treatments. (n = 3, for each group). Statistical significance was assessed using a two tailed Student’s *t*-test. Data are presented as means ± SD. **p* < 0.05, ***p* < 0.01, ****p* < 0.001.


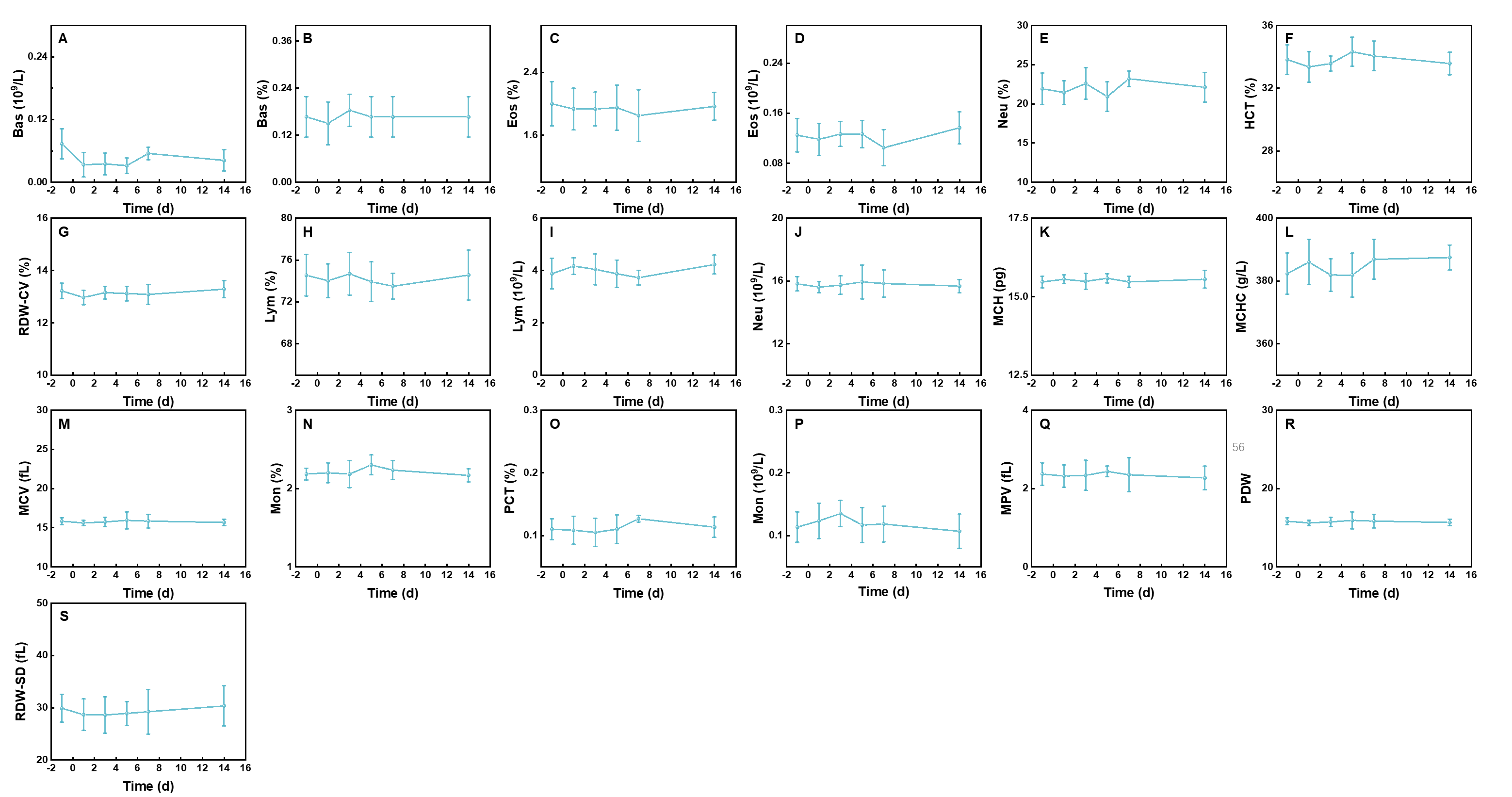


**Figure S44**. Hematological analysis of mice from different groups: A) Bas (10^9^/L), B) Bas (%), C) Eos (%), D) Eos (10^9^/L), E) Neu (%), F) HCT (%), G) RDW-CV (%), H) Lym (%), I) Lym (10^9^/L), J) Neu (10^9^/L), K) MCH (pg), L) MCHC (g/L), M) MCV (fL), N) Mon (%), O) PCT (%), P) Mon (10^9^/L), Q) MPV (fL), R) PDW, S) RDW-SD (fL). (n = 3, for each group). Data are presented as means ± SD.


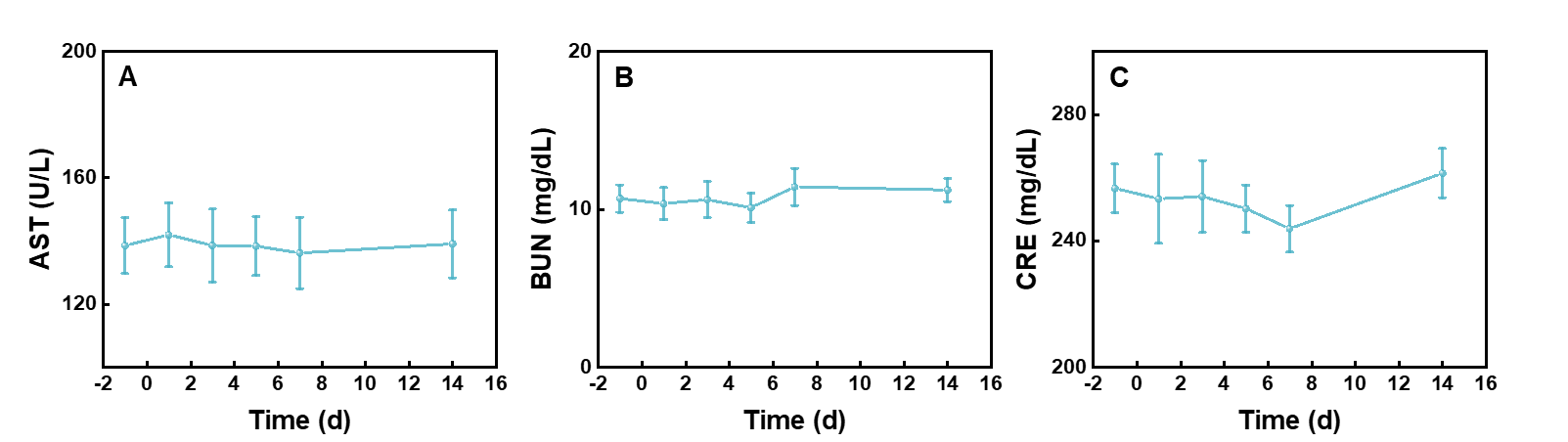


**Figure S45**. Analysis of liver and renal functions in mice from different groups: A) AST, B) BUN, and C) CRE. (n = 3, for each group). Data are presented as means ± SD.


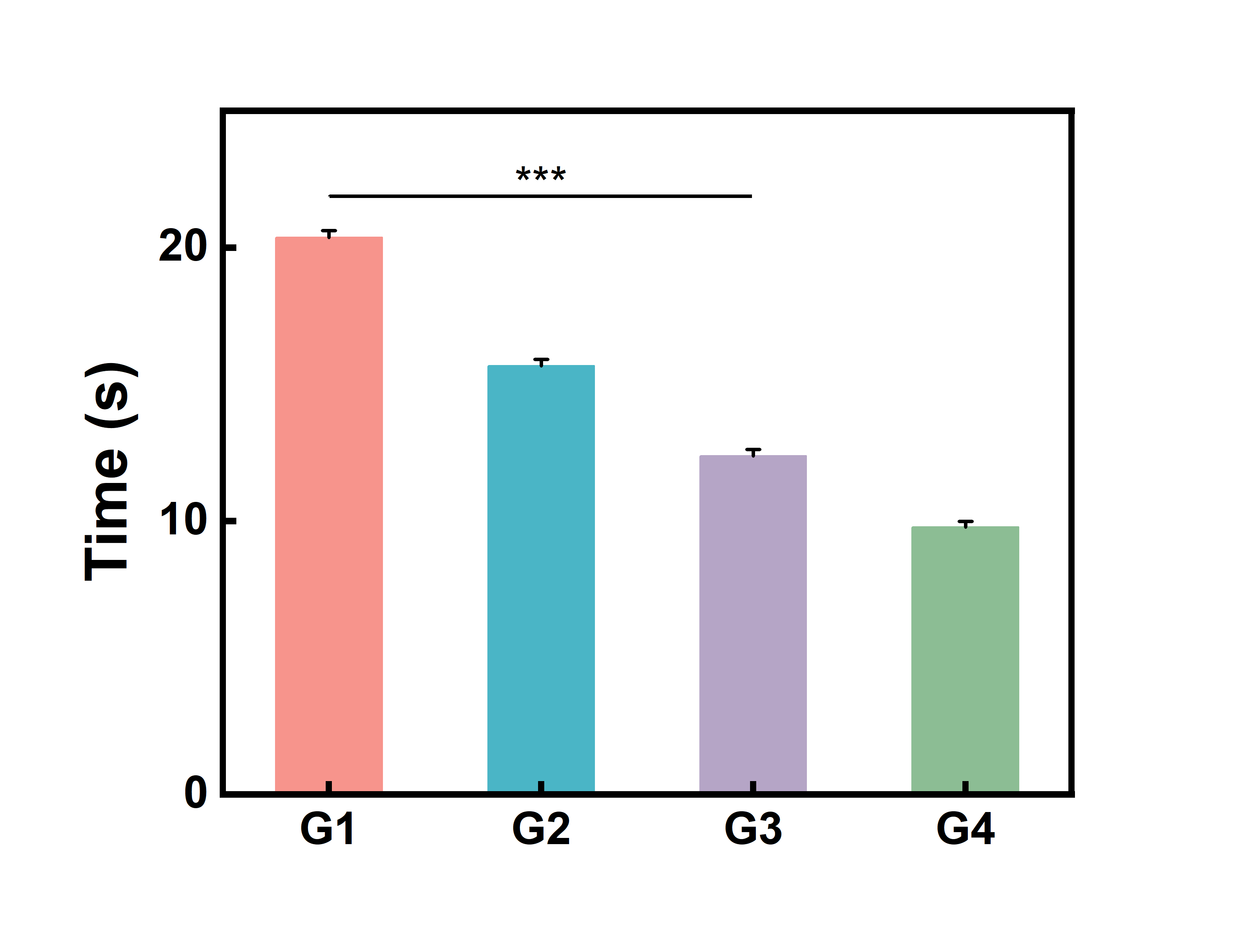


**Figure S46**. Beam walking test: time to endpoint in different groups. (n = 6, for each group). Statistical significance was assessed using a two tailed Student’s *t*-test. Data are presented as means ± SD. **p* < 0.05, ***p* < 0.01, ****p* < 0.001.


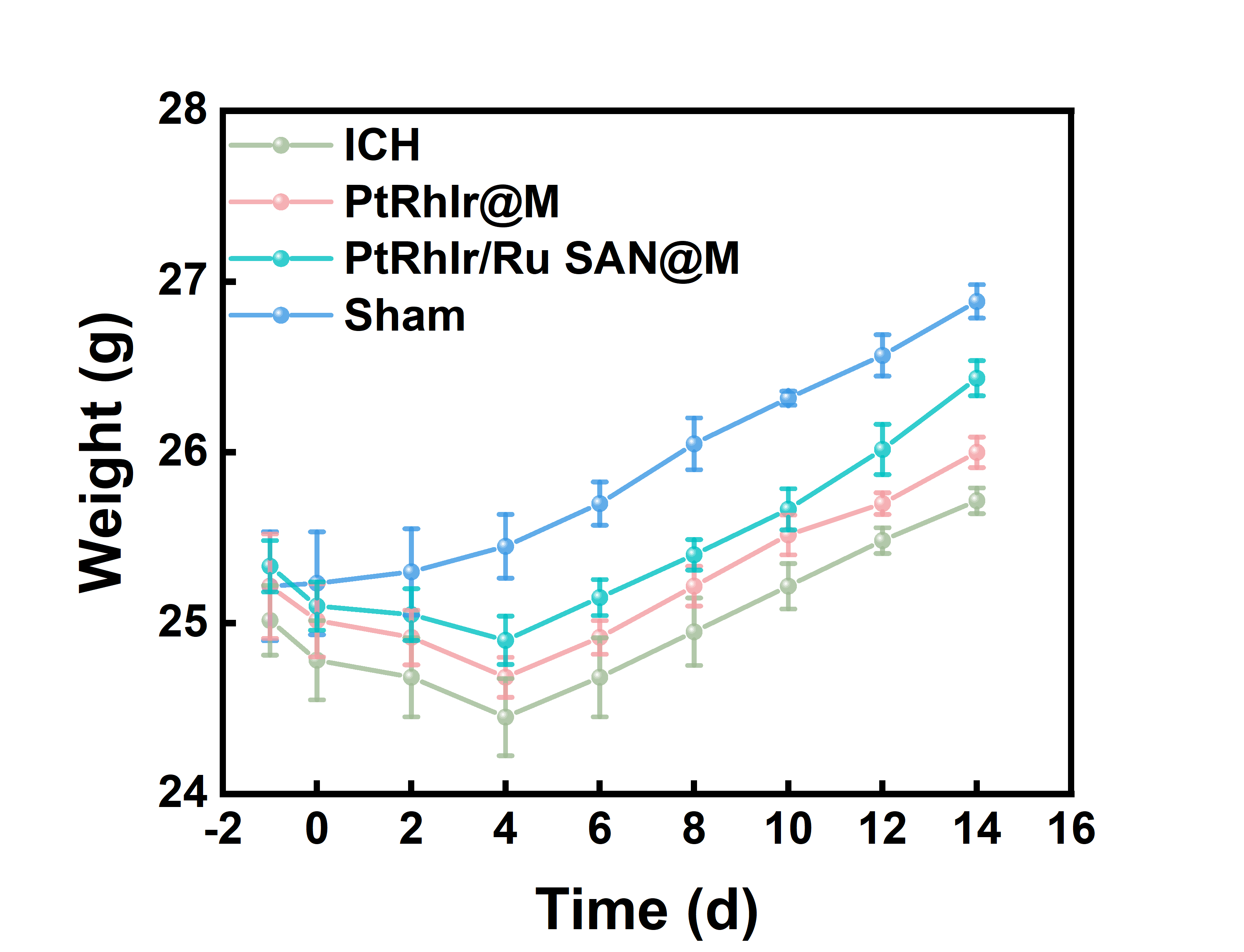


**Figure S47**. Mice weight changes with different treatments. (n = 6, for each group). Data are presented as means ± SD.


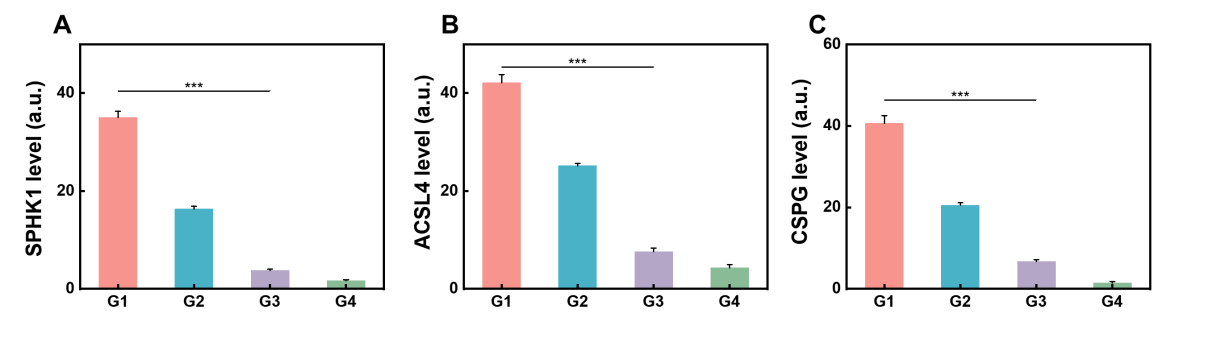


**Figure S48**. Corresponding fluorescence intensities of A) SPHK1, B) ACSL4, and C) CSPG are shown. (n = 3, for each group). Statistical significance was assessed using a two tailed Student’s *t*-test. Data are presented as means ± SD. **p* < 0.05, ***p* < 0.01, ****p* < 0.001.
